# Au(I) Catalyzed HF Transfer: Tandem Alkyne Hydrofluorination and Perfluoroarene Functionalization

**DOI:** 10.1021/acscatal.1c05474

**Published:** 2022-03-01

**Authors:** Daniel Mulryan, Jack Rodwell, Nicholas A. Phillips, Mark R. Crimmin

**Affiliations:** Molecular Sciences Research Hub, Imperial College London, 82 Wood Lane, Shepherds Bush, London W12 0BZ, U.K.

**Keywords:** hydrofluorination, vinyl fluorides, gold catalysis, shuttle catalysis, fluoroarene

## Abstract

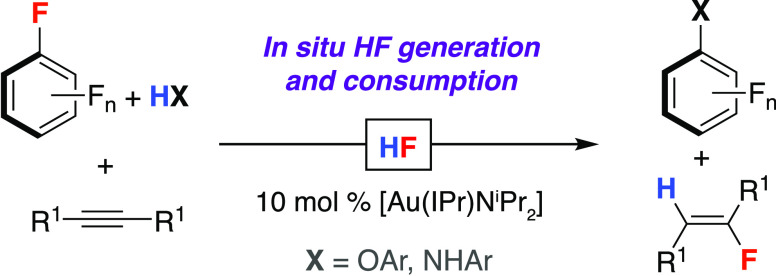

HF transfer reactions between organic substrates are potentially useful transformations. Such reactions require the development of catalytic systems that can promote both defluorination and fluorination steps in a single reaction sequence. Herein, we report a catalytic protocol in which an equivalent of HF is generated from a perfluoroarene | nucleophile pair and transferred directly to an alkyne. The reaction is catalyzed by [Au(IPr)N^i^Pr_2_] (IPr = *N*,*N*′-1,3-bis(2,6-diisopropylphenyl)imidazol-2-ylidene). HF transfer generates two useful products in the form of functionalized fluoroarenes and fluoroalkenes. Mechanistic studies (rate laws, KIEs, density functional theory (DFT) calculations, competition experiments) are consistent with the Au(I) catalyst facilitating a catalytic network involving both concerted S_N_Ar and hydrofluorination steps. The nature of the nucleophile impacts the turnover-limiting step. The cS_N_Ar step is turnover-limiting for phenol-based nucleophiles, while protodeuaration likely becomes turnover-limiting for aniline-based nucleophiles. The approach removes the need for direct handling of HF reagents in hydrofluorination and offers possibilities to manipulate the fluorine content of organic molecules through catalysis.

## Introduction

Hydrofluorination is an essential method in synthesis. The addition of HF to unsaturated functional groups serves as an atom-efficient and expedient way to introduce fluorine atoms into organic molecules.^1−10^ Such substitutions are highly attractive for drug discovery and agrochemical sciences where the introduction of fluorine is known to block metabolic pathways, improve lipophilicity, modify p*K*_a_ of adjacent sites, and improve binding through noncovalent interactions.^11,12^

Hydrofluorination methods are not without their technical challenges. HF is a corrosive gas, and high concentrations can be fatal in contact with skin.^13,14^ Modified HF reagents, such as pyridinium poly(hydrogen fluoride) (Olah’s reagent) or triethylamine tri(hydrogen fluoride) (TREAT-HF), have been widely adopted, and while not volatile like HF itself, they remain highly toxic and corrosive.^8,15,16^ Furthermore, these types of fluorinating agents are exclusively derived from HF produced from acidification of fluorite (CaF_2_). There are concerns over the sustainability of such approaches.^17,18^ In the long term, the fluorochemicals sector will need to resolve the twin issues of finite raw materials and the damage caused by the release of fluorinated molecules into the environment.

**Figure 1:**
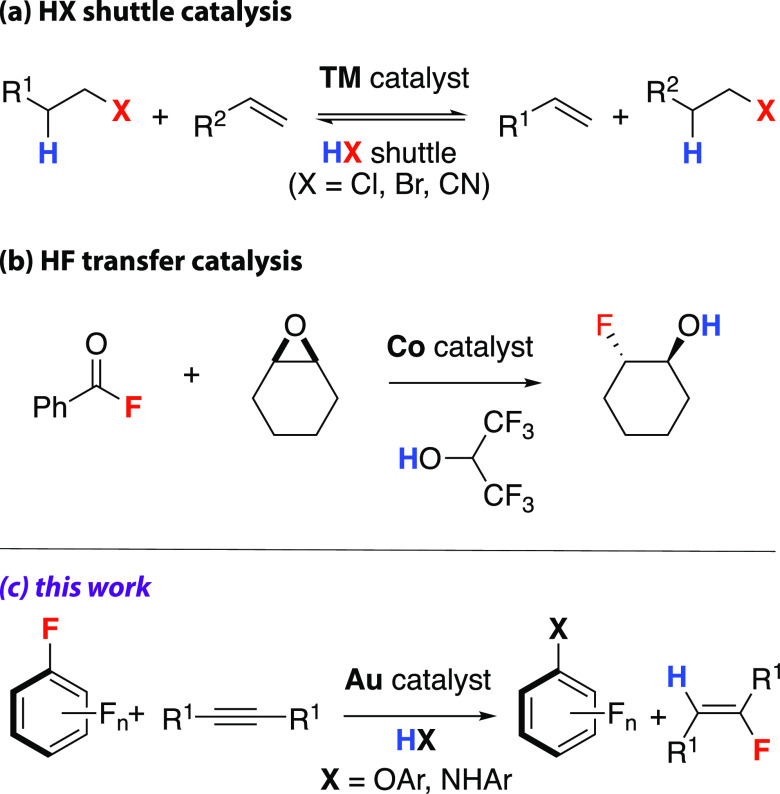
(a) General reaction scheme for transition metal-catalyzed HX shuttling (X = Cl, Br, CN). (b) HF transfer catalysis. (c) This work.

In this paper, we report a new catalytic reaction that results in the transfer of an equivalent of HF between a fluoroarene | nucleophile pair and an alkyne. Transfer functionalizations are an emerging, highly efficient, and powerful class of reactions for synthesis, owing to their high atom economy and potential reversibility.^19,20^ Recent advances in this field have provided systems for the shuttling of HX (Figure 1a, X = CN,^21^ Cl, Br,^22^ I^23^) between organic fragments.^24^ Our approach allows the realization of these methods for HF transfer and combines both defluorination and hydrofluorination steps in a single catalytic network. Both products of HF transfer are useful fluorinated synthons, resulting in a highly economic process. The new method removes the need to directly handle HF (or related) reagents in hydrofluorination catalysis, improving safety concerns. It also represents an important step toward the chemical recycling of fluorinated compounds through the reuse of their fluorine content.^25^

There is limited precedent for reactions that transfer HF between organic substrates. In 2010, Kalow and Doyle reported the catalytic enantioselective reaction of benzoyl fluoride, 1,1,1,3,3,3-hexafluoro-2-propanol, and cyclohexene oxide (Figure 1b).^26−29^ The reaction resulted in the net addition of HF to the epoxide. Despite this remarkable result, a general approach to HF transfer for hydrofluorination remains elusive. Indeed, transition metal catalysts developed for HX shuttling (X = CN, Cl, Br, I)^21−23^ are poor candidates to develop such reactivity due to the reluctance of carbon–fluorine bonds to participate in oxidative addition and reductive elimination steps at transition metal centers.

In 2006, Sadighi and co-workers reported that the gold(I) fluoride complex, [Au(SIPr)F], could catalyze the *trans*-selective addition of HF to an internal alkyne (SIPr = 1,3-*bis*(2,6-diisopropylphenyl)-4,5-dihydroimidazol-2-ylidene).^1^ A gold(I) fluoride complex supported by a *bis*(phosphine) ligand, [Au(^t^BuXantPhos)F] has also been shown to be an on-cycle intermediate in the catalytic S_N_Ar of perfluoroarenes (^t^BuXantPhos = 9,9-dimethyl-4,5-bis(di-tertbutylphosphino)xanthene).^30^ Both pathways achieve catalytic turnover due to the low fluorophilicity of Au(I). These two key results suggest that Au(I) catalysts may be viable candidates for developing transfer catalysis with HF but only if both types of reactivity can be established within a single catalytical network, ideally by a single catalyst.

## Results and Discussion

### Catalyst Development

Following a campaign of catalyst screening and reaction optimization, a new catalytic HF transfer reaction was developed (see Supporting Information for further details). The reaction of pentafluoropyridine, 4-methoxyphenol, and diphenylacetylene in toluene at 120 °C was catalyzed by 10 mol % [Au(IPr)N^i^Pr_2_]^31^ and led to the formation of corresponding biaryl ether (**1a**) and fluoroalkene (**2a**) in >80% spectroscopic yield. This protocol transfers an equivalent of HF from the fluoroarene | nucleophile pair to the alkyne. Hydrofluorination of the alkyne shows complete selectivity for the *trans*-isomer.^1,8^ The precatalyst is operationally simple. While a related Au(I) amide has been applied in hydrofluorination catalysis,^3^ to the best of our knowledge, these types of species are limited to stoichiometric applications in fluoroarene functionalization.^32^

Optimization of the conditions highlighted the need for the reaction to be performed in a poly(tetrafluoroethylene) lined vessel to exclude the side reactions with borosilicate glassware. The limiting reagent of the reaction is the fluoroarene. This finding exemplifies the difference with traditional hydrofluorination reactions of alkynes, in which an excess of HF-reagent is typical.^1,9,33^ Here, an excess of alkyne and a slight excess of nucleophile were required for a high yield of fluoroalkene product. Control reactions in the absence of the catalyst show no HF transfer to the alkyne. Furthermore, a background reaction between 4-methoxyphenol and pentafluoropyridine showed no reaction after 16 h at 120 °C. These controls demonstrate that the Au(I) catalyst plays a role in both the hydrofluorination and S_N_Ar steps.

### Reaction Scope

**Figure 2:**
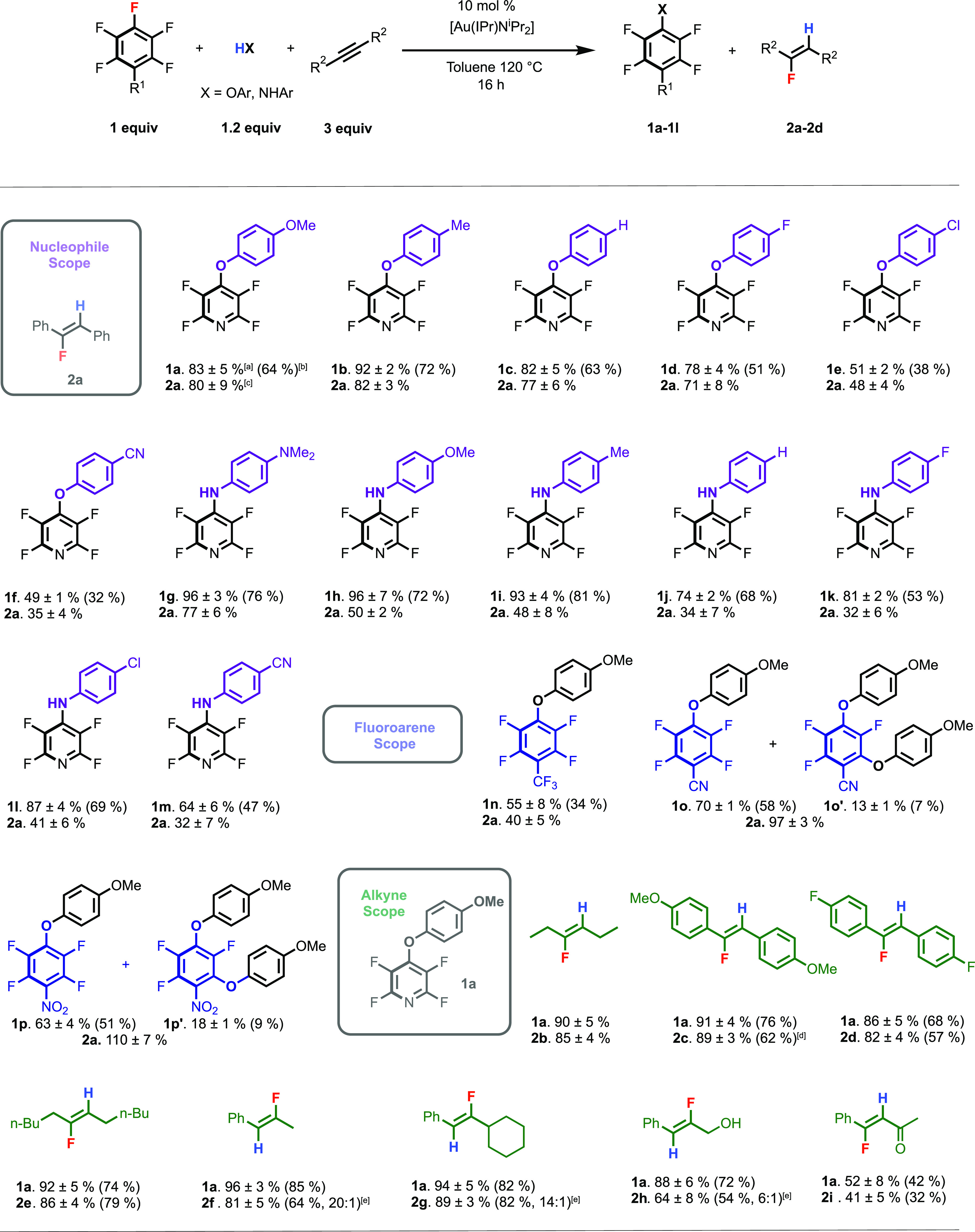
HF transfer reaction scope catalyzed by [Au(IPr)N^i^Pr_2_]. [a] Reactions were performed with 0.1:1:1.2:3 equivalents of catalyst: fluoroarene (0.04 M): nucleophile: alkyne. Yields of fluoroarene (**1a**-**1p**) and fluoroalkene (**2a**-**2d**) were calculated from ^19^F NMR spectroscopy using a fluorobenzene internal standard. Reactions were performed in triplicate, and standard deviations are reported with a 99% confidence level. [b] Isolated yields were obtained from scale-up reactions and are shown in parenthesis. [c] Isolated yields of **2a** are not reported due to this compound co-eluting with diphenylacetylene. [d] Due to the challenging isolation, this product was contaminated with ∼20% of unreacted alkyne. [e] Ratio of regioisomers β:α functionalization. Major isomer is shown.

A range of substituted phenols and anilines were shown to be effective nucleophiles for the reaction with pentafluoropyridine and diphenylacetylene to form **1a-m** (Figure 2). High to modest yields were observed with both electron-rich and electron-deficient nucleophiles; however, a general decrease in the relative yield of fluoroalkene product (**2a**) was observed with anilines compared to phenols. The scope in fluoroarene was investigated with 4-methoxyphenol as the nucleophile and diphenylacetylene as the HF acceptor to form mixtures of **1n-p** and **2a**. Lower yields were observed with less electron-deficient fluoroarenes, and the scope is currently limited to systems known to be susceptible to S_N_Ar. Di-substitution products **1o**′ and **1p**′ were observed with 2,3,4,5,6-pentafluorobenzonitrile and 2,3,4,5,6-pentafluoronitrobenzene allowing >1 equiv of HF to be liberated for each fluoroarene, thereby increasing the yield of the HF transfer product **2a**. Multiple S_N_Ar reactions are expected and are consistent with previous reports for these fluoroarenes.^34,35^ The reaction could also be applied to both symmetric and asymmetric alkyl and aryl internal alkynes, allowing the formation of **2b-i** as products of HF transfer. Hydrofluorination tolerates examples of both electron-donating and electron-withdrawing groups, including free alcohol. Attempts to expand the scope to terminal alkynes (e.g., hex-1-yne, ethynylbenzene) or silylated alkynes (e.g., trimethyl(phenylethynyl)silane or 1,2-bis(trimethysilyl)ethyne) did not lead to hydrofluorination products. In every case, the hydrofluorination was 100% *trans*-selective, and both the scope and selectivity parallels that reported by Sadighi and co-workers using HF-based reagents.^1,8^ Prior examples of Au(I) catalyzed fluoroarene functionalization are limited to the use of silylated nucleophiles due to the need to create a thermodynamic sink for the liberated fluoride.^30,32,36^ Hence, HF transfer catalysis allows expansion of the substrate scope to more convenient and synthetically accessible nucleophiles.

Both fluoroalkene and fluoroarene products have synthetic utility. Substituted fluoroarenes are applied in liquid crystal displays,^37,38^ light-emitting diodes,^39,40^ and as precursors for fluorinated synthons.^41,42^**1a** has also been highlighted as a protected form of phenol and can regenerate the phenol and pentafluoropyridine under mild conditions.^43^ Vinyl fluoride functional groups, such as those in **2a**-**2d**, are highly desirable due to their role as bioisosteres for amide and enol functional groups.^11,44−52^

### Kinetic Analysis

Kinetic analysis was used to gain insight into the new catalytic protocol. The experimentally determined empirical rate law for the reaction of 4-methoxyphenol (HX), pentafluoropyridine (fluoroarene), and excess diphenylacetylene with 10 mol % [Au(IPr)N^i^Pr_2_] (**cat**) is given in eq 1.
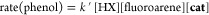
1

The reaction was found to be first order in HX, first order in fluoroarene and first order in the catalyst. Initial rates were used to determine the catalyst order, while pseudo-first-order conditions and timecourse data over 3 half-lives were used to determine the order in HX and fluoroarene.^53^ Due to the strict requirement to run the reaction in excess of alkyne (>2 equiv, outlined by previous studies^1^ and confirmed in optimization reactions), orders for this reagent were not determined.

**Figure 3:**
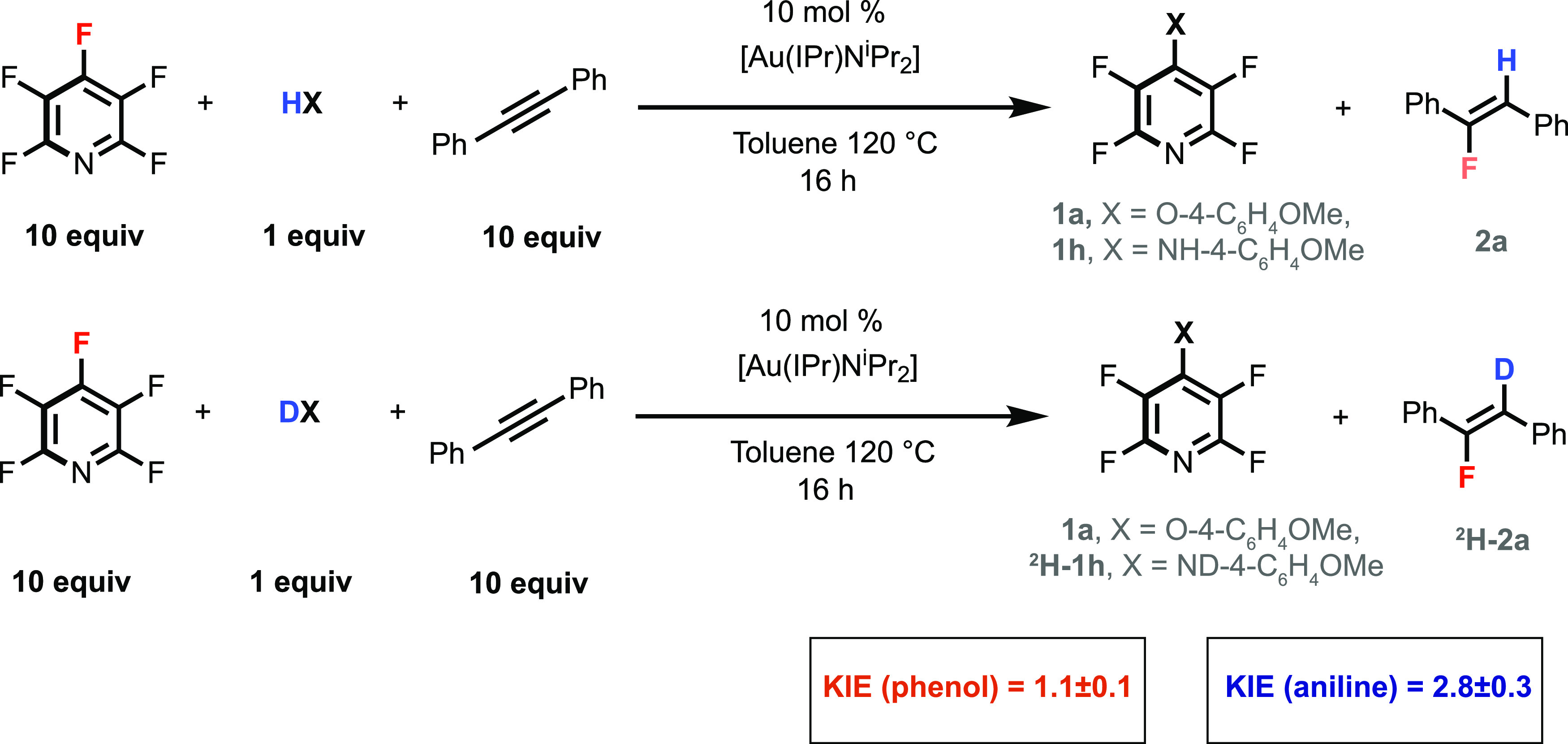
KIEs determined by independent rates.

Kinetic experiments were also run using 4-methoxyaniline as a nucleophile in place of 4-methoxyphenol. The interpretation of this data is complicated by the observation that **1h** and **2a** form at different rates for this nucleophile (*vide infra*). Despite this limitation, the analysis suggests the formation of **2a** is 0th order in fluoroarene and first order in HX and strongly indicates that the mechanism may change, depending on the nature of the nucleophile. This finding is further supported by the measurement of KIEs, which indicate a small isotope effect of 1.1 ± 0.1 for the reaction of 4-methoxyphenol/*d*_1_–4-methoxyphenol but a clear primary KIE of 2.8 ± 0.3 for the formation of **2a** from 4-methoxyaniline/*d*_2_–4-methoxyaniline. These KIE values are consistent across both independent rates measurements and intermolecular competition experiments (Figure 3).

### DFT Calculations

**Figure 4:**
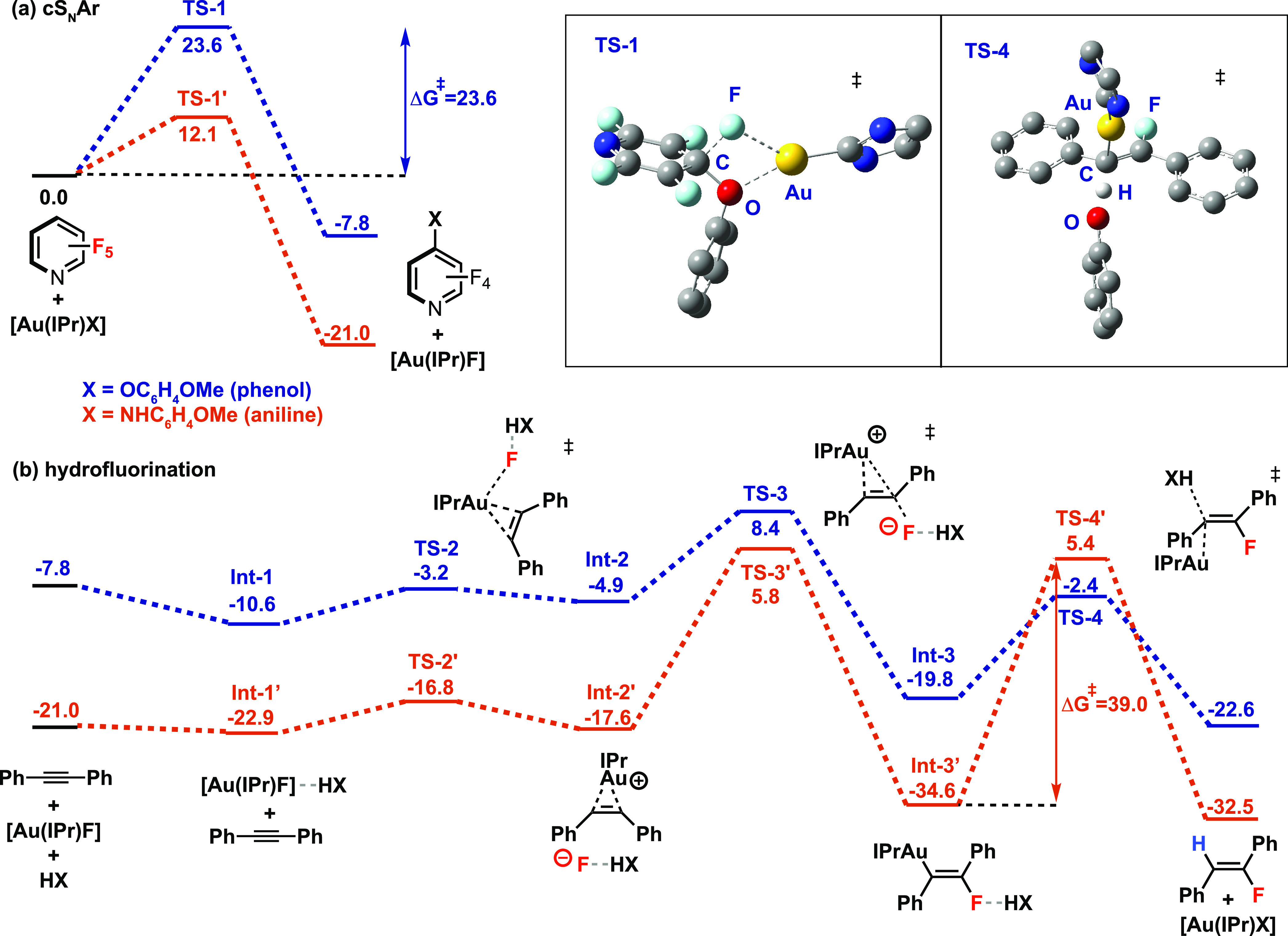
DFT calculated pathways for (a) cS_N_Ar and (b) hydrofluorination reaction pathways. (inset) Models of **TS-1** and **TS-4** showing geometries of key steps. Free energy values are calculated at 298.15 K.

DFT calculations were performed to supplement the kinetic data and used as a foundation with which to build a mechanistic model. The PBE0 functional, which has previously been used to model Au(I) interactions with alkynes,^54,55^ and 6–311G** basis set were used for all atoms other than Au, for which the SDDAll pseudopotential was applied. A single-point empirical dispersion correction (D3) with Becke–Johnson damping and solvent correction (PCM, ε = 2.38) was applied to the energies of all stationary points. Pathways were calculated for both the reaction of 4-methoxyphenol and 4-methoxyaniline with pentafluoropyridine and diphenylacetylene (Figure 4). Initially, a simple model constructed from a catalytic cycle without considering off-cycle species was considered. Toste, Bergman, and co-workers have demonstrated that [Au(IPr)N^i^Pr_2_] does not react with diphenylacetylene below 75 °C but that this species is capable of deprotonating weak acids (e.g., fluorene p*K*_a_ = 23 in THF).^31^ Precatalyst initiation by protonolysis with HX was considered to be facile, and [Au(IPr)N^i^Pr_2_] was assumed to react with 4-methoxyphenol (p*K*_a_ = 19)^56^ and 4-methoxyaniline (p*K*_a_ ∼ 30)^57^ to form [Au(IPr)X] (X = O-4-C_6_H_4_OMe, NH-4-C_6_H_4_OMe).

The reaction of [Au(IPr)X] (X = O-4-C_6_H_4_OMe) with pentafluoropyridine is calculated to occur by a concerted S_N_Ar mechanism by **TS-1** (Δ*G*^‡^ = +23.6 kcal mol^–1^) to form **1a** alongside [Au(IPr)F]. **TS-1** bears all features expected for a concerted S_N_Ar (cS_N_Ar) process with charge accumulation and pyramidalization occurring at the ipso-carbon of the electrophile. The {Au(IPr)}^+^ fragment interacts with both the alkoxide nucleophile and the fluoride leaving group in **TS-1**, and concerted bond breaking and formation was confirmed by IRC calculations, which show only a single TS connecting starting materials and products. Prior computational studies on Au-catalyzed hydrodefluorination of fluoroarenes have modeled redox pathways but consider trigonal planar three-coordinate Au(I) intermediates supported by *bis*(phosphine) ligands rather than two-coordinate linear geometries.^36^

The hydrofluorination sequence evolves from [Au(IPr)F]. Explicit solvation of this species with the nucleophile was considered, leading to a series of structures stabilized by F–H–X hydrogen bonding interactions. Upon addition of diphenylacetylene, fluoride dissociation from **Int-1** to form **Int-2** is calculated to be endergonic (Δ*G*°_rxn_ = +5.7 kcal mol^–1^) and occurs by a facile interchange mechanism by **TS-2** (Δ*G*^‡^ = +7.4 kcal mol^–1^). π-Complexation of the alkyne to the cationic Au(I) fragment in **Int-2** is supported by NBO calculations; second-order perturbation analysis reveals both σ-donation and π-backdonation components to the bonding (see Supporting Information). This bonding interaction renders the alkyne ligand of **Int-2** susceptible to nucleophilic attack by the fluoride ion. **TS-3** shows that slippage of the alkyne from an η^2^ toward an η^1^ bonding mode occurs during this reaction pathway as evidenced by the asymmetry of the Au–C bond distances (2.13 vs 2.44 Å).^58^ Fluorination by **TS-3** ultimately leads to the vinyl Au(I) species **Int-3** with complete stereochemical control. The energy span between **Int-1** and **TS-3** defines the barrier for the fluorination sequence (Δ*G*^‡^ = +19.0 kcal mol^–1^), which is lower than the barrier for the cS_N_Ar step. Finally, protodeauration of **Int-3** by HX occurs via **TS-4** (Δ*G*^‡^ = +17.4 kcal mol^–1^) and leads to the hydrofluorination product **2a** while regenerating the active catalyst [Au(IPr)X]. NBO analysis reveals partial C–H bond formation and C–Au bond breaking in the transition state, while IRC calculations are consistent with the association of the resulting charged fragments {Au(IPr)}^+^ and X^–^ occurring afterward.^59−64^ The overall transformation is calculated to be exergonic (Δ*G*°_rxn_ = −22.6 kcal mol^–1^), with the S_N_Ar step being turnover-limiting.

A closely related pathway was calculated for the reaction of [Au(IPr)X] (X = NH-4-C_6_H_4_OMe) with pentafluoropyridine and diphenylacetylene, albeit with significantly modified barriers for each of the steps. The improved nucleophilicity of the amide ligand of [Au(IPr)NH-4-C_6_H_4_OMe] (NPA charges Au = +0.42, N = −1.00) over the alkoxide of [Au(IPr)O-4-C_6_H_4_OMe] (NPA charges Au = +0.45, O = −0.82), alongside the reduced p*K*_a_ of HO-4-C_6_H_4_OMe compared to H_2_N-4-C_6_H_4_OMe impacts key transition state barriers. The S_N_Ar step by **TS-1′** is now a low energy process (Δ*G*^‡^ = +12.1 kcal mol^–1^) and, as such, is no longer predicted to be the turnover-limiting step. In contrast, the protodeauration step from the Au(I) vinyl intermediate **Int-3′** to **TS-4′** involves a large energy barrier (Δ*G*^‡^ = +40.0 kcal mol^–1^) and is not only predicted to be the slowest step of the catalytic sequence, but the activation energy is also large enough to question if it is accessible under the reaction conditions (120 °C, 16 h).

Comparison of the **TS-1** and **TS-1′** allows identification of the key features that led to the lowering of this barrier for the cS_N_Ar step. The C–X and C–F distances in **TS-1** are 1.44 (X = O) and 1.65 Å, respectively, while the X–C–F angle is 89.9°. In contrast, **TS-1′** possesses a more open structure with a much longer C–X interaction of 1.92 Å (X = N), shorter C–F distance of 1.36 Å, and obtuse X–C–F angle of 94.1°. These metrics suggest that C–F bond breaking in **TS-1′** is less advanced than in **TS-1**. The accumulation of charge on key moieties in these transition states is consistent with this argument as **TS-1** (Δ*q*: Au = +0.07, O = +0.20, C_ipso_ = +0.04, F = −0.23) involves greater charge separation than **TS-1′** (Δ*q*: Au = +0.03, N = +0.12, C_ipso_ = +0.03, F = −0.13).^65^ A competition experiment in which excess 4-methoxyphenol and 4-methoxyaniline were reacted with pentafluoropyridine and 10 mol % [Au(IPr)N^i^Pr_2_] led exclusively to **1h** in preference to **1a**. This finding reflects the large energy difference between **TS-1′** and **TS-1** (ΔΔ*G*^‡^ = 11.5 kcal mol^–1^).

Further comparisons can be made between the protodeauration transition states **TS-4** and **TS-4**′. Protodeauration involves direct breaking of the H–X bond (X = O, N) through deprotonation by the Au–C moiety.^59−63^ In **TS-4**, the C–H–X bond angle is 174.1° while the H–C–Au angle is 90.7°, and this reflects the orthogonality between the reacting ligand and Au center. The protodeauration transition states **TS-4** and **TS-4′** both involve isomerization of the vinyl ligand from a σ- to π-coordination mode. This reorganization leads to an elongation of the Au–C bond length (e.g., **Int-3**, 2.04; **TS-4**, 2.18) and charge accumulation on the C atom adjacent to Au (e.g., **Int-3**, −0.44; **TS-4**, −0.57), both of which facilitate protonation. The acidity of the H–X moiety is a key factor in determining the activation barrier for this step. The transition state geometries would be expected to be consistent with a primary KIE if this step becomes turnover limiting.

In combination, the empirical rate laws, the KIEs, and the DFT calculations are fully consistent with a change of turnover-limiting step depending on the nucleophile. For 4-methoxyphenol, the cS_N_Ar is expected to be turnover-limiting, with a small or no KIE. For 4-methoxyaniline, the protodeuaration step becomes turnover-limiting; the reaction rate no longer depends on [fluoroarene], and a primary KIE is expected. Given the apparent sensitivity of these key steps to the electronics of the nucleophile, further DFT calculations were undertaken in which the nucleophile was modified through variation of the substituent at the 4-position (see Supporting Information for details). These calculations revealed clear free energy relationships between the transition state barriers and Hammett parameters (σ_p_) of the nucleophile. In no case did varying the electronics of phenol or aniline change the predicted turnover-limiting step for each of these systems; the switch-in mechanism requires a complete switch in nucleophile rather than perturbation of its electronic structure.

### Catalytic Network

**Figure 5:**
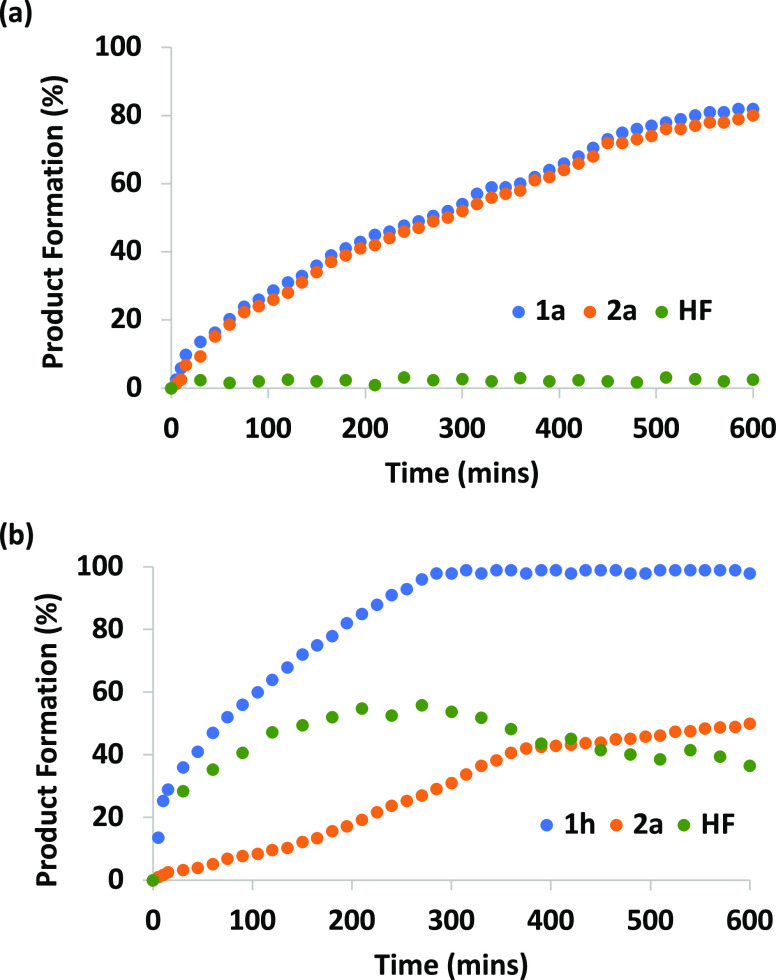
Plots for the concentration of fluoroarene and fluoroalkene products and HF intermediate over time for the reaction of pentafluoropyridine and diphenylacetylene with (a) 4-methoxyphenol and (b) 4-methoxyaniline.

Taken in combination, these data suggest a complex catalytic cycle involving both cS_N_Ar and hydrofluorination mechanisms within a single reaction network. Further insight into the different behavior of the two nucleophile types was obtained by following the concentration of key species (including HF) over the complete timecourse of these reactions (Figure 5). In the case of pentafluoropyridine, 4-methoxyphenol, and diphenylacetylene, the formation of products **1a** and **2a** was concurrent, and no HF buildup was observed. For the same reaction with 4-methoxyaniline, the formation of product **1h** occurred independently of the formation of **2a**. HF was identified as a reaction intermediate by a broad and concentration-dependent resonance in the ^19^F NMR spectrum at δ = 150.5–152.5 ppm. Given the reaction conditions, it remains likely that the HF interacts with 4-methoxyaniline in solution (through proton transfer or hydrogen bonding).^66^ An induction period was observed for the formation of **2a**, and an increase in the rate of formation of this species is observed as the HF concentration increases.

**Figure 6:**
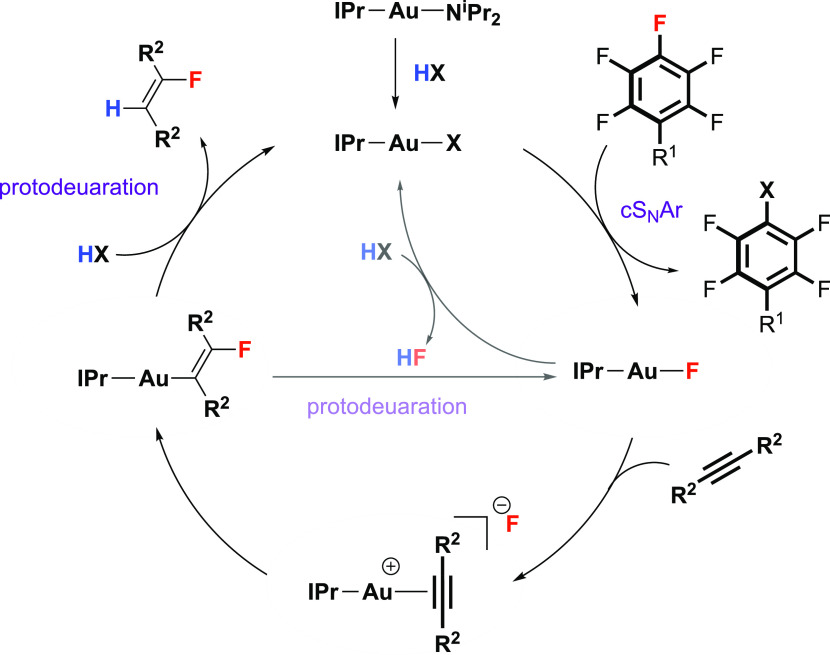
Plausible catalytic network for HF transfer.

The catalytic network in Figure 6 is a plausible reaction mechanism. This network explains the combined data and calculations. The reaction potentially operates in two different regimes depending on the nature of the nucleophile. In regime 1, for phenol-based nucleophiles, the cS_N_Ar step is expected to be slow and turnover-limiting. As such, any [Au(IPr)F] generated is expected to be consumed in the onward hydrofluorination sequence. While [Au(IPr)F] can also potentially react with HX to liberate HF and regenerate the active catalyst, this reaction is calculated to be endergonic (X = O-4-C_6_H_4_OMe; Δ*G*° = 9.6 kcal mol^–1^, Δ*G*^‡^ = 12.0 kcal mol^–1^) and should be reversible under the catalytic conditions. Protodeauration with HX is facile, leading to the synchronous formation of the products **1** and **2** and no buildup of HF during the reaction.

In regime 2, for aniline-based nucleophiles, the cS_N_Ar step is now fast, and the hydrofluorination sequence is slow and likely turnover limiting. The extremely high calculated activation barrier for protodeuaration with HX (Δ*G*^‡^ = 40.0 kcal mol^–1^) suggests that this step may only be a minor contributor under the reaction conditions. Instead, the reaction of [Au(IPr)F] with HX could produce a bypass in the catalytic cycle, resulting in the generation of HF. While again calculated to be endergonic and reversible (X = NH-4-C_6_H_4_OMe; Δ*G*° = 10.5 kcal mol^–1^, Δ*G*^‡^ = 11.3 kcal mol^–1^), if this bypass step operated in combination with the consumption of HF in the protodeuaration step, it may prove thermodynamically viable. If combined with the fast cS_N_Ar step, this bypass mechanism would lead to the asynchronous production of **1** and **2** along with the potential buildup of HF as a reaction intermediate. Control reactions revealed that under catalytic conditions, **1h** forms in near-quantitative yield, alongside HF, even if diphenylacetylene was omitted from the reaction mixture. As HF is susceptible to off-cycle side reactions, this mechanism would explain the lower yield of **2** for aniline-based nucleophiles compared to phenol-based nucleophiles (Figure 2).

This mechanistic hypothesis could be used to improve the yields of the HF transfer product **2** when carrying out reactions with aniline-based nucleophiles. The catalytic reaction to form **1h** in 83 ± 5% yield generates 50 ± 2% of **2a** as a coproduct (Figure 2). When the standard conditions are repeated but 1.2 equiv of 4-methoxyphenol is added to the reaction mixture, **1h** is still formed as the exclusive cS_N_Ar product in 92 ± 5%, but the yield of **2a** improves to 72 ± 3%. The finding is consistent with the addition of the phenol-limiting bypass catalysis by accelerating the protodeauration step.

## Conclusions

In summary, a Au(I)-catalyzed HF transfer reaction for the tandem hydrofluorination of alkynes and functionalization of fluoroarenes has been developed. HF is generated through the reaction of perfluoroarene with a nucleophile, obviating the need for direct handling of HF-based reagents and providing an operationally simple approach to fluorination catalysis. Through kinetics analysis, competition experiments, and DFT calculations, a detailed understanding of the catalytic network involved in HF transfer has been obtained. These studies showed that the rate of production and distribution of products is dependent on the nature of the nucleophile. This mechanistic understanding was exploited to improve the efficiency of HF transfer catalysis. In the longer term, we believe that these results will provide a foundation for the development of new catalytic approaches to transfer fluorine-containing groups between molecules and recycle fluorinated compounds.

## Supporting Information Available

Full experimental details: synthetic procedures, computational methods, and analysis; primary data (.mnova,.xyz) are available through the following link: https://data.hpc.imperial.ac.uk/resolve/?doi=9985 (PDF)
