# Supplementary material for: Au(I) Catalyzed HF Transfer: Tandem Alkyne Hydrofluorination and Perfluoroarene Functionalization
Source: ACS Catal. 2022 Mar 1;12(6):3411–9. doi: 10.1021/acscatal.1c05474 (PMC9007466; doi:10.1021/acscatal.1c05474)
Supplement: Supplementary file 1 — cs1c05474_si_001.pdf [file cs1c05474_si_001.pdf]

Supporting Information for:

Au(I) Catalyzed HF Transfer: Tandem Alkyne Hydrofluorination and Perfluoroarene Functionalisation

Daniel Mulryan, Jack Rodwell, Nicholas Phillips, and Mark R. Crimmin*

d.mulryan18@imperial.ac.uk, *m.crimmin@imperial.ac.uk

Molecular Sciences Research Hub, Imperial College London, 82 Wood Lane, Shepherds Bush, W12 0BZ, UK

Contents:

**1) Experimental Methods**	**S2**
**1.1) Starting Material Synthesis and Characterisation**	**S3**
**2) Optimisation of Reaction Conditions**	**S10**
**2.1) Catalyst Screening**	**S11**
**2.2) Conditions Screening**	**S12**
**2.3) Equivalents of Reagents Screening**	**S13**
**3.1) General Reaction Procedure**	**S14**
**3.2) Product Characterisation**	**S15**
**3.3) Substrate Competition Reactions**	**S42**
**4) DFT Studies**	**S43**
**4.1) Calculated Reaction Pathway with 4-Methoxyphenol Mechanism**	**S44**
**4.2) 4-Methoxyphenol Mechanism Functional Testing**	**S44**
**4.3) Intermediates and Transition States for the 4-Methoxyphenol Mechanism**	**S45**
**4.4) Calculated Reaction Pathway with 4-Methoxyaniline Mechanism**	**S47**
**4.5) 4-Methoxyaniline Mechanism Functional Testing**	**S47**
**4.6) Geometries of Intermediates and Transition States for the 4-Methoxyaniline Mechanism**	**S48**
**4.7) Uncatalyzed S_NAr Energy Barriers**	**S50**
**4.8) Hammett Plots and Energy Profiles for Para-Substitution Variation**	**S51**
**5) Kinetic Studies**	**S53**
**5.1) Catalyst Order for 4-Methoxyphenol Reaction**	**S54**
**5.2) Catalyst Order for 4-Methoxyaniline Reaction**	**S55**
**5.3) Pentafluoropyridine Order for 4-Methoxyphenol Reaction**	**S56**
**5.4) Paramethoxyphenol Order for 4-Methoxyphenol Reaction**	**S57**
**5.5) Pentafluoropyridine Order for 4-Methoxyaniline Reaction**	**S58**
**5.6) Paramethoxyaniline Order for 4-Methoxyaniline Reaction**	**S59**
**5.7) Product Formation Plots**	**S60**
**5.8) Kinetic Isotope Effects: Competition Experiments**	**S61**
**5.9) Kinetic Isotope Effects: Rates Experiments**	**S62**
**6) References**	**S64**
**7) XYZ Coordinates**	**S66**

1. Experimental Methods

All manipulations were carried out using standard Schlenk-line and glovebox techniques under an inert atmosphere of dinitrogen. An MBraun Labmaster glovebox was employed, operating at <0.1 ppm O_2 and <0.1 ppm H_2O. Solvents were dried over alumina from an SPS (solvent purification system) and degassed before use. Glassware was dried for 12 h at 120 °C prior to use. Benzene-d_6 was stored over 3 Å molecular sieves and distilled prior to use. NMR scale reactions were conducted in J. Young's tap tubes and prepared in a glovebox. Scaled-up reactions were conducted in a PTFE lined 30 ml reaction vessel, containing a PTFE-coated magnetic stirrer bar and sealed with electrical tape. Heating of NMR scale reactions was done using silicone oil baths and heating of scaled-up reactions was done using a sand bath. ^{1}H, ^{13}C, and ^{19}F NMR spectra were obtained on BRUKER 400 MHz or 500 MHz machines, unless otherwise stated, and referenced against $SiMe_4$ (^{1}H, ^{13}C) or $CFCl_3$ (^{19}F). All peak intensities are derived against an internal standard peak (^{1}H m-xylene; δ_H = 2.30 ppm, ^{19}F fluorobenzene; δ_F = -110.7 ppm). NMR data was processed using MestReNova software. Multiplicity assignments for NMR spectra are labelled as follows: "s" = singlet, "d" = doublet, "t" = triplet, "q" = quartet, "hept" = heptet, "m" = multiplet. Some ipso- and fluorocarbon environments on arene rings are not observed by ^{13}C NMR and this is indicated in the product characterisation in each case.

Phenols and anilines were sublimed or distilled prior to use. $LiN^{i}Pr_2$ was sublimed prior to use. Fluoroarenes and fluoroalkanes were dried over activated 3 Å molecular sieves and freeze-pump-thaw degassed before use. 1,2-bis(4-fluorophenyl)ethylene and 1,2-bis(4-methoxyphenol)ethylene were synthesised according to the literature report by Grieco and co-workers.[S1] (Cyclohexylethynyl)benzene was synthesised according to the literature report by Tsuhi and co-workers.[S2] All other chemicals were purchased from Sigma Aldrich and used without purification unless stated.

1.1. Synthesis of [Au(^{t}BuXantphos)Cl]

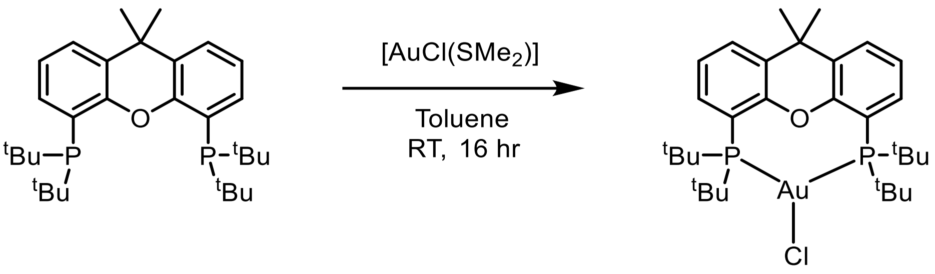

1

According to the literature procedure reported by Zhang and co-workers, the gold catalyst **1** was synthesised.[S3]

In a glovebox under an inert atmosphere, ^{t}BuXantPhos (68.2 mg, 0.14 mmol) was dissolved in toluene (3 ml) and [AuCl(SMe$_2$)] (40.3 mg, 0.14 mmol) was added. This reaction mixture was then stirred for 16 hours at room temperature in darkness and filtered through a celite plug. The solvent was then removed in vacuo to afford catalyst **1** as an off-white solid (85.0 mg, 0.12 mmol, 85 %).

^{1}H NMR (400 MHz, Benzene-d$_6$): δ 7.44 (m, 2H), 7.10 (m, 2H), 6.81 (m, 2H), 1.48 (d, $^3J_{P\text{-}H}$ = 7.7 Hz, 18H), 1.46 (d, $^3J_{P\text{-}H}$ = 7.7 Hz, 18H), 1.23 (s, 6H).

^{31}P NMR (200 MHz, Benzene-d$_6$): δ 52.7 (s, 2P).

1.2. Synthesis of [Au(IPr)N^{i}Pr$_2$]

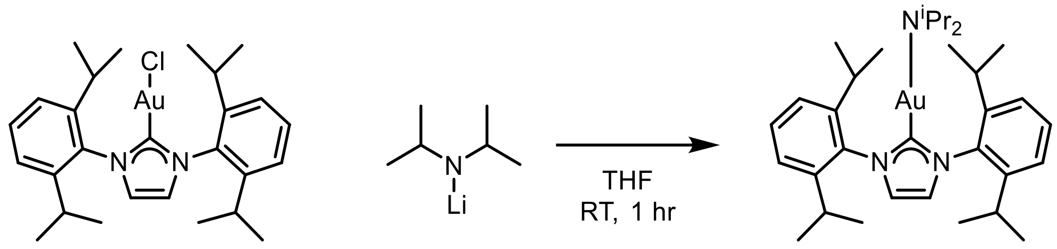

2 **3**

According to the literature procedure reported by Toste and co-workers, the gold catalyst **3** was synthesised.[S4]

In a glovebox in the dark, [Au(IPr)Cl], **2,** (500 mg, 0.8 mmol) was dissolved in THF (30 ml). Lithium diisopropylamine (100 mg, 0.9 mmol,) was added and the reaction was stirred in darkness for one hour at room temperature. The reaction mixture was then dried in vacuo to afford crude **3** as an off-yellow solid. This was then dissolved in n-hexane and filtered, resulting in a luminous green solution which was dried in vacuo to afford of **3** as a colourless solid (400 mg, 0.64 mmol, 80 %).

^{1}H NMR (400 MHz, Benzene-d$_6$): δ 7.32-7.21 (m, 2H), 7.12-7.09 (m, 4H), 6.34 (s, 2H), 3.83 (hept, $^3J_{H-H}$ = 6.1 Hz, 2H), 2.72 (hept, $^3J_{H-H}$ = 7.0 Hz, 4H), 1.53 (d, $^3J_{H-H}$ = 6.8 Hz, 12H), 1.08 (d, $^3J_{H-H}$ = 7.0 Hz, 24H).

1.3. Synthesis of [Au(IPr)O^{t}Bu]

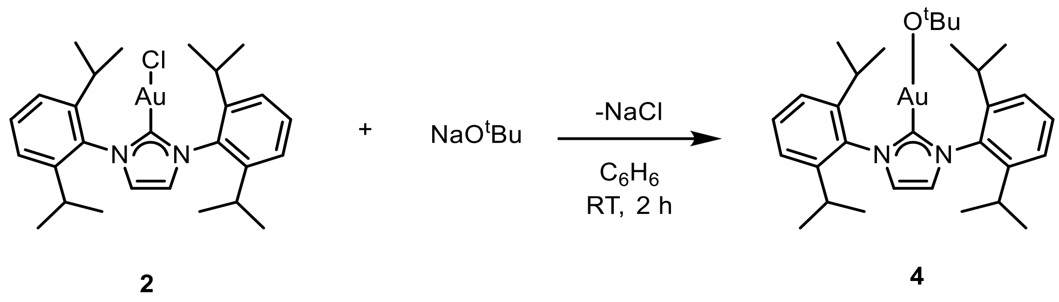

According to the literature reported by Sadighi and co-workers, [Au(IPr)O^{t}Bu] was synthesised.[S5]

In a glovebox, under an inert atmosphere, [Au(IPr)Cl] (104 mg, 0.17 mmol) was dissolved in benzene (3 mL) and NaO^{t}Bu (16 mg, 0.17 mmol) was added. The reaction mixture was stirred for 2 hours in darkness and then filtered through celite to afford a clear solution. The solvent was then removed in vacuo to afford [Au(IPr)O^{t}Bu] (45mg, mmol, 43%) as an off-white solid.

^{1}H (400 MHz, THF-d_8): δ 7.39 – 7.21 (m, 6H), 6.25, (s, 2H), 2.53 (hept, $^3J_{H\text{-}H}$ = 6.8 Hz, 4H), 1.24 (d, $^3J_{H\text{-}H}$ = 6.9 Hz, 12H), 1.05 (d, $^3J_{H\text{-}H}$ = 6.9 Hz, 12H), 0.63 (s, 9H).

1.4 Synthesis of [Au(SIPr)Cl]

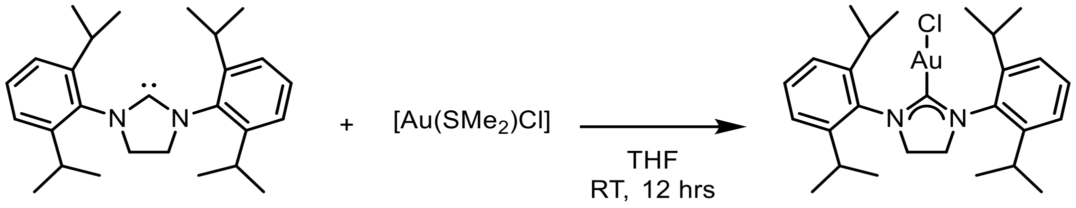

5

According to the literature report by Nolan and co-workers, [Au(SIPr)Cl] was synthesised.[S6]

In a glovebox under an inert atmosphere, 1,3-bis(2,6-di-isopropylphenyl)imidazolidin-2-ylidene (125 mg, 0.32 mmol) was dissolved in THF (10 ml) and added to [Au(SMe_2)Cl] (100 mg, 0.34 mmol) in a scintillation vial. The reaction mixture was stirred at room temperature in darkness for 12 hours and then filtered through celite. The reaction was quenched using activated carbon, stirred for 4 hours and then filtered through celite again. The filtrate was evaporated *in vacuo* and the remaining solid was recrystallised from DCM (4 ml) using pentane (12 ml). The addition of pentane resulted in the precipitation of the product, which was collected by filtration and then washed with pentane and dried *in vacuo* to afford [Au(SIPr)Cl] (140 mg, 0.22 mmol, 70%).

^{1}H NMR (400 MHz, Benzene-d_6): δ 7.04 – 6.91 (m, 6H), 3.10 (s, 4H), 2.92 (hept, $^3J_{H\text{-}H}$ = 7.0 Hz, 4H), 1.46 (d, $^3J_{H\text{-}H}$ = 6.8 Hz, 12H), 1.14 (d, $^3J_{H\text{-}H}$ = 6.9 Hz, 12H).

1.4 Synthesis of 1,2-bis(4-methoxyphenol)ethylene

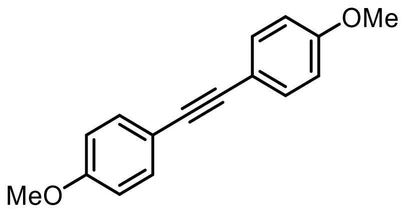

According to the literature report by Grieco and co-workers, 1,2-bis(4-methoxyphenol)ethylene was synthesised.[S1]

Under an inert atmosphere, an 8 ml ampoule containing a magnetic stirrer bar was charged with Bis(triphenylphosphine)palladium(II) dichloride (27.4 mg, 0.04 mmol), copper Iodide (24.8 mg, 0.13 mmol), and 4-bromoanisole (163 µl, 1.30 mmol). 6.5 ml of dry n-hexane and 1,8-Diazabicyclo[5.4.0]undec-7-ene (1.17 ml, 8.00 mmol) were added. The reaction was cooled to 0 °C with an ice-water bath before trimethylsilylacetylene (92 µl, 0.65 mmol) was added. The reaction mixture was then heated to 60 °C and stirred for 18 hours in the dark. After 18 hours, reaction mixture was decanted from the ampoule, and washed with diethyl ether and water (50 ml each). The combined organic layer was washed with 10 % HCl (3×75 ml) and saturated NaCl (75 ml), before being dried over magnesium sulfate. The crude product was filtered, concentrated and columned in a mixture of 4:1 n-pentane:DCM (R_F = 0.3)

^{1}H NMR (100 MHz, $CDCl_3$): δ 7.96 (d, $^{3}J_{H\text{-}H}$ = 8.3 Hz, 4H), 7.63 (d, $^{3}J_{H\text{-}H}$ = 8.1 Hz, 4H), 2.62 (s, 6H).

1.5 Synthesis of 1,2-bis(4-fluorophenyl)ethylene

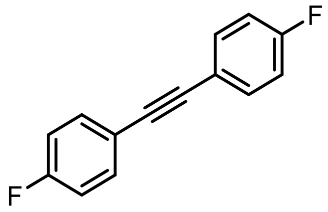

According to the literature report by Grieco and co-workers, 1,2-bis(4-fluorophenol)ethylene was synthesised.[S1]

In an inert atmosphere, an 8 ml ampoule containing a magnetic stirrer bar was charged with Bis(triphenylphosphine)palladium(II) dichloride (27.4 mg, 0.04 mmol), copper Iodide (24.8 mg, 0.13 mmol), and 4-fluoroiodobenzene (149 µl, 1.3 mmol). 6.5 ml of dry hexane and 1,8-diazabicyclo[5.4.0]undec-7-ene (1.17 ml, 8.00 mmol) were added. The reaction was cooled to 0 °C with an ice-water bath before trimethylsilylacetylene (92 µl, 0.65 mmol) was added. The reaction mixture was then heated to 60 °C and stirred for 18 hours in the dark. After 18 hours, the ampoule was unsealed and the reaction mixture was washed with ethyl ether and water (50 ml each). The organic layer was washed with 10 % HCl (3×75 ml) and saturated NaCl (75 ml), before being dried over magnesium sulfate. The crude product was filtered, concentrated and columned in n-hexane (R_F = 0.14).

^{1}H NMR (100 MHz, $CDCl_3$): δ 7.50 (m, 4H), 7.05 (t, $^3J_{H-H}$ = 8.6, 4H).

^{19}F NMR (100 MHz, $CDCl_3$): δ -112.4 (m, 2F)

1.6 Synthesis of (cyclohexylethynyl)benzene.

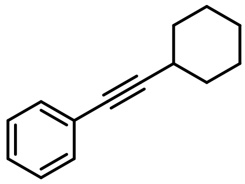

According to the literature reported by Tsuji and co-workers, (cyclohexylethynyl)benzene was synthesised. [S2]

A 50 mL Schlenk tube was charged under nitrogen with 106 mg of $PdCl_2(PPh_3)_2$ (0.15 mmol, 0.02 equiv.), 58.3 mg CuI (0.30 mmol, 0.04 equiv.), and 25 mL HN^iPr_2. 850 µL of iodobenzene (7.6 mmol, 1 equiv.) and 1 g of cyclohexylacetylene (9.2 mmol, 1.2 equiv.) were added slowly to the reaction mixture. The solution was stirred for 16 h at room temperature. After the reaction, 40 mL of diethyl ether was added to the reaction mixture. The organic phase was washed with water and saturated NaCl solution (30 mL, each), dried with magnesium sulfate, and filtered. The solvent was removed under reduced pressure and the crude product was purified by column chromatography in n-pentane (R_F = 0.7).

^{1}H NMR (400 MHz, C_6D_6): δ 7.52-7.43 (m, 2H, C_{Ph}H), 7.03-6.94 (m, 3H, C_{Ph}H), 2.53-2.45 (m, 1H, $C_{cyclohexyl}$H), 1.85-1.72 (m, 2H, $C_{cyclohexyl}$H), 1.68-1.44 (m, 4H, $C_{cyclohexyl}$H), 1.24-1.06 (m, 4H, $C_{cyclohexyl}$H).

^{13}C NMR (100 MHz, C_6D_6): δ 131.6 (C_{Ph}H), 128.2 (C_{Ph}H), 127.3 (C_{Ph}H), 124.6 ($\underline{C}_{Ph}$-C_{alkyne}), 94.3 ($C_{Cyclohexyl}$-$\underline{C}_{alkyne}$), 81.2 (C_{Ph}-C_{alkyl}), 32.7 ($C_{cyclohexyl}$H), 29.7 ($C_{cyclohexyl}C_{alkyne}$), 25.8 ($C_{cyclohexyl}$H), 24.7 ($C_{cyclohexyl}$H).

2. Optimisation of Reaction Conditions.

Initial optimisation of the reaction conditions were conducted with 0.1:1:1:3 equiv. of catalyst : pentafluoropyridine : 4-methoxyphenol : diphenylacetylene. Reactions were run in PTFE tubes. 4-Methoxyphenol was used as the nucleophile of choice due to higher conversion to product **2a** than with phenol in a preliminary screening. Three equivalents of diphenylacetylene were added to maintain an excess of diphenylacetylene throughout the reaction, as prior studies had demonstrated that an excess of alkyne is necessary to achieve the fluoroauration of the alkyne and that addition of [Au(IPr)F] to diphenylacetylene is reversible.[S6]

2.1 Catalyst Screening

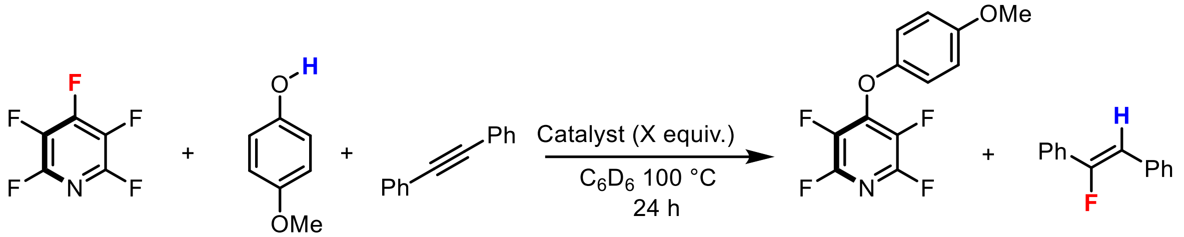

1 equiv. **[0.04] M** | **1 equiv.** | **3 equiv.** | **1a** | **2a**

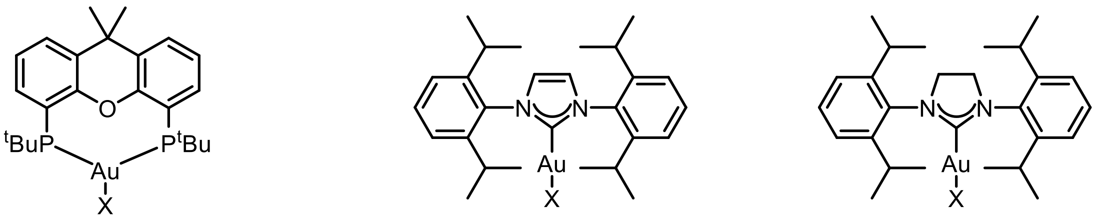

[Au(^{t}BuXantPhos)X] | **[Au(IPr)X]** | **[Au(SIPr)X]**

Entry	Catalyst	Equivalents of Catalyst	1a (%)	2a (%)
1	[Au(^{t}BuXantPhos)Cl] + AgF	0.1	0	0
2	[Au(IPr)Cl] + AgF	0.1	0	0
3	[Au(SIPr)Cl] + AgF	0.1	0	0
4	[Au(IPr)O^{t}Bu]	0.1	43	17
5	[Au(IPr)N^{i}Pr$_2$]	0.01	26	Trace
6	[Au(IPr)N^{i}Pr$_2$]	0.05	28	6
7	[Au(IPr)N^{i}Pr$_2$]	0.1	47	13
8	None	0	0	0

Table S1. Percentage production of products **1a** and **2a** with a variety of homogeneous Au(I) catalysts. Reactions run with 1:1:3 equivalents of pentafluoropyridine (0.036 mmol, 0.04 M) : paramethoxyphenol : diphenylacetylene in 1.0 ml C_6D_6. Reactions were run in PTFE inserts inside J-Young's NMR tubes. Production of **1a** and **2a** was monitored by ^{19}F NMR spectroscopy using fluorobenzene as an internal standard.

Multiple side-products were observed in the ^{19}F NMR spectrum of Entry 4 which were attributed to 4-hydroxy-2,3,5,6-tetrafluoropyridine (δ = -92.8 and -164.2 ppm at 6 % conversion), and 4- (tert-butoxy)-2,3,5,6-tetrafluoropyridine (δ = --91.2 and -152.9 ppm at 8 % conversion). Owing to this unwanted side reactivity, further optimisations were conducted with [Au(IPr)N^{i}Pr$_2$], which did not display these side-products.

2.2 Conditions Screening

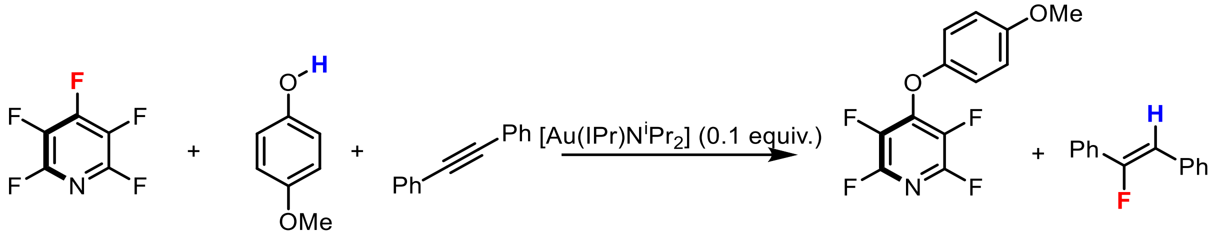

Entry	Solvent	Temperature (°C)	Reaction Time (h)	1a (%)	2a (%)
1	CH_2Cl_2	40	24	50	0
2	THF	60	24	58	0
3	CH_3CN	80	24	63	0
4	DMSO	120	24	99	0
5[a]	C_6D_6	100	24	35	7
6	C_6D_6	100	24	47	13
7	Toluene	100	24	4	15
8	Toluene	120	16	53	23

Table S2. Solvent, temperature and reaction time screening. Reactions run with 0.1:1:1:3 equivalents of [Au(IPr)N^{i}Pr$_2$] : pentafluoropyridine (0.036 mmol, 0.04 M) : 4-methoxyphenol : diphenylacetylene in 1.0 ml of solvent. Reactions were run in PTFE inserts inside J-Young's NMR tubes.[a] Entry 5 was run without a PTFE insert. Production of **1a** and **2a** was monitored by ^{19}F NMR spectroscopy using fluorobenzene as an internal standard.

2.3 Equivalents of Reagent Screening

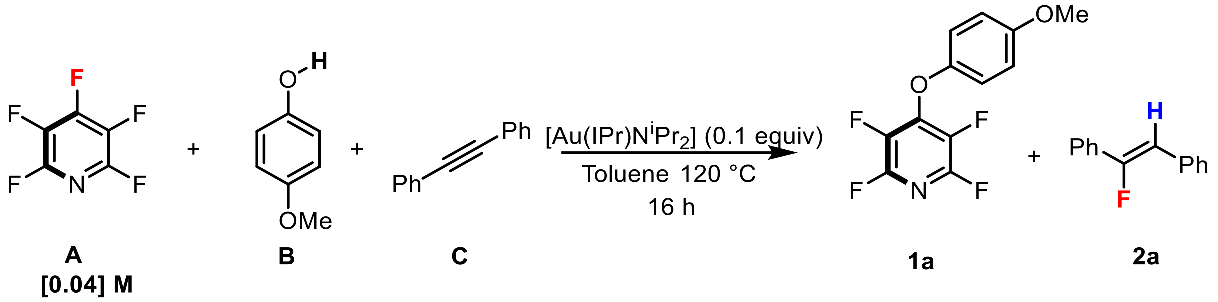

Entry	Ratio of equiv. A : B : C	**1a** (%)	**2a** (%)
1	1 : 1 : 1	53	23
2	1 : 1 : 2	58	38
3	1 : 1 : 3	63	45
4	1 : 1.1 : 3	78	72
5	1 : 1.2 : 3	83	80
6	1 : 1.2 : 1	56	49
7	1 : 1.2 : 2	84	79
8	1 : 1.5 : 2	86	82
9	1 : 2 : 2	94	81

Table S3. Reagent equivalents screening. Reactions run with 0.1:1 equivalents of [Au(IPr)N^{i}Pr$_2$] : pentafluoropyridine (0.036 mmol, 0.04 M) in 1.0 ml toluene. Reactions were run in PTFE inserts inside J-Young's NMR tubes. Production of products **1a** and **2a** was monitored by ^{19}F NMR spectroscopy using fluorobenzene as an internal standard.

3.1. General Reaction Procedure

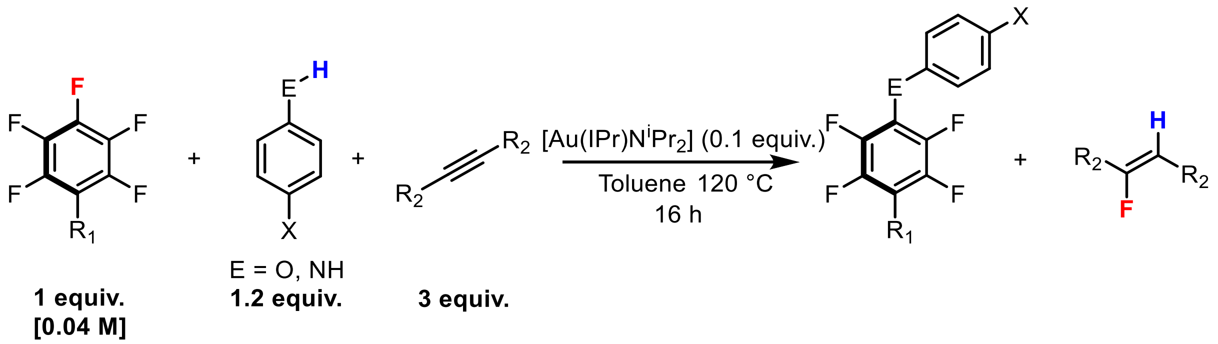

NMR Scale Procedure: In a glovebox, [Au(IPr)N^{i}Pr$_2$] (2.5 mg, 10 mol %) was dissolved in 0.1 ml C_6D_6 to give a yellow solution. A solution of nucleophile in 0.2 ml toluene (0.216 M, 0.043 mmol) was added which resulted in the rapid decolourisation of the solution. Then a solution of alkyne in 0.2 ml toluene (0.54 M, 0.108 mmol), followed by a 1:1 solution of fluoroarene : fluorobenzene (internal standard) in 0.6 ml toluene (0.06 M, 0.036 mmol) was added. The solution was transferred to a PTFE (or FEP) tube which was placed inside a J-Young's NMR tube and sealed. The reaction was heated for 16 hours at 120 °C in a silicone oil bath, ensuring that the tube is immersed in the heating medium up to the solvent line. Quantitative ^{19}F NMR spectroscopy integration was performed using the fluorobenzene singlet resonance at δ_F -112.7 ppm as an internal standard.

Preparative Scale: In a glovebox, [Au(IPr)N^{i}Pr$_2$] (25 mg, 10 mol %), alkyne (1.08 mmol), nucleophile (0.43 mmol) and fluoroarene (0.36 mmol) were added to a PTFE lined 15 ml vial with a PTFE covered stirrer bar. 10 ml of toluene was added, and the solution was stirred until homogeneous. The vial was sealed and heated in a sand bath for 16 h at 120 °C. After the 16 hours, the vial was removed and allowed to cool. Once cooled, the vial was opened, and the reaction solution was transferred to a Schlenk and the toluene solvent was removed under reduced pressure. The resulting solid was purified by column chromatography on silica gel (tech grades, 60 Å, 230-400 mesh, 40-63 µm particle size, 200 : 1 silica gel : crude mixture loading) to provide the isolated products. The eluent system for each separation is listed in the product characterisation below.

In some cases, separation of products from starting materials was not possible, or resulted in low isolated yields due to the discarding of mixed fractions.

3.2 Product Characterisation

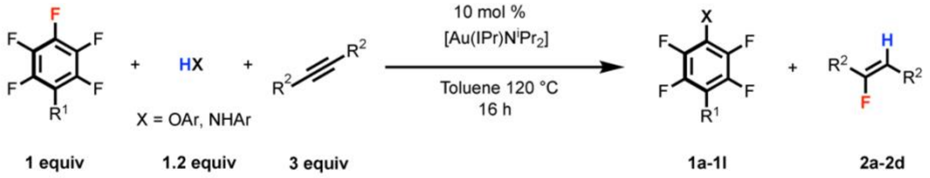

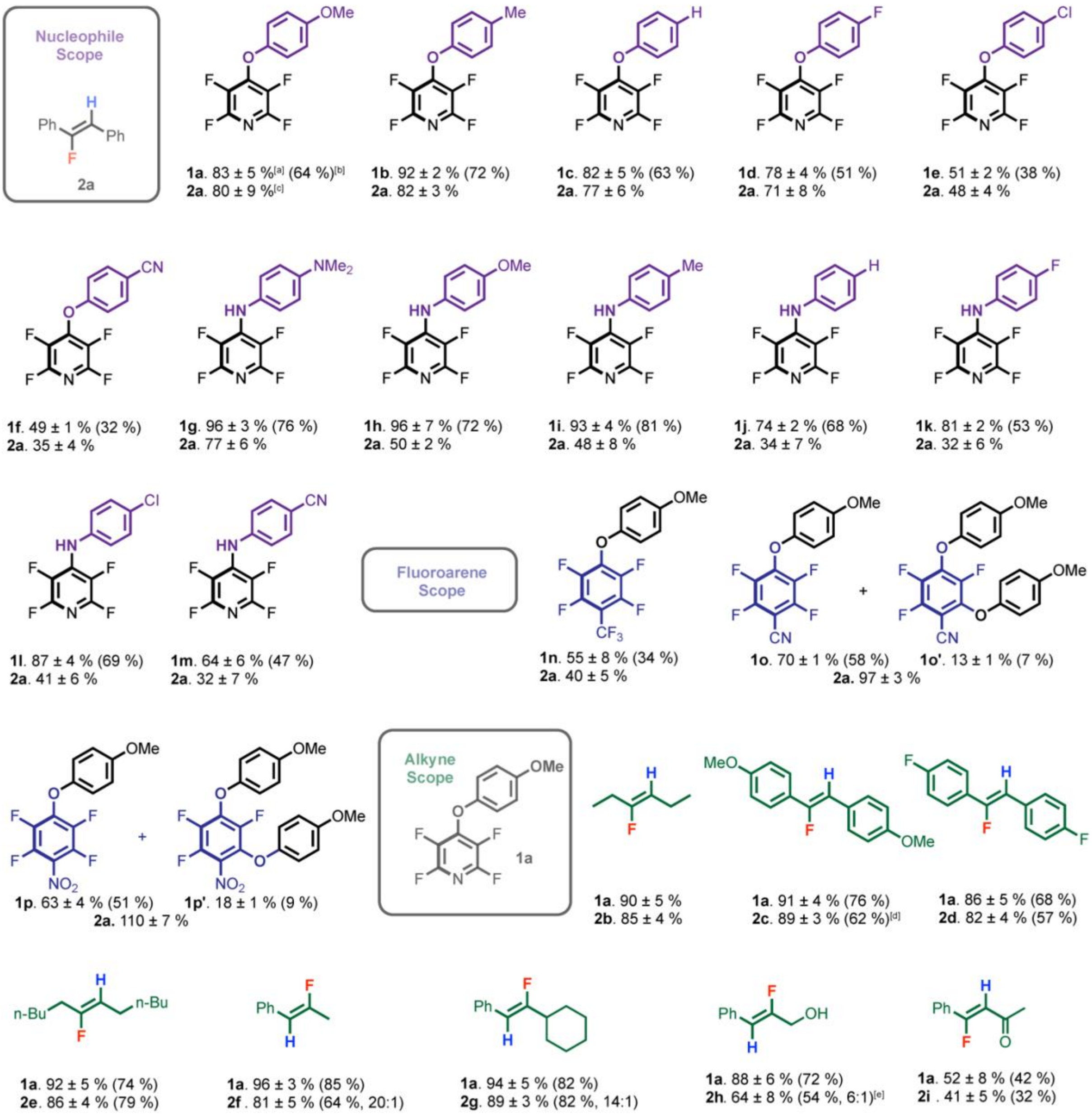

Figure S1. HF transfer reaction scope catalysed by [Au(IPr)N^{i}Pr$_2$]. [a] Reactions were performed with 0.1 : 1 : 1.2 : 3 equivalents of catalyst : fluoroarene (0.04 M) : nucleophile : alkyne. Yields of fluoroarene (**1a**-**1p**) and fluoroalkene (**2a**-**2d**) were calculated from ^{19}F NMR spectroscopy, using a fluorobenzene internal standard. Reactions were performed in triplicate and standard deviations are reported with a 99% confidence level. [b] Isolated yields were obtained from scale-up reactions and are shown in parenthesis. [c] Isolated yields of **2a** are not reported due to this compound co-eluting with diphenylacetylene. [d] Due to the challenging isolation, this product was contaminated with ~ 20% of unreacted alkyne. [e] Ratio of regioisomers β:α functionalisation. Major isomer shown.

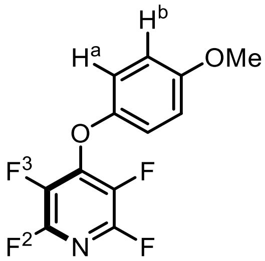

1a. 2,3,5,6-tetrafluoro-4-(4-methoxyphenoxy)pyridine Isolation achieved by column chromatography in 19:1 n-pentane : dichloromethane, R_F = 0.60. Isolated as a colourless solid. (62.8 mg, 0.23 mmol, 64 %).[S7]

^{1}H NMR (C_6D_6, 400 MHz): δ 6.61-6.50 (m, 4H, C_{Ph}), 3.21 (s, 3H, OCH_3).

^{13}C{^{1}H} NMR (C_6D_6, 100 MHz): δ 156.7 (s, $\underline{C}_{Ph}OCH_3$), 149.7 (s, $\underline{C}_{Ph}$-OC_{Pyr}), 143.8 (dm, $^{1}J_{C-F}$ = 246 Hz, CF^2), 135.5 (dm, $^{1}J_{C-F}$ = 257 Hz, CF^3), 131.6 (s, $C_{pyr}O$), 117.9 (s, CH^a), 114.7 (s, CH^b), 54.7 (s, OCH_3).

^{19}F (C_6D_6, 376.5 MHz): δ -89.7 (m, 2F, CF^2), -155.3 (m, 2F, CF^3).

HRMS (APCL): m/z: M^+ Calcd for $[C_{12}H_7F_4NO_2]^+$ 274.0862, observed 274.0862.

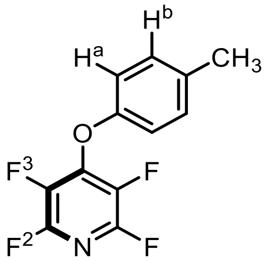

1b. 2,3,5,6-tetrafluoro-4-(p-tolyloxy)pyridine . Isolation achieved by column chromatography in 19:1 pentane : dichloromethane, R_F = 0.70. Isolated as a colourless solid. (66.7 mg, 0.26 mmol, 72 %).[S7]

^{1}H NMR (C_6D_6, 400 MHz): δ 6.74 (d, $^3J_{H\text{–}H}$ = 8.6 Hz, 2H, CH^b), 6.55 (d, $^3J_{H\text{–}H}$ = 8.6 Hz, 2H, CH^a), 1.98 (s, 3H, Me).

^{13}C{^{1}H} NMR (C_6D_6, 100 MHz): δ 153.8 (s, O-C_{ph}), 144.0 (dm, $^1J_{C\text{–}F}$ = 237 Hz, CF^2), 136.0 (dm, $^1J_{C\text{–}F}$ = 249 Hz, CF^3), 134.4 (s, C-CH_3), 130.2 (s, m-C_{ph}), 116.5 (s, o-C_{ph}), 20.1 (s, CH_3). Pyridine ipso carbon not observed.

^{19}F (C_6D_6, 376.5 MHz): δ -89.6 (m, 2F, CF^2), -154.9 (m, 2F, CF^3).

HRMS (APCL): m/z: M$^+$ Calcd for $[C_{12}H_7F_4N_2O]^+$ 258.0537, observed 258.0536.

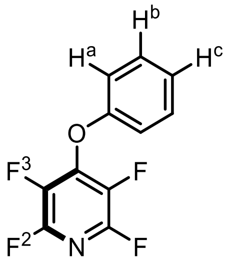

1c. 2,3,5,6-tetrafluoro-4-phenoxypyridine . Isolation achieved by column chromatography in 19:1 n-pentane : dichloromethane, R_F = 0.65. Isolated as a colourless solid. (46.0 mg, 0.23 mmol, 63 %).

^{1}H NMR (C_6D_6, 400 MHz): δ 6.98-6.91 (m, 2H, CH^a), 6.86-6.80 (m, 1H, CH^c), 6.63-6.57 (m, 2H, CH^b)

^{13}C{^{1}H} NMR (C_6D_6, 100 MHz): δ 155.7 (s, O-C_{Ph}), 144.0 (dm, $^1J_{C-F}$ = 247 Hz, CF^2), 143.8 (m, C_{Pyr}-O), 136.0 (dm, $^1J_{C-F}$ = 262 Hz, CF^3), 129.8 (s, CH^b), 124.6 (s, CH^c), 116.3 (s, CH^a).

^{19}F (C_6D_6, 376.5 MHz): δ -89.6 (m, 2F, CF^2), -154.9 (m, 2F, CF^3).

HRMS (APCL): m/z: M^+ Calcd for $[C_{11}H_5F_4NO]^+$ 243.1620, observed 243.1592

Product could not be fully separated from the reagent phenol, with the isolated product ^{1}H NMR showing a 90:10 ratio of 2,3,5,6-tetrafluoro-4-phenoxypyridine : phenol. Here, the isolated yield is based on mass recovery.

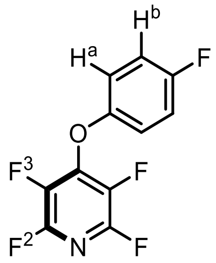

1d. 2,3,5,6-tetrafluoro-4-(4-fluorophenoxy)pyridine Isolation achieved by column chromatography in 19:1 n-pentane : dichloromethane, R_F = 0.50. Isolated as a colourless solid. (42.0 mg, 0.19 mmol, 51 %).

^{1}H NMR (C_6D_6, 400 MHz): δ 6.57-6.52 (m, 2H, CH^a), 6.33-6.28 (m, 2H, CH^b).

^{13}C{^{1}H} NMR (C_6D_6, 100 MHz): δ 159.4 (d, $^1J_{C-F}$ = 233 Hz, C_{Ph}F), 151.5 (s, i-$\underline{C}_{Ph}OC_{Pyr}$), 144.0 (dm, $^1J_{C-F}$ = 246 Hz, CF^2), 135.8 (dm, $^1J_{C-F}$ = 259 Hz, CF^3), 131.6 (s, i-$C_{Pyr}OC_{Ph}$), 118.0 (d, $^3J_{C-F}$ = 9 Hz, CH^a), 116.4 (d, $^2J_{C-F}$ = 24 Hz, CH^b)

^{19}F (C_6D_6, 376.5 MHz): δ -89.1 (m, 2F, CF^2), -117.4 (m, 1F, C_{Ph}F), -154.9 (m, 2F, CF^3).

HRMS (APCL): m/z: M$^+$ Calcd for $[C_{11}H_4F_5NO]^+$ 257.1910, observed 257.1822.

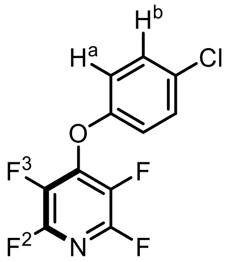

1e. 2,3,5,6-tetrafluoro-4-(4-chlorophenoxy)pyridine. Isolation achieved by column chromatography in 19:1 n-pentane : dichloromethane, R_F = 0.60. Isolated as a colourless solid. (33.5 mg, 0.14 mmol, 38 %).

^{1}H NMR (C_6D_6, 400 MHz): δ 6.85-6.81 (m, 2H, CH^b), 6.70-6.66 (m, 2H, CH^a).

^{13}C{^{1}H} NMR (C_6D_6, 100 MHz): δ 155.0 (s, i-$\underline{C}_{Ph}OC_{Pyr}$), 149.5 (s, i-$C_{Pyr}OC_{Ph}$), 143.7 (dm, $^{1}J_{C-F}$ = 255 Hz, CF^3), 134.1 (dm, $^{1}J_{C-F}$ = 258 Hz, CF^2), 128.5 (s, CH^b), 128.3 (s, C-Cl), 126.0 (s, CH^a).

^{19}F (C_6D_6, 376.5 MHz): δ -88.8 (m, 2F, CF^3), -154.4 (m, 2F, CF^2).

HRMS (APCL): m/z: M^+ Calcd for $[C_{11}H_4F_4NOCl]^+$ 277.9994, observed 277.9990.

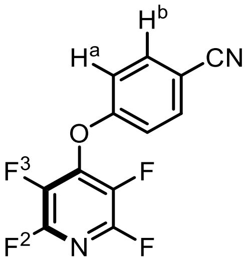

1f. 4-((2,3,5,6-tetrafluoro-pyridin-4-yl)oxy)benzonitrile. Isolation achieved by column chromatography in a gradient column of 19:1 to 1:1 n-pentane : dichloromethane, R_F = 0.1 (in 19:1 mixture). Isolated as a colourless solid. (30.9 mg, 0.11 mmol, 32 %).[S7]

^{1}H NMR (C_6D_6, 400 MHz): δ 6.80-6.75 (m, 2H, CH^b), 6.07-6.02 (m, 2H, CH^a).

^{13}C{^{1}H} NMR (C_6D_6, 100 MHz): δ 159.2 (s, $\underline{C}_{Ph}$-OC_{Pyr}), 148.5 (s, $\underline{C}_{Pyr}$-OC_{Ph}), 141.7 (dm, $^1J_{C\text{–}F}$ = 252 Hz, CF^2), 134.6 (dm, $^1J_{C\text{–}F}$ = 248 Hz, CF^3), 133.8 (s, CH^a), 125.5 (s, CH^b), 116.5 (s, $\underline{C}$-CN), 106.0 (s, CN).

^{19}F (C_6D_6, 376.5 MHz): δ -87.9 (m, 2F, CF^2), 153.5 (m, 2F, CF^3).

IR (cm^{-1}) = 2230 (CN str).

HRMS (APCL): m/z: M^+ Calcd for $[C_{12}H_4F_4N_2O]^+$ 268.0268, observed 268.0268.

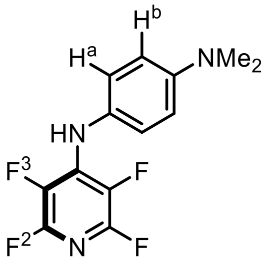

1g. 2,3,5,6-tetrafluoro-N-(4-dimethylaminophenyl)pyridin-4-amine. Isolation achieved by column chromatography in a column of 19:1 n-pentane : dichloromethane, R_F = 0.35. Isolated as a yellow solid. (74.1 mg, 0.26 mmol, 76 %).

^{1}H NMR (C_6D_6, 400 MHz): δ 6.71-6.67 (m, 2H, CH^a), 6.45-6.38 (m, 2H, CH^b), 5.5 (s, 1H, NH), 2.47 (s, 6H, NMe_2).

^{13}C{^{1}H} NMR (C_6D_6, 100 MHz): δ 149.0 (s, C_{Ph}-NMe_2), 144.3 (dm, $^{1}J_{CF}$ = 236.9 Hz, CF^2), 135.5 (m, $\underline{C}_{Pyr}$-NHC_{Ph}), 131.6 (dm, $^{1}J_{CF}$ = 248.9 ,CF^3), 127.1 (s, $\underline{C}_{Ph}$-NHC_{Pyr}), 125.0 (s, CH^a), 112.1 (s, CH^b), 39.8 (s, NMe_2).

^{19}F (C_6D_6, 376.5 MHz): δ -94.1 (m, 2F, CF^2), -158.9 (m, 2F, CF^3).

HRMS (APCL): m/z: M^+ Calcd for $[C_{13}H_{11}F_4N_3]^+$ 286.0961, observed 286.0961.

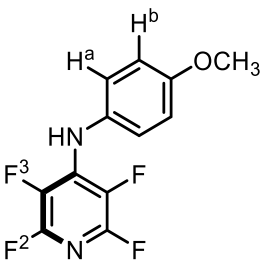

1h. 2,3,5,6-tetrafluoro-N-(4-methoxyphenyl)pyridin-4-amine. Isolation achieved by column chromatography in a gradient column of 19:1 to 1:1 n-pentane : dichloromethane, R_F = 0.48 (in a 1:1 mixture of n-pentane : dichloromethane). Isolated as a colourless solid. (70.7 mg, 0.26 mmol, 72 %).

^{1}H NMR (C_6D_6, 400 MHz): δ 7.09 (d, $^3J_{H-H}$ = 4.9 Hz, 2H, CH^a), 6.63 (m, 2H, CH^b), 5.47 (s, 1H, NH), 3.30 (s, 3H, OCH_3).

^{13}C{^{1}H} NMR (C_6D_6, 100 MHz): δ 157.9 (s, $\underline{C}OCH_3$), 144.2 (dm, $^1J_{C-F}$ = 229 Hz, CF^2), 136.7 (s, $\underline{C}_{Pyr}NHC_{Ph}$), 131.7 (dm, $^1J_{C-F}$ = 249 Hz, CF^3), 132.5 (s, $\underline{C}_{Ph}NHC_{Pyr}$), 124.7 (s, CH^a), 113.9 (s, CH^b), 54.6 (s, OCH_3).

^{19}F (C_6D_6, 376.5 MHz): δ -93.8 (m, 2F, CF^2), -157.3 (m, 2F, CF^3).

HRMS (APCL): m/z: M^+ Calcd for $[C_{12}H_8F_4N_2O]^+$ 273.0646, observed 273.0643.

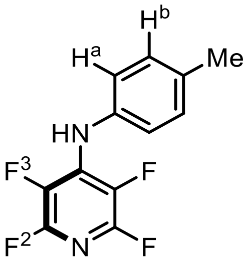

1i. **2,3,5,6-tetrafluoro-N-(p-tolyl)pyridine-4-amine.** Isolation achieved by column chromatography in a column of 19:1 n-pentane : dichloromethane, R_F = 0.15. Isolated as a colourless solid. (74.9 mg, 0.29 mmol, 81 %).

^{1}H NMR (C_6D_6, 400 MHz): δ 6.86-6.78 (m, 2H, CH^a), 6.49-6.41 (m, 2H, CH^b), 5.26 (s, 1H, NH), 2.05 (s, 3H, CH_3).

^{13}C{^{1}H} NMR (C_6D_6, 100 MHz): δ 144.1 (dm, $^1J_{C\text{-}F}$ = 236.3 Hz, CF^2), 135.5 (s, $\underline{C}_{Ph}$-NHC_{Pyr}), 134.9 (s, $\underline{C}_{Ph}$Me), 132.5 (dm, $^1J_{C\text{-}F}$ = 250.1 Hz), 129.2 (s, CH^b), 122.2 (s, CH^a), 20.4 (s, CH_3).

^{19}F (C_6D_6, 376.5 MHz): δ -93.3 (m, 2F, CF^2), -156.2 (m, 2F, CF^3).

HRMS (APCL): m/z: M^+ Calcd for $[C_{12}H_6F_4N_2]^+$ 256.2030, observed 256.2026.

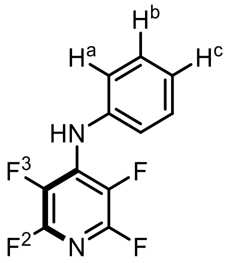

1j. **2,3,5,6-tetrafluoro-N-(phenyl)pyridine-4-amine.** Isolation achieved by column chromatography in a column of 19:1 n-pentane : dichloromethane, R_F = 0.45. Isolated as a colourless solid. (58.1 mg, 0.24 mmol, 68 %).[S8]

^{1}H NMR (C_6D_6, 400 MHz): δ 7.02-6.95 (m, 2H, CH^a), 6.92-6.85 (m, 1H, CH^c), 6.50-6.45 (m, 2H, CH^b).

^{13}C{^{1}H} NMR (C_6D_6, 100 MHz): δ 144.2 (dm, $^1J_{C-F}$ = 232 Hz, CF^2), 138.1 (s, $\underline{C}_{Pyr}NHC_{Ph}$), 132.4 (dm, $^1J_{C-F}$ = 249 Hz), 128.6 (s, CH^b), 125.0 (s, CH^c), 121.6 (s, CH^a). C_{Ph} ipso carbon not observed.

^{19}F (C_6D_6, 376.5 MHz): δ -93.1 (m, 2F, CF^2), -155.2 (m, 2F, CF^3).

HRMS (APCL): m/z: M^+ Calcd for $[C_{12}H_6F_4N_2]^+$ 241.0394, observed 241.0394.

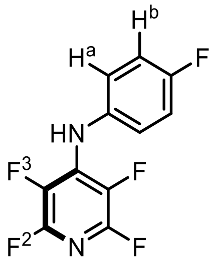

1k. 2,3,5,6-tetrafluoro-N-(4-fluorophenyl)pyridin-4-amine. Isolation achieved by column chromatography in 19:1 n-pentane : dichloromethane, R_F = 0.30. Isolated as a colourless solid. (49.4 mg, 0.19 mmol, 53 %).[S9]

^{1}H NMR (C_6D_6, 400 MHz): δ 6.62 (m, 2H, CH^b), 6.22 (m, 2H, CH^a).

^{13}C{^{1}H} NMR (C_6D_6, 100 MHz): δ 160.5 (d, $^1J_{C–F}$ = 244 Hz, CF^c), 144.1 (dm, $^1J_{C–F}$ = 238 Hz, CF^2), 133.9 (s, i-$\underline{C}_{Ph}$NHC$_{Pyr}$), 132.0 (dm, $^1J_{C–F}$ = 242 Hz, CF^3), 124.2 (d, $^3J_{C–F}$ = 8 Hz, CH^a), 115.3 (d, $^2J_{C–F}$ = 23 Hz, CH^b). $\underline{C}_{Pyr}$NHC$_{Ph}$ Ipso carbon not observed.

^{19}F (C_6D_6, 376.5 MHz): δ -93.0 (m, 2F, CF^2), -116.4 (m, 1F, p-C$_{Ph}$F), -156.4 (m, 2F, CF^3).

HRMS (APCL): m/z: M$^+$ Calcd for $[C_{11}H_5F_5N_2]^+$ 260.0425, observed 260.0418.

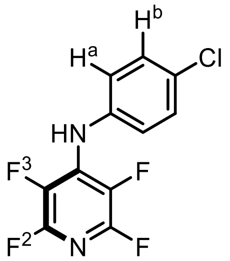

1l. 2,3,5,6-tetrafluoro-N-(4-chlorophenyl)pyridin-4-amine. Isolation achieved by column chromatography in 19:1 n-pentane : dichloromethane, R_F = 0.55. Isolated as a colourless solid. (72.6 mg, 0.25 mmol, 69 %).[S10]

^{1}H NMR (C_6D_6, 400 MHz): δ 6.98-6.92 (m, 2H, CH^b), 6.20-6.13 (m, 2H, CH^a), 5.16 (s, 1H, NH).

^{13}C{^{1}H} NMR (C_6D_6, 100 MHz): δ 144.2 (dm, $^1J_{C-F}$ = 239 Hz, CF^3), 136.5 (s, i-$\underline{C}_{Ph}$-NHC_{Pyr}), 133.1 (m, C_{Ph}-Cl), 132.2 (dm, $^1J_{C-F}$ = 252 Hz, CF^2), 130.4 (s, i-C_{Pyr}-NHC_{Ph}), 128.7 (s, CH^b), 122.9 (s, CH^a).

^{19}F (C_6D_6, 376.5 MHz): δ -92.7 (m, 2F, CF^3), -154.9 (m, 2F, CF^2).

HRMS (APCL): m/z: M^+ Calcd for $[C_{11}H_5F_4N_2Cl]^+$ 276.6241, observed 276.6218.

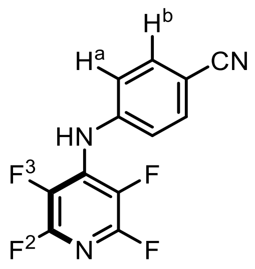

1m. 4-((perfluoropyridin-4-yl)amino)benzonitrile . Isolation achieved by column chromatography in 19:1 n-pentane : dichloromethane, R_F = 0.30. Isolated as a colourless solid. (31.0 mg, 0.12 mmol, 32 %).

^{1}H NMR (C_6D_6, 400 MHz): δ 7.01-6.97 (m, 2H, CH^b), 6.88-6.84 (m, 2H, CH^a), 5.14 (s, NH).

^{13}C{^{1}H} NMR (C_6D_6, 100 MHz): δ 146.0 (s, i-$\underline{C}_{Pyr}$NHC$_{Ph}$), 142.7 (dm, $^1J_{C-F}$ = 252 Hz, CF^2), 134.3 (dm, $^1J_{C-F}$ = 258 Hz), 132.5 (s, CH^b), 132.2 (s, i-$\underline{C}_{Ph}$NHC$_{Pyr}$), 124.1 (s, CH^a), 119.1 (s, CN). $\underline{C}$-CN ipso carbon not observed.

^{19}F (C_6D_6, 376.5 MHz): δ -91.2 (m, 2F, CF^2), -151.9 (m, 2F, CF^3).

IR (cm^{-1}) = 2315.

HRMS (APCL): m/z: M$^+$ Calcd for $[C_{12}H_5F_4N_3]^+$ 266.0351, observed 266.0351.

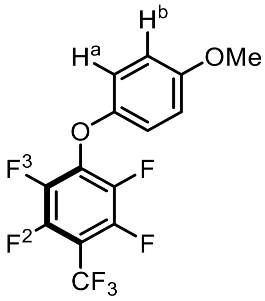

1n. 2,3,5,6-tetrafluoro-4-(4-methoxyphenoxy)-1-(trifluoromethyl)benzene. Isolation achieved by column chromatography in 19:1 n-pentane : dichloromethane, R_F = 0.70. Isolated as a colourless solid. (44.2 mg, 0.13 mmol, 34 %).[S11]

^{1}H NMR (C_6D_6, 400 MHz): δ 6.66-6.61 (m, 2H, CH^a), 6.58-6.53 (m, 2H, CH^b), 3.21 (s, 3H, OCH_3).

^{13}C{^{1}H} NMR (C_6D_6, 100 MHz): δ 156.5 (s, i-C-OMe), 150.5 (s, i-C_{Ph}-$OC_{Fluoroarene}$), 144.5 (d, $^1J_{C–F}$ = 296 Hz, CF^2), 138.2 (d, $^1J_{C–F}$ = 291 Hz, CF^3), 135.6 (i-$C_{Fluoroarene}$-OC_{Ph}), 117.4 (s, CH^b), 114.8 (s, CH^a), 104.6 (m, CF_3), 54.8 (s, OCH_3). C-CF_3 ipso carbon not observed.

^{19}F (C_6D_6, 376.5 MHz): δ -55.6 (t, $^4J_{C–F}$ = 22.9 Hz, CF_3), -141.1 (m, 2F CF^2), -152.9 (m, 2F, CF^3)

HRMS (APCL): m/z: M^+ Calcd for $[C_{14}H_7F_7O_2]^+$ 340.0329, observed 340.0331.

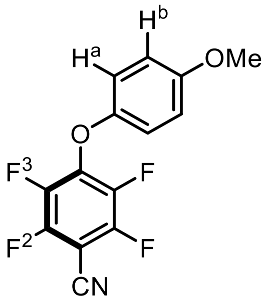

1o. 2,3,5,6-tetrafluoro-4-(4-methoxyphenoxy)-benzonitrile Isolation achieved by column chromatography in 19:1 n-pentane : dichloromethane, R_F = 0.65. Isolated as a colourless solid. (65.3 mg, 0.22 mmol, 58 %).

^{1}H NMR (C_6D_6, 400 MHz): δ 6.71-6.66 (m, 2H, CH^a), 6.62-6.57 (m, 2H, CH^b), 3.24 (s, 3H, OCH_3).

^{13}C{^{1}H} NMR (C_6D_6, 100 MHz): δ 156.5 (s, $\underline{C}$OMe) 117.5 (s, CH^a), 117.2 (s, CH^b), 114.8 (s, CN), 54.9 (s, OMe). C-CN, C_{Pyr}-O, C_{Ph}-O ipso-carbons not observed.

^{19}F (C_6D_6, 376.5 MHz): δ -133.4 (m, 2F, CF^2), -152.3 (m, 2F, CF^3).

IR (cm^{-1}) = 2228 (CN str).

HRMS (APCL): m/z: M$^+$ Calcd for $[C_{14}H_8F_4NO_2]^+$ 298.0486, observed 298.0481.

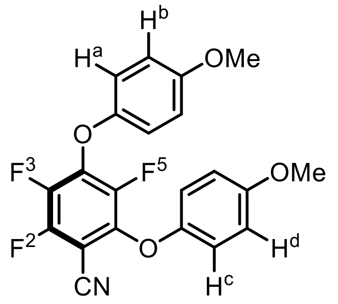

1o'. 2,3,5-trifluoro-4,6-bis(4-methoxyphenoxy)-benzonitrile Isolation achieved by column chromatography in 19:1 pentane : dichloromethane, R_F = 0.61. Isolated as a colourless solid. (12.0 mg, 0.03 mmol, 7 %).

^{1}H NMR (C_6D_6, 400 MHz): δ 6.75-6.71 (m, 2H, H^a), 6.70-6.66 (m, 2H, CH^c), 6.58-6.56 (m, 2H, CH^b), 6.56-6.53 (m, 2H, CH^d), 3.19 (s, 3H, OCH_3), 3.18 (s, 3H, OCH_3).

^{13}C{^{1}H} NMR (C_6D_6, 100 MHz): δ 156.9 (s, $\underline{C}$OMe), 154.9 (s, $\underline{C}$OMe), 150.6 (s, $\underline{C}_{Ph}OC_{Fluoroarene}$), 150.3 (s, $\underline{C}_{Ph}OC_{Fluoroarene}$), 147.6 (dm, $^1J_{C–F}$ = 264 Hz, CF^2), 140.8 (dm, $^1J_{C–F}$ = 257 Hz, CF^3), 136.6 (s, $\underline{C}_{Fluoroarene}OC_{Ph}$), 135.8 (dm, $^1J_{C–F}$ = 261 Hz), 135.3 (s, $\underline{C}_{Fluoroarene}OC_{Ph}$), 117.5 (s, CH^a), 117.1 (s, CH^c), 116.7 (s, CN), 114.9 (s, CH^b), 114.8 (s, CH^d), 107.0 (s, $\underline{C}$-CN), 54.8 (s, OCH_3), 54.6 (s, OCH_3).

^{19}F (C_6D_6, 376.5 MHz): δ -132.2 (m, 1F, CF^2), -142.8 (m, 1F, CF^3), -152.0 (m, 1F, CF^5).

IR (cm^{-1}) = 2230 (CN str).

HRMS (APCL): m/z: M^+ Calcd for 401.0924, observed 401.0921.

Product could not be fully separated from the tri-substituted side reaction product, 4,4',4''-((2,4-difluoro-6-nitrobenzene-1,3,5-triyl)tris(oxy))tris(methoxybenzene). The final ^{19}F NMR spectra shows a 95:5 ratio of 2,3,5-trifluoro-4,6-bis(4-methoxyphenoxy)-benzonitrile : 4,4',4''-((2,4-difluoro-6-nitrobenzene-1,3,5-triyl)tris(oxy))tris(methoxybenzene). Here, the yield of product is based on mass recovery.

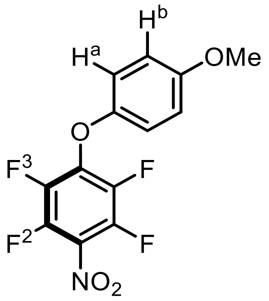

1p. 2,3,5,6-tetrafluoro-4-(4-methoxyphenoxy)-nitrobenzene Isolation achieved by column chromatography in 19:1 n-pentane : dichloromethane, R_F = 0.74. Isolated as a yellow oil. (69.3 mg, 0.22 mmol, 51 %).

^{1}H NMR (C_6D_6, 400 MHz): δ 6.72 (m, 2H, CH^b), 6.57 (m, 2H, CH^a).

^{13}C{^{1}H} NMR (C_6D_6, 100 MHz): δ 156.8 (s, C-OMe), 150.2 (s, C_{Ph}-$OC_{Fluoroarene}$), 142.4 (d, $^1J_{C–F}$ = 269 Hz, CF^3), 136.9 (d, $^1J_{C–F}$ = 267 Hz, CF^2), 129.1 (s, $C_{Fluoroarene}$-OC_{Ph}), 117.7 (s, CH^a), 117.2 (s, C-NO_2), 114.9 (s, CH^b), 54.8 (s, OCH_3).

^{19}F (C_6D_6, 376.5 MHz): δ -146.6 (m, 2F, CF^2), -152.3 (m, 2F, CF^3).

HRMS (APCL): m/z: M^+ Calcd for $[C_{14}H_8F_4NO_2]^+$ 298.0486, observed 298.0481.

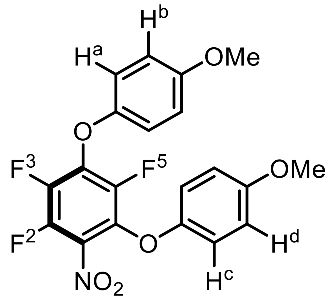

1i'. 2,3,5-trifluoro-4,6-bis(4-methoxyphenoxy)-nitrobenzene Isolation achieved by column chromatography in 19:1 n-pentane : dichloromethane, R_F = 0.70. Isolated as a yellow oil. (16.3 mg, 0.04 mmol, 9 %).

^{1}H NMR (C_6D_6, 400 MHz): δ 6.75-6.69 (m, 2H, CH^a), 6.62-6.52 (m, 6H, overlap of CH$^{b, c, d}$).

^{13}C{^{1}H} NMR (C_6D_6, 100 MHz): δ 156.5 (s, COMe), 156.3 (s, COMe), 151.2 (s, $\underline{C}_{Ph}$-$OC_{Fluoroarene}$) 150.6 (s, $\underline{C}_{Ph}$-$OC_{Fluoroarene}$), 117.1 (s, CH^a), 117.0 (s, CH^c), 114.8 (s, CH^b), 114.7 (s, CH^d). $\underline{C}_{Fluoroalkene}$-$OC_{Ph}$ and C-NO_2 ipso carbons not observed.

^{19}F (C_6D_6, 376.5 MHz): δ -141.9 (m, 1F, CF^2), -147.8 (m, 1F, CF^3), -151.0 (m, 1F, CF^5).

HRMS (APCL): m/z: M$^+$ Calcd for 421.0802, observed 421.0804.

Product could not be separated from pentafluoronitrobenzene starting material, with the final ^{19}F NMR spectra showing a 92:8 ratio of 2,3,5-trifluoro-4,6-bis(4-methoxyphenoxy)-nitrobenzene : pentafluoronitrobenzene. Here, the yield of product is based on mass recovery.

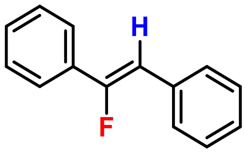

2a. (Z)-(1-Fluoroethene-1,2-diyl)dibenzene. Isolation achieved by column chromatography in 19:1 n-pentane : dichloromethane (R_F = 0.85).[S12] Product could not be fully separated from the starting material diphenylacetylene, with the final ^{1}H NMR spectra showing a ratio of 2.4:1 (Z)-(1-Fluoroethene-1,2-diyl)dibenzene : diphenylacetylene. Isolated as a colourless solid with mass recovery of 41.6 mg.

^{1}H NMR (C_6D_6, 400 MHz): δ 7.51 (m, 4H, ArH), 7.18 (t, $^3J_{H–H}$ = 7.5 Hz, 1H, ArH), 7.03 (m, 5H, ArH), 6.10 (d, $^3J_{H–F}$ = 39.6 Hz, C_{vinyl}H).

^{13}C{^{1}H} NMR (C_6D_6, 100 MHz): δ 157.2 (d, $^1J_{C–F}$ = 254 Hz, C_{alkene}F) 133.8 (d, $^3J_{C–F}$ = 3 Hz, i-C_{Ph}), 132.9 (d, $^2J_{CF}$ = 27 Hz, i-C_{Ph}), 129.0 (d, $^3J_{C–F}$ = 6 Hz, C_{Ph}H), 128.7 (s, C_{Ph}H), 128.5 (s, C_{Ph}H), 128.4 (s, C_{Ph}H), 127.3 (d, $^5J_{C–F}$ = 3 Hz, C_{Ph}H), 124.3 (d, $^4J_{C–F}$ = 8 Hz, C_{Ph}H), 106.1 (d, $^2J_{C–F}$ = 9 Hz, C_{alkene}H).

^{19}F (C_6D_6, 376.5 MHz): δ -114.4 (d, $^3J_{H–F}$ = 39 Hz).

HRMS (APCL): m/z: M^+ Calcd for $[C_{14}H_{11}F]^+$ 198.0839, observed 198.0839.

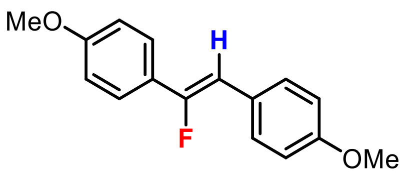

2c. (Z)-4,4'-(1-fluoroethene-1,2-diyl)bis(methoxybenzene). Isolation achieved by column chromatography in 9:1 hexane : ethyl acetate (R_F = 0.6).[S12] Product could not be fully separated from the starting material diphenylacetylene, with the final ^{1}H NMR spectra showing a ratio of 82:18 (Z)-4,4'-(1-fluoroethene-1,2-diyl)bis(methoxybenzene) : 1,2-bis(4-methoxyphenol)ethylene. Isolated as a colourless solid with mass recovery of 70.2 mg.

^{1}H NMR (C_6D_6, 400 MHz): δ 7.66-7.61 (m, 2H, ArH), 7.48-7.43 (m, 2H, ArH), 6.85-6.80 (m, 2H, ArH), 6.73-6.68 (m, 2H, ArH), 6.07 (d, $^3J_{HF}$ = 40.7 Hz, C_{alkene}H), 3.29 (s, 3H, OCH_3), 3.23 (s, 3H, OCH_3).

^{13}C{^{1}H} NMR (C_6D_6, 100 MHz): δ 158.3 (d, $^1J_{C–F}$ = 273 Hz, C_{alkene}F), 156.0 (s, C-OMe), 155.9 (s, C-OMe), 130.3 (s, C_{Ph}H), 130.2 (s, C_{Ph}H), 129.7 (s, $\underline{C}_{Ph}$-C_{Vinyl}H), 125.6 (d, $^2J_{C–F}$ = 9 Hz, $\underline{C}_{Ph}$-C_{vinyl}F), 114.1 (s, C_{Ph}H), 114.0 (s, C_{Ph}H), 104.2 (d, $^2J_{C–F}$ = 11 Hz, C_{alkene}H), 54.5 (s, OMe), 54.4 (s, OMe).

^{19}F (C_6D_6, 376.5 MHz): δ -116.3 (d, $^3J_{HF}$ = 40.2 Hz, C_{alkene}F).

HRMS (APCL): m/z: M^+ Calcd for 258.1142, observed 258.1140.

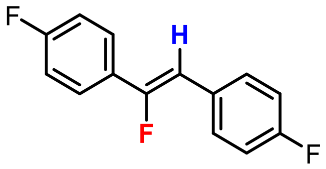

2d. (Z)-4,4'-(1-fluoroethene-1,2-diyl)bis(fluorobenzene). Isolation achieved by column chromatography in 19:1 n-hexane : ethylacetate (R_F = 0.6). Isolated as a colourless solid. (53.6 mg, 0.25 mmol, 57 %).

^{1}H NMR (C_6D_6, 400 MHz): δ 7.38-7.33 (m, 2H, C_{Ph}H), 7.25-7.20 (m, 2H, C_{Ph}H), 6.85-6.79 (m, 2H, C_{Ph}H), 6.73-6.66 (m, 2H, C_{Ph}H), 5.76 (d, $^{3}J_{H-F}$ = 39 Hz, 1H, C_{vinyl}H),

^{13}C{^{1}H} NMR (C_6D_6, 100 MHz): δ 162.5 (d, $^{1}J_{C-F}$ = 253 Hz, C_{Ph}F), 156.3 (d, $^{1}J_{C-F}$ = 260 Hz, C_{vinyl}F), 133.5 (s, $\underline{C}_{Ph}$-C_{vinyl}H), 133.3 (s, $\underline{C}_{Ph}$-C_{vinyl}F), 115.7 (s, C_{Ph}H), 115.6 (s, C_{Ph}H), 115.5 (s, C_{Ph}H), 115.4 (s, C_{Ph}H), 88.1 (s, C_{vinyl}H).

^{19}F (C_6D_6, 376.5 MHz): δ -115.0 (d, $^{3}J_{H-F}$ = 39 Hz, 1F, C_{vinyl}F), -111.7 (m, 1F, C_{Ph}F), -113.3 (m, 1F, C_{Ph}F).

HRMS (APCL): m/z: M^+ Calcd for $[C_{14}H_9F_3]^+$ = 234.0652, observed 234.0652.

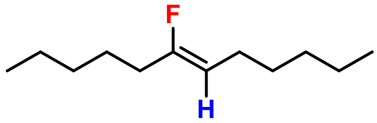

2e. (Z)-6-fluorododec-6-ene. Isolation achieved by column chromatography in pentane (R_F = 0.8). Isolated as a colourless oil (64 mg, 0.35 mmol, 79 %).[S7]

^{1}H NMR (C_6D_6, 400 MHz): δ 4.36 (dt, $^3J_{H–F}$ = 37.7 Hz, 1H, C_{vinyl}H), 2.17-2.09 (m, 2H), 2.05-1.95 (m, 2H), 1.45-1.11 (m, 12H), 0.92-0.80 (m, 6H).

^{13}C{^{1}H} NMR (C_6D_6, 100 MHz): δ 159.7 (d, $^1J_{C–F}$ = 252 Hz, CF), 104.8 (d, $^2J_{C–F}$ = 16.7 Hz, C_{Vinyl}H), 32.0 (d, $^2J_{C–F}$ = 29.6 Hz), 31.2 (d, $^3J_{C–F}$ = 29 Hz), 29.4 (s, CH_2), 26.0 (s, CH_2), 23.6 (s, CH_2), 23.5 (s, CH_2), 22.5 (s, CH_2), 22.4 (s, CH_2), 13.9 (s, CH_3), 13.8 (s, CH_3).

^{19}F NMR (C_6D_6, 376.5 MHz): δ -109.3 (m, 1F).

HRMS (APCL): m/z: M^+ Calcd for $[C_{12}H_{23}F]^+$ = 204.1309, observed 204.1318.

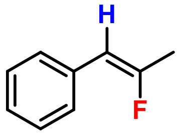

2f. (Z)-(2-fluoroprop-1-en-1-yl)benzene. Isolation achieved by column chromatography in pentane (R_F = 0.7). Isolated as a colourless oil (39 mg, 0.28 mmol, 64 %).[S14]

^{1}H NMR (C_6D_6, 400 MHz): δ 7.50 (m, 2H C_{Ph}H), 7.17 (m, 2H, C_{Ph}H), 7.02 (m, 1H, C_{Ph}H), 5.13 (d, $^3J_{H-F}$ = 39.0 Hz, 1H, C_{vinyl}H), 1.57 (d, $^3J_{H-F}$ = 17.0, 3H, CH_3).

^{13}C{^{1}H} NMR (C_6D_6, 100 MHz): δ 157.4 (d, $^1J_{C-F}$ = 263.7 Hz, C_{vinyl}F), 134.0 (s, $\underline{C}_{Ph}$-CH), 128.4 (s, C_{Ph}), 128.2 (s, C_{Ph}), 126.6 (s, C_{Ph}), 106.5 (d, $^2J_{C-F}$ = 8.9 Hz, C_{vinyl}H), 18.3 (d, $^2J_{C-F}$ = 29.5 Hz, CH_3).

^{19}F NMR (C_6D_6, 376.5 MHz): δ -94.8 (m, 1F).

HRMS (APCL): m/z: M^+ Calcd for $[C_9H_9F]^+$ = 136.0683, observed 136.0689.

The product was separated from the minor product (Z)-(1-fluoroprop-1-en-1-yl)benzene through column chromatography. The mixture of products is formed in a 20:1 ratio as determined by quantitative ^{19}F NMR:

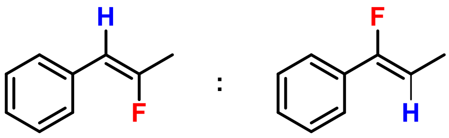

20 : **1**

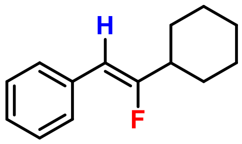

2g. (Z)-(2-cyclohexyl-2-fluorovinyl)benzene. Isolation achieved by column chromatography in pentane (R_F = 0.8). Isolated as a colourless oil (73 mg, 0.36 mmol, 82 %).[S15]

^{1}H NMR (C_6D_6, 400 MHz): δ 7.61-7.56 (m, 2H, $C_{Ph}H$), 7.21-7.14 (m, 2H, $C_{Ph}H$), 7.07-7.01 (m, 1H, $C_{Ph}H$), 5.32 (d, $^3J_{H-F}$ = 40.7 Hz, 1H, $C_{vinyl}H$), 2.04-1.91 (m, 1H, $C_{alkyl}H$), 1.82-1.73 (m, 2H, $C_{alkyl}H$), 1.63-1.53 (m, 2H, $C_{alkyl}H$), 1.34-0.93 (m, 6H, $C_{alkyl}H$).

^{13}C{^{1}H} NMR (C_6D_6, 100 MHz): δ 165.0 (d, $^1J_{C-F}$ = 266.0 Hz, $C_{vinyl}F$), 134.2 (s, $\underline{C}_{Ph}$-$C_{vinyl}H$), 128.6 (s, C_{Ph}), 128.4 (s, C_{Ph}), 126.6 (s, C_{Ph}), 104.0 (d, $^2J_{C-F}$ = 9.0 Hz, $C_{vinyl}H$), 41.5 (d, $^2J_{C-F}$ = 24.7 Hz, $C_{alkyl}H$-$C_{vinyl}F$), 29.9 (s, $C_{alkyl}H_2$), 25.8 (s, $C_{alkyl}H_2$), 25.7 (s, $C_{alkyl}H_2$).

^{19}F NMR (C_6D_6, 376.5 MHz): δ -105.6 (dd, $^3J_{H-F}$ = 40.8 Hz, $^4J_{H-F}$ = 16.1 Hz, 1F).

HRMS (APCL): m/z: M^+ Calcd for $[C_{14}H_{17}F]^+$ = 186.1778, observed 186.1780.

The product was separated from the minor product (Z)-(2-cyclohexyl-1-fluorovinyl)benzene through column chromatography. The mixture of products is formed in a 14:1 ratio as determined by quantitative ^{19}F NMR:

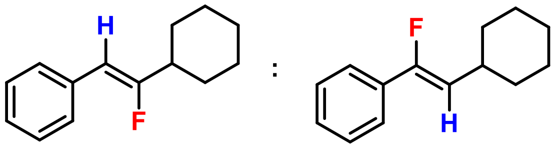

14 : **1**

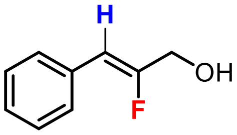

2h. (Z)-2-fluoro-3-phenylprop-2-en-1-ol. Isolation achieved by column chromatography in dichloromethane (R_F = 0.4). Isolated as an orange oil (36 mg, 0.24 mmol, 54 %).[S16]

^{1}H NMR (C_6D_6, 400 MHz): δ 7.52-7.47 (m, 2H, C_{Ph}H), 7.17-7.13 (m, 2H, C_{Ph}H), 7.05-7.00 (m, 1H, C_{Ph}H), 5.45 (d, $^{3}J_{H\text{–}F}$ = 39.5 Hz, 1H, C_{vinyl}H), 3.70 (dd, $^{3}J_{H\text{–}F}$ = 12.8 Hz, $^{4}J_{H\text{–}H}$ = 5.5 Hz, 2H, CH_2).

^{13}C{^{1}H} NMR (C_6D_6, 100 MHz): δ 158.8 (d, $^{1}J_{C\text{–}F}$ = 266.3 Hz, C_{vinyl}F), 133.1 (s, $\underline{C}_{Ph}$-CH), 128.6 (s, C_{Ph}), 128.4 (s, C_{Ph}), 127.3 (s, C_{Ph}), 106.7 (d, $^{2}J_{C\text{–}F}$ = 6.4 Hz, C_{vinyl}H), 61.1 (d, $^{2}J_{C\text{–}F}$ = 35.1 Hz, CH_2).

^{19}F NMR (C_6D_6, 376.5 MHz): δ -112.9 (m, 1F).

HRMS (APCL): m/z: M^+ Calcd for $[C_9H_9FO]^+$ = 152.0632, observed 152.0632.

The product could not be fully separated from the minor product (Z)-3-fluoro-phenylprop-2-en-1-ol, with the columned mixture being a mixture of products with a ratio of 1:0.05 determined by quantitative ^{19}F NMR. Prior to column chromatography, the two fluoroalkene products are observed in a ratio of 6:1.

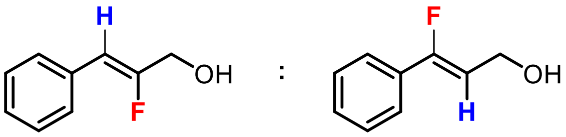

6 : **1**

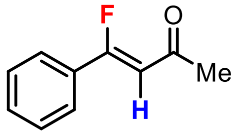

2i. (Z)-4-fluoro-4-phenylbut-3-en-2-one. Isolation achieved by column chromatography in dichloromethane (R_F = 0.3). [S17] Isolated as a pale yellow oil (31 mg, 0.19 mmol, 32 %).

^{1}H NMR (C_6D_6, 400 MHz): δ 7.20-7.16 (m, 2H, C_{Ph}H), 7.00-6.95 (m, 1H, C_{Ph}H), 6.93-6.87 (m, 2H, C_{Ph}H), 5.87 (d, $^3J_{H\text{–}F}$ = 39.8 Hz, 1H, C_{vinyl}H), 2.22 (d, $^5J_{H\text{–}F}$ = 4.2 Hz, 3H, CH_3).

^{13}C{^{1}H} NMR (C_6D_6, 100 MHz): δ 194.1 (s, <u>C</u>OMe), 164.5 (d, $^1J_{C\text{–}F}$ = 270.0 Hz, C_{vinyl}F), 130.8 (s, C_{Ph}), 130.5 (d, $^2J_{C\text{–}F}$ = 28.3 Hz, C_{Ph}-CF), 128.5 (s, C_{Ph}), 125.5 (d, $^3J_{C\text{–}F}$ = 8.7 Hz, C_{Ph}H), 107.3 (d, $^2J_{C\text{–}F}$ = 10.8 Hz, C_{vinyl}H), 30.8 (d, $^4J_{C\text{–}F}$ = 7.2 Hz, CO<u>C</u>H$_3$).

^{19}F NMR (C_6D_6, 376.5 MHz): δ -99.0 (dm, $^3J_{H\text{–}F}$ = 40.1 Hz, C_{vinyl}F).

HRMS (APCL): m/z: M$^+$ Calcd for $[C_{10}H_9FO]^+$ = 164.0584, observed 164.0580.

No alternative regiomer of the product 2i was observed.

3.2. Substrate Competition Reactions

Competition reactions between nucleophiles in the absence of, or presence of, 0.1 equivalents of [Au(IPr)N^{i}Pr$_2$] catalyst were conducted as outlined in Figure S2.

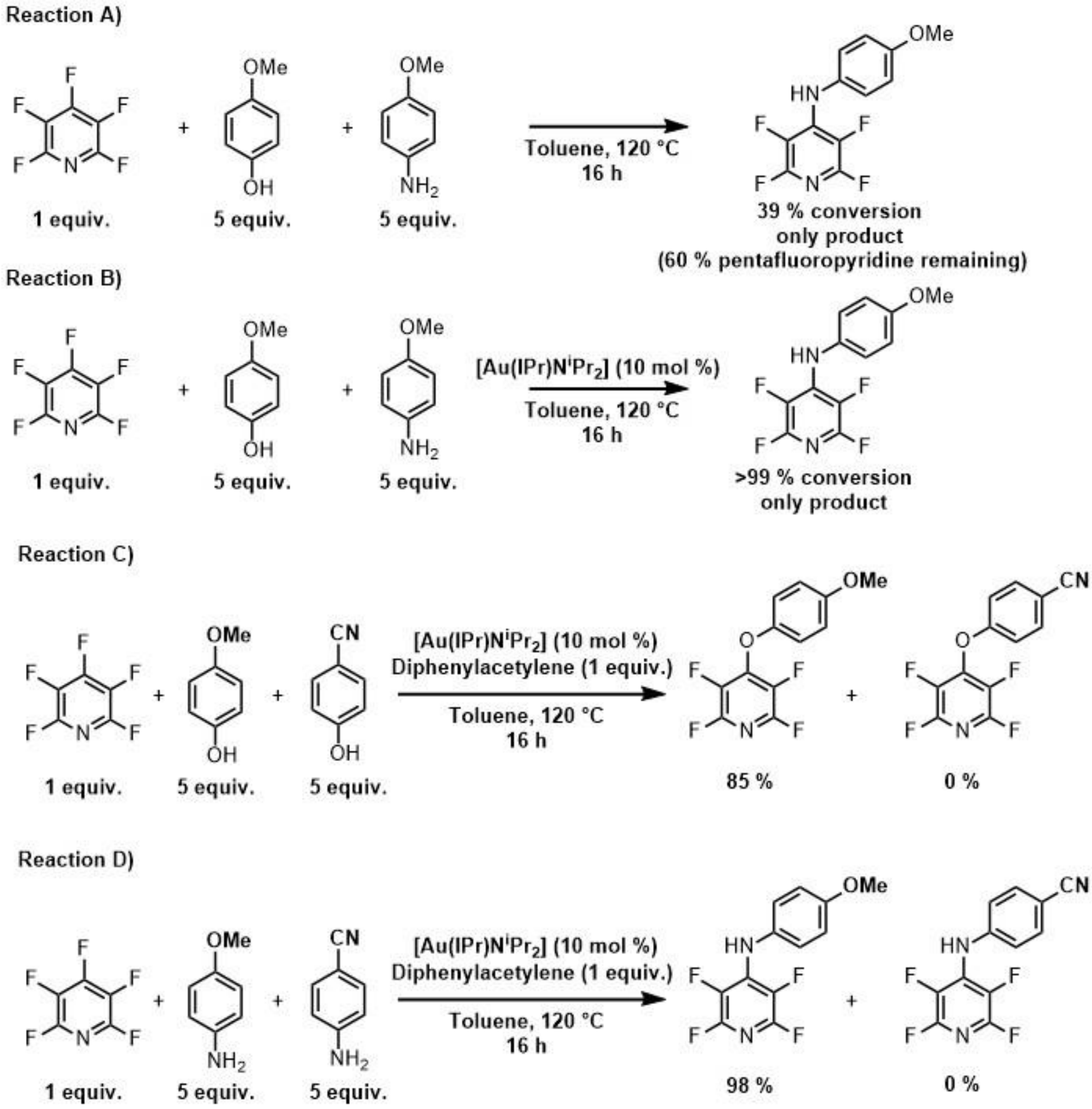

Figure S2. Reactions were conducted with 1:5:5 equivalents of pentafluoropyridine (0.036 mmol, 0.04 M) : nucleophiles in 0.5 ml of toluene. In reactions B, 10 mol % of [Au(IPr)N^{i}Pr$_2$] precatalyst was included. Yields and conversions were calculated from ^{19}F NMR, in the presence of monofluorobenzene as an internal standard.

4. DFT Studies

DFT calculations were run using Gaussian 09 (Revision D.01) using the PBE0, B3PW91 and wB97X-D density functionals. The 6-31G** basis set was used for all atoms except Au. Geometry optimisation calculations were performed without symmetry constraints. The pseudo potential SDDAll was applied for Au only. Frequency analyses for all stationary points were performed to confirm the nature of the structures as either minima (no imaginary frequency) or transition states (only one imaginary frequency). Intrinsic reaction coordinate (IRC) calculations followed by full geometry optimisations on final points were used to connect transition states and minima located on the potential energy surface allowing a full energy profile (calculated at 298.15 K, 1 atm) of the reaction to be constructed. Free energies reported are corrected for the effects of toluene (Ɛ=2.38) solvent using the polarizable continuum model (PCM). Calculations run using the PBE0 or B3PW91 functionals were corrected with GD3-BJ empirical dispersion inclusion. Solvent and dispersion corrections were run in transition state and intermediates optimisation unless stated otherwise. The graphical user interface used to visualise the various properties of the intermediates and transition states was GaussView 5.0.9.

Two reaction profiles were considered for the Au catalysed reaction of pentafluoropyridine, diphenylacetylene and either 4-methoxyphenol or 4-methoxyaniline.

4.1 Calculated Reaction Pathway with 4-Methoxyphenol Mechanism

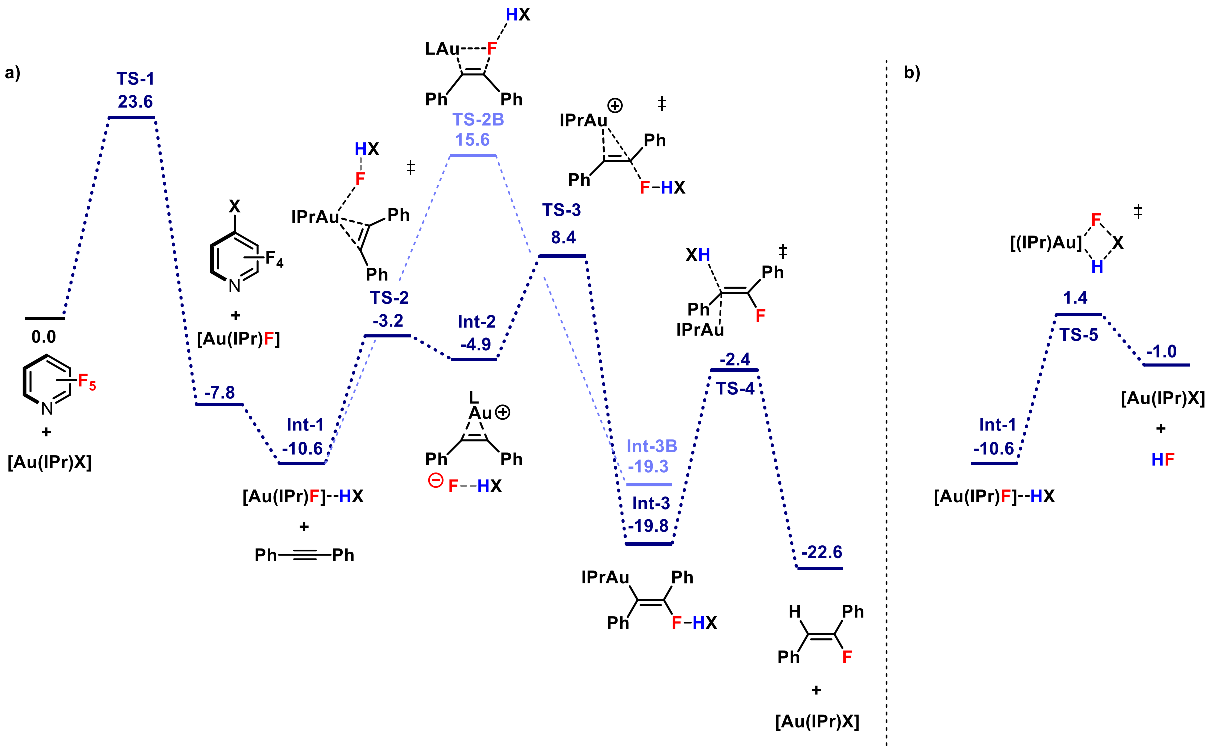

Figure S3. a) Proposed reaction pathway for the [Au(IPr)X] (x = $OC_6H_4OCH_3$) catalysed HF shuttle reaction of pentafluoropyridine and diphenylacetylene with 4-methoxyphenol and b) The side reaction of **Int-2**. Energy values stated in Kcal mol^{-1} and calculated using the PBE0 functional and 6-311G** basis set.

4.2 Functional and Basis-Set Scope Testing for 4-Methoxyphenol Mechanism

	B3PW91/ 6-31G**	ωB97XD / 6-31G**	PBE0 / 6-31G**	PBE0 / 6-311G**
TS-1	20.5	19.6	21.8	23.6
[Au(IPr)F]	-9.9	-7.5	-8.7	-7.8
Int-1	-12.7	-9.4	-11.5	-10.6
TS-2	-6.5	-2.8	-3.6	-3.2
TS-2B	11.7	12.4	14.8	15.6
Int-2	-7.8	-4.6	-5.2	-4.9
TS-3	5.8	6.2	7.9	8.4
Int-3	-22.6	-18.2	-20.5	-19.8
Int-3B	-22.1	-17.6	-20.1	-19.3
TS-4	-7.6	-3.6	-4.1	-2.4
TS-5	-3.1	-2.5	-0.3	1.4
Product	-26.7	-25.4	-23.1	-22.6

Table S4. Calculated energies for the intermediates and transition states shown in Figure S3. [a] Values calculated with empirical dispersion = GD3-BJ and solvent interaction scrf=(pcm, solvent=toluene) included in the optimisation. [b] Values calculated with single point solvent and dispersion after structure optimisation. [c]The 6-311G** basis set was applied as a single point energy correction to 6-31G** / PBE0 values.

4.3 Geometries of Intermediates and Transition-States in the Paramethoxyphenol Mechanism

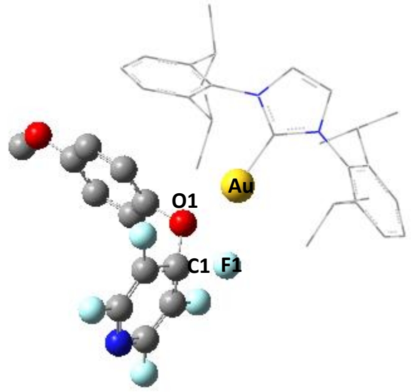

TS-1

C1-F1 = 1.64 Å
C1-O1 = 1.44 Å
F1-C1-O1 = 89.9°

q (Au) = 0.52
q (O1) = -0.61
q (F1) = -0.51
q (C1) = 0.40

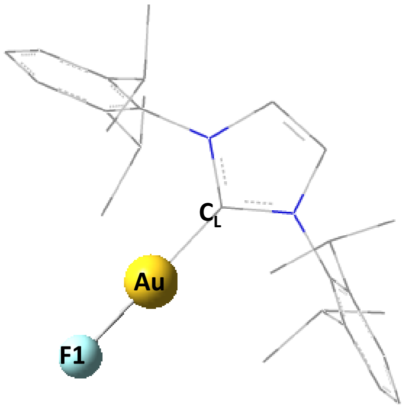

[Au(IPr)F]

Au-F1 = 1.97 Å
Au-C_L = 1.96 Å
F1-Au-C_L = 180.0°

q (Au) = 0.43
q (F1) = -0.68

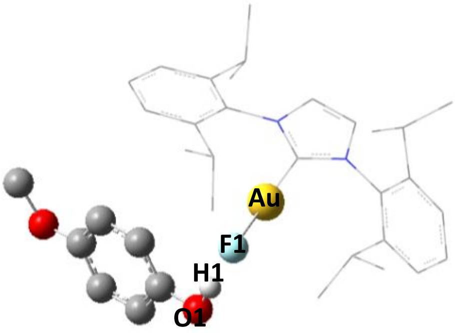

Int-1

Au-F1 = 2.01 Å
Au-C_L = 1.96 Å
F1-H1 = 1.59 Å

q (Au) = 0.45
q (F1) = -0.67
q (O1) = -0.75

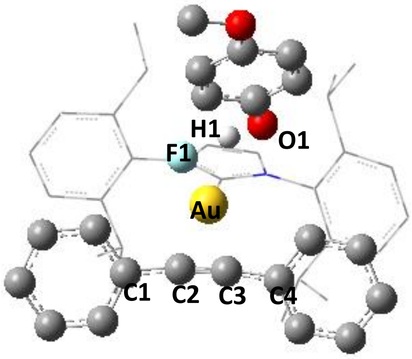

TS-2

Au-F1 = 2.50 Å
Au-C3 = 2.25 Å
C1-C2-C3 = 170.3°
Au–C3 bond order = 0.34

q (Au) = 0.57
q (F1) -0.71
q (C3) = -0.03

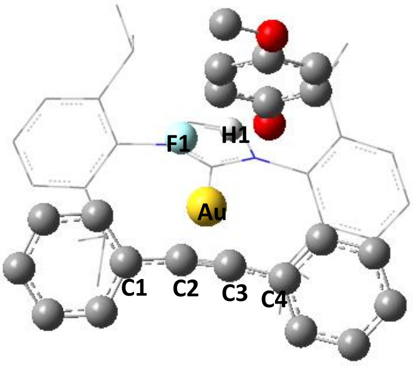

Int-2

Au-F1 = 2.51 Å
Au-C3 = 2.26 Å
C1-C2-C3 = 167.2°
Au–C2 bond order = 0.46

q (Au) = 0.56
q (F1) = -0.72
q (C3) = -0.04
Au-C3 bond order = 0.46

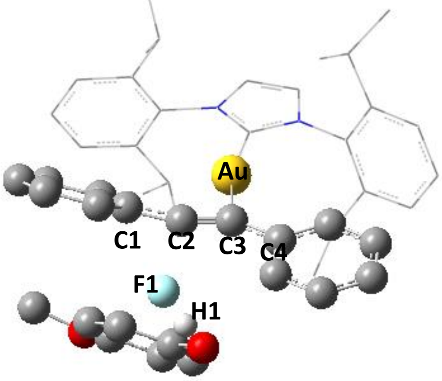

TS-3

Au-C3 = 2.13 Å
F1-C3 = 2.33 Å
C1-C2-C3 = 166.6°
Au–C3 bond order = 0.55

q (Au) = 0.47
q (F1) = -0.69
q (C3) = -0.20

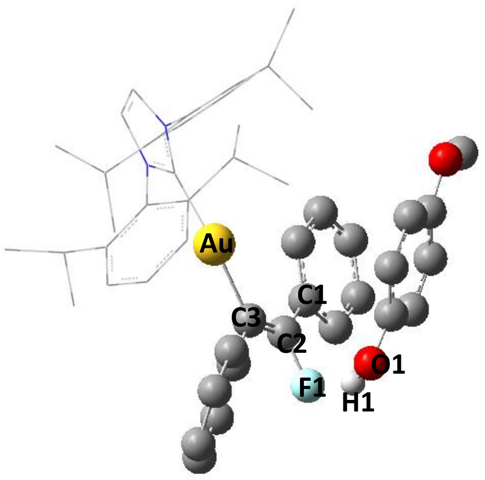

Int-3

Au-C3 = 2.04 Å	q (Au) = 0.35
C2-F1 = 1.39 Å	q (F1) = -0.38
F1-H1 = 2.10 Å	q (C2) = 0.36
C1-C2-C3 = 132.6°	q (C3) = -0.44
Au–C3 bond order = 0.73	q (O1) =
	q (H1) =

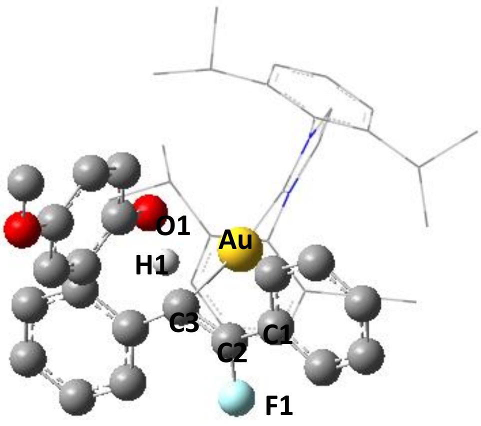

TS-4

O1-H1 = 1.74 Å	q (Au) = 0.47
H1-C3 = 1.12 Å	q (F1) = -0.34
C3-Au = 2.23 Å	q (C2) = 0.48
H1-C3-Au = 99.5°	q (C3) = -0.57
Au–C3 bond order =0.51	q (O1) = -0.81
	q (H1) = 0.40

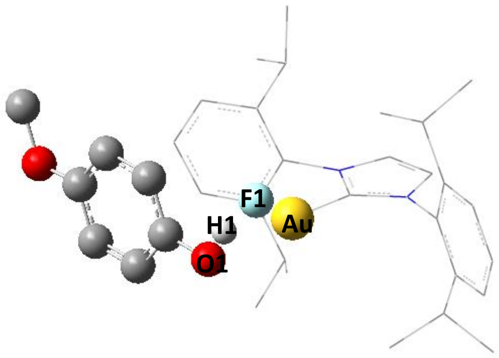

TS-5

Au-F = 2.39 Å	q(Au) = 0.52
F-H = 1.21 Å	q(F1) = -0.69
O-Au = 2.33 Å	q(O1) = -0.82
O-H = 1.13 Å	q(H1) = 0.54

Table S5. Optimised structures of transition states and intermediates of the reaction energy profile detailed in Figure S3. Selected bond lengths and angles are provided below each structure. Full reaction coordinates for each structure can be found under XYZ coordinates. Protons and IPr ligand removed for clarity.

4.4 Calculated Reaction Pathway with 4-Methoxyaniline Mechanism

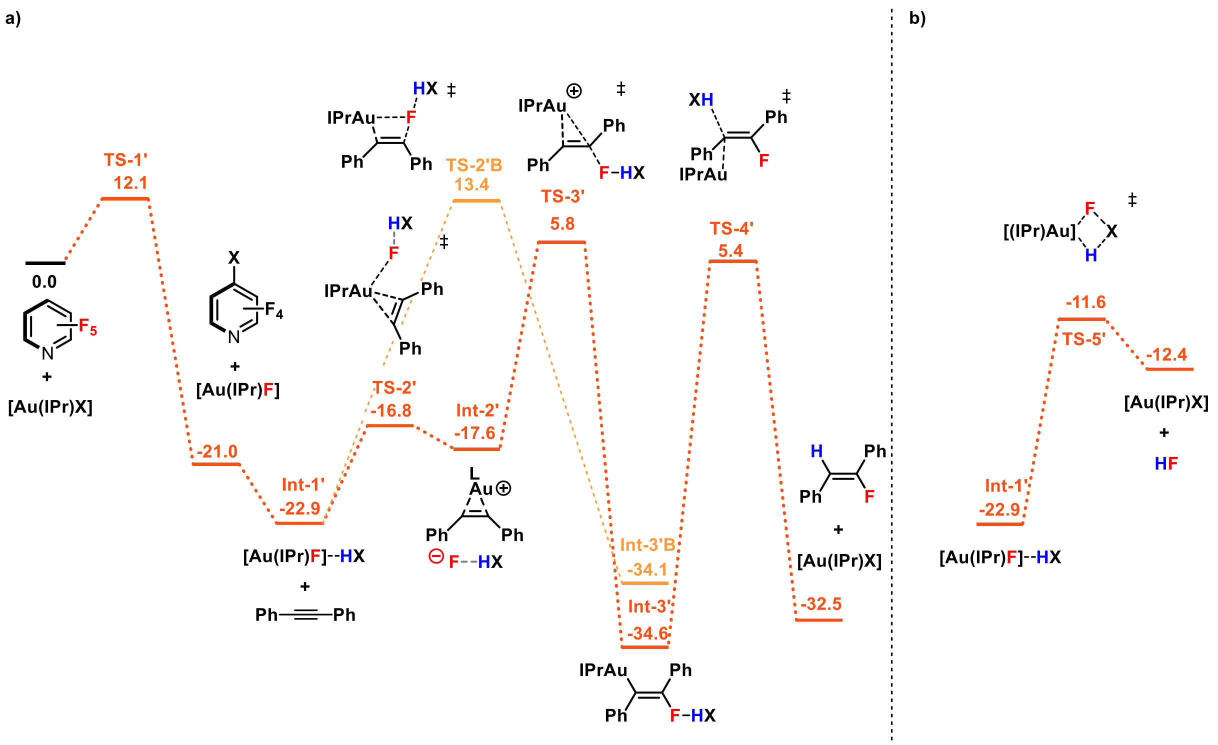

Figure S4. a) Proposed reaction pathway for the [Au(IPr)X] (x = $NHC_6H_4OCH_3$) catalysed HF shuttle reaction of pentafluoropyridine and diphenylacetylene with 4-methoxyaniline and b) The side reaction of **Int-2**. Energy values stated in Kcal mol^{-1} and calculated using the PBE0 functional and 6-311G** basis set.

4.5 Functional Testing for Paranethoxyaniline Mechanism

	B3PW91/ 6-31G**	ωB97XD / 6-31G**	PBE0 / 6-31G**	PBE0 / 6-311G**
TS-1’	9.4	12.1	8.6	12.1
[Au(IPr)F]	-25.3	-24.2	-24.3	-21.0
Int-1’	-28.0	-26.4	-26.5	-22.9
TS-2’	-21.5	-19.4	-20.4	-16.8
Int-2’	-22.7	-20.3	-21.2	-17.6
Int-2’B	15.4	10.8	9.5	13.4
TS-3’	0.4	1.6	2.4	5.8
Int-3’	-32.6	-35.0	-38.1	-34.6
Int-3’B	-32.1	-34.6	-37.3	-34.1
TS-4’	0.5	1.6	0.9	5.4
TS-5’	-16.4	-14.9	-15.2	-11.6
Product	-34.6	-34.3	-36.0	-32.5

Table S6. Calculated energies for the intermediates and transition states shown in Figure S4. [a] Values calculated with empirical dispersion = GD3-BJ and solvent interaction scrf=(pcm, solvent=toluene) included in the optimisation. [b] Values calculated with single point solvent and dispersion after structure optimisation. [c]The 6-311G** basis set was applied as a single point energy correction to 6-31G** / PBE0 values.

4.6 Geometries of Intermediates and Transition-States in the 4-Methoxyaniline Mechanism

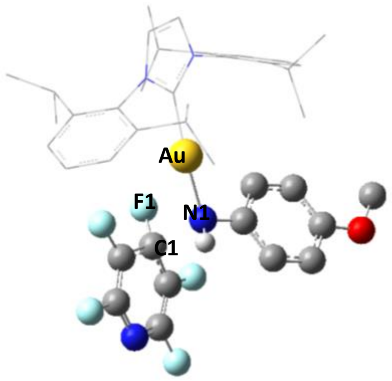

TS-1'

C1-F1 = 1.36 Å
C1-N1 = 1.92 Å
F1-C1-N1 = 94.1°

q (Au) = 0.42
q (N1) = -0.87
q (F1) = -0.36
q (C1) = 0.38

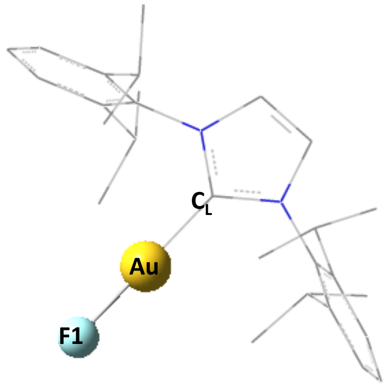

[Au(IPr)F]

Au-F1 = 1.97 Å
Au-C_L = 1.96 Å
F1-Au-C_L = 180.0°

q (Au) = 0.43
q (F1) = -0.68

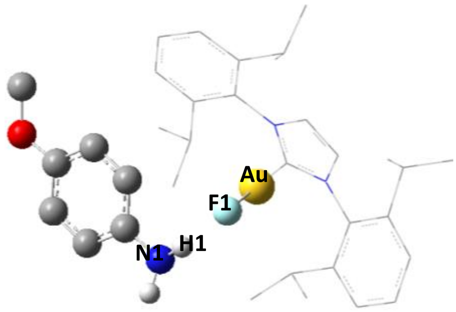

Int-1'

Au-F1 = 2.00 Å
Au-C1 = 1.96 Å
F1-H1 = 1.82 Å
F1-Au-C_L = 180.0°

q (Au) = 0.45
q (F1) = -0.68
q (N1) = -0.89

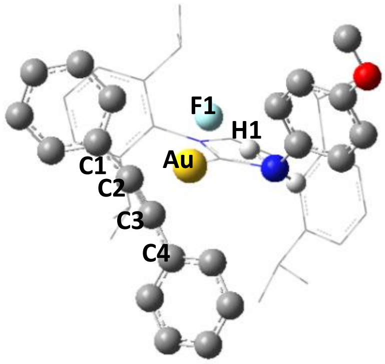

TS-2'

Au-F1 = 2.32 Å
Au-C2 = 2.38 Å
Au-C3 = 2.41 Å
C1-C2-C3 = 169.5°

q (Au) = 0.58
q (F1) = -0.73
q (C3) = -0.04

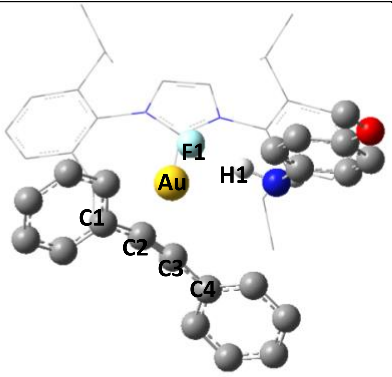

Int-2'

Au-F1 = 2.42 Å
Au-C2 = 2.30 Å
Au-C3 = 2.27 Å
C1-C2-C3 = 167.3°
Au–C2 bond order = 0.45

q (Au) = 0.58
q (F1) = -0.73
q (C3) = --0.05
Au-C3 bond order = 0.46

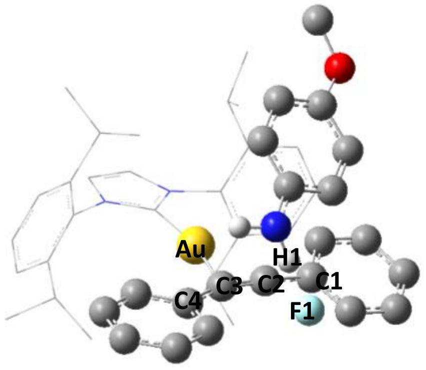

TS-3'

Au-C3 = 2.14 Å
F-C2 = 2.51 Å
F1-H1 = 1.58 Å
C1-C2-C3 = 172.4°
Au–C3 bond order = 0.54

q (Au) = 0.47
q (F1) = -0.73
q (C3) = -0.17

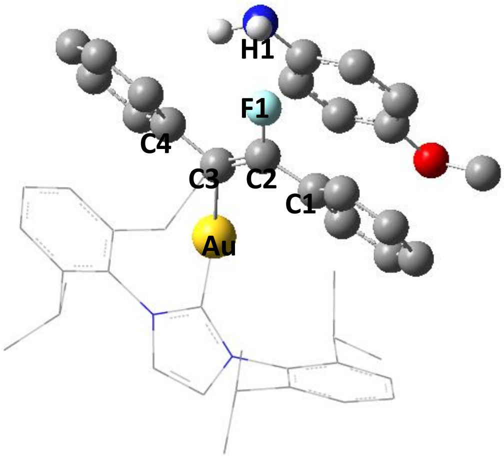

Int-3’

Au-C3 = 2.05 Å	q (Au) = 0.34
C2-F1 = 1.38 Å	q (F1) = -0.37
F1-H1 = 2.36 Å	q (C2) = 0.38
C1-C2-C3 = 129.4°	q (C3) = -0.46
Au–C3 bond order = 0.73	

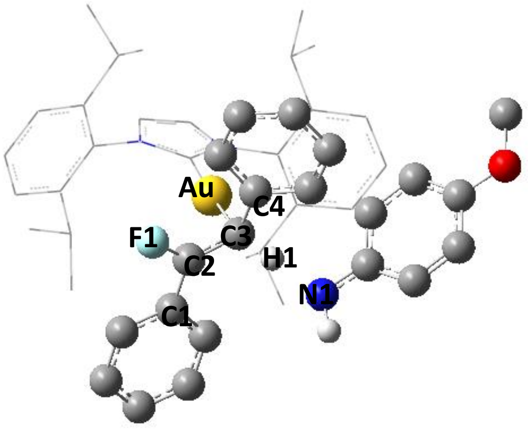

TS-4’

N1-H1 = 1.74 Å	q (Au) = 0.46
H1-C3 = 1.16 Å	q (F1) = -0.34
C3-Au = 2.20 Å	q (C2) = 0.49
H1-C3-Au = 94.5°	q (C3) = -0.55
Au–C3 bond order = 0.51	q (N1) = -0.95
	q (H1) = 0.34

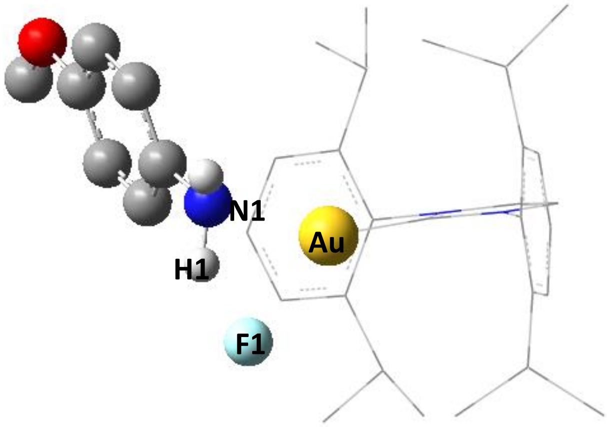

TS-5’

Au-F1 = 2.30 Å	q (Au) = 0.52
F1-H1 = 1.57 Å	q (N1) = -0.96
N1-Au = 2.28 Å	q (F1) = -0.74
N1-H1 = 1.05 Å	q (H1) = 0.48

Table S7. Optimised structures of transition states and intermediates of the reaction energy profile detailed in Figure S4. Selected bond lengths and angles are provided below each structure. Full reaction coordinates for each structure can be found under XYZ coordinates. Protons and IPr-ligand removed for clarity.

4.7 Uncatalyzed S_NAr Energy Barriers

The energy barrier to the S_NAr reaction of both 4-methoxyphenol, 4-methoxyaniline and their {Au(IPr)} bound analogues was computationally modelled, as shown in Figure S5.

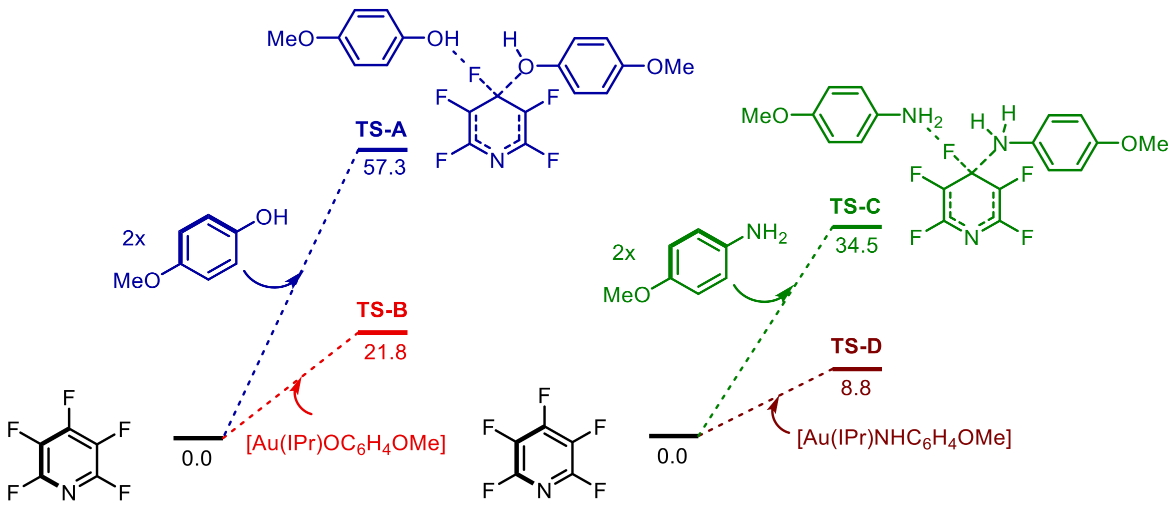

Figure S5. Computationally determined energy barrier differences for the S_NAr reactions of pentafluoropyridine with 4-methoxyphenol (TS-A), 4-methoxyaniline (TS-C), IPrAu(I) coordinated 4-methoxyphenol (TS-B) and IPrAu(I) coordinated 4-methoxyaniline (TS-D). Calculated using the PBE0 functional. Energy values reported in Kcal mol^{-1}.

4.8 Hammett Plots and Energy Profiles for Para-Substituent Variation.

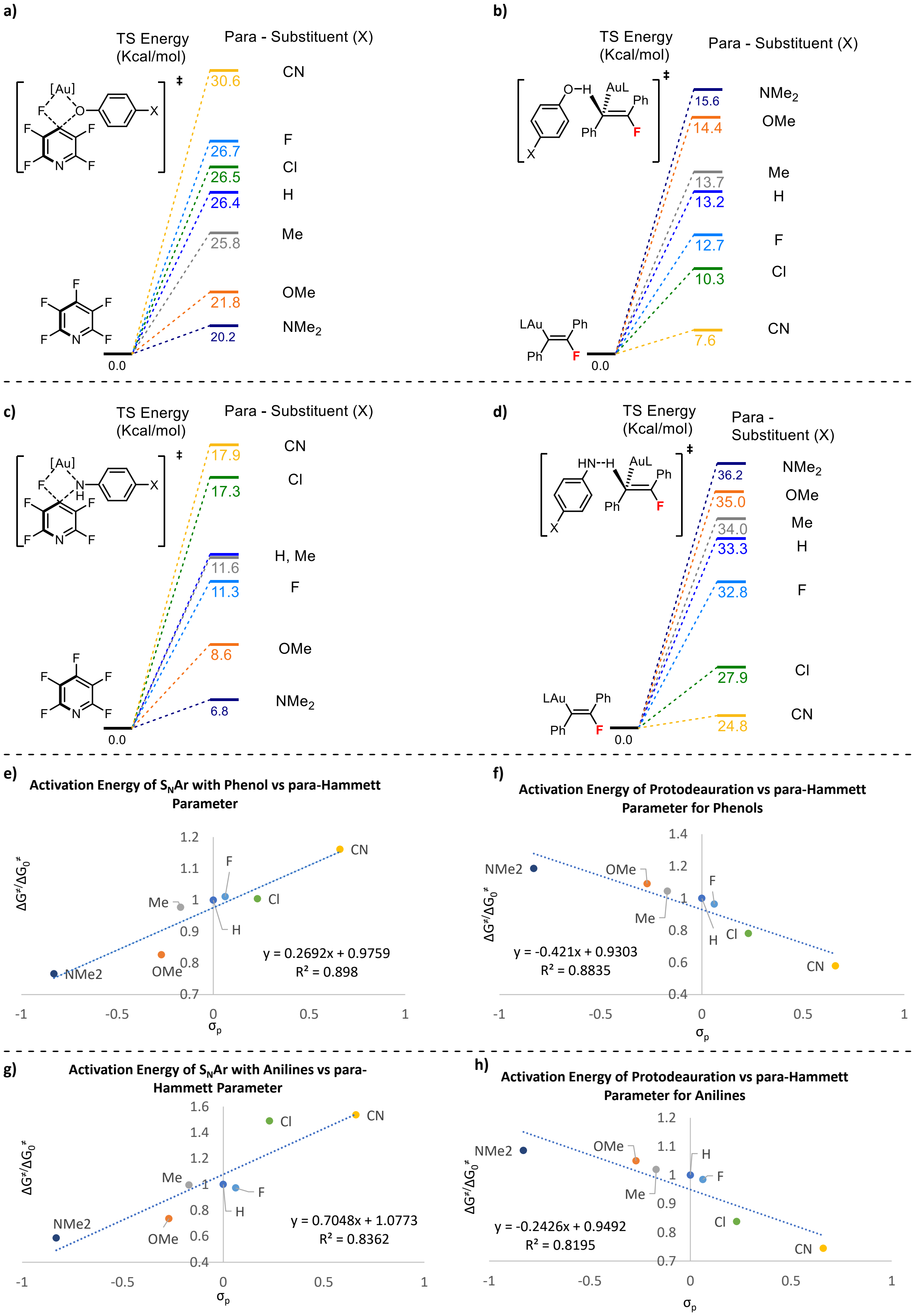

Figure S6. Computationally derived relative activation energies to S_NAr of a series of Au(I)-bound phenols (a), and anilines (c), with pentafluoropyridine; and the relative activation energies of protodeauration of **Int-4** with the same series of phenols (b) and anilines (d), and their associated Hammett plots (e-h). All energies are quoted in kcal mol^{-1}.

5. Kinetic Studies

Kinetic data was used to determine the catalyst and reagent order for both the Au(I) catalysed reactions of pentafluoropyridine, diphenylacetylene and 4-methoxyphenol, or 4-methoxyaniline. Catalyst order was determined by initial rates experiments, monitoring the initial consumption of 4-methoxyphenol or 4-methoxyaniline through ^{1}H NMR spectroscopy. Three non-zero time points were taken at 5 minutes, 10 minutes, and 15 minutes. Initial reaction rates were recorded for a range of catalyst concentrations, measured against an internal standard (mesitylene). Order in 4-methoxyphenol (or aniline) and pentafluoropyridine was determined by pseudo-first-order reactions.

Pseudo-first-order reactions were performed in-situ in a Bruker 500 MHz machine. ^{1}H NMR spectra were recorded with a scan range of 0 to 12 ppm. An FID was collected every 15 minutes over a period of 10 hours. The reactions were conducted at 120 °C. Conversions were calculated from reagent and product concentrations, based on proton integration value, relative to an internal standard (mesitylene). Reactions were performed in J. Young's NMR Tubes. In each case, a time-zero ^{1}H NMR spectrum was taken of the reaction mixture at room temperature, in the absence of pentafluoropyridine. The NMR tube was then taken into a glovebox and the reaction mixture was transferred into a PTFE liner and placed back in the NMR tube and re-submitted into the Bruker machine thermostated at 120 °C.

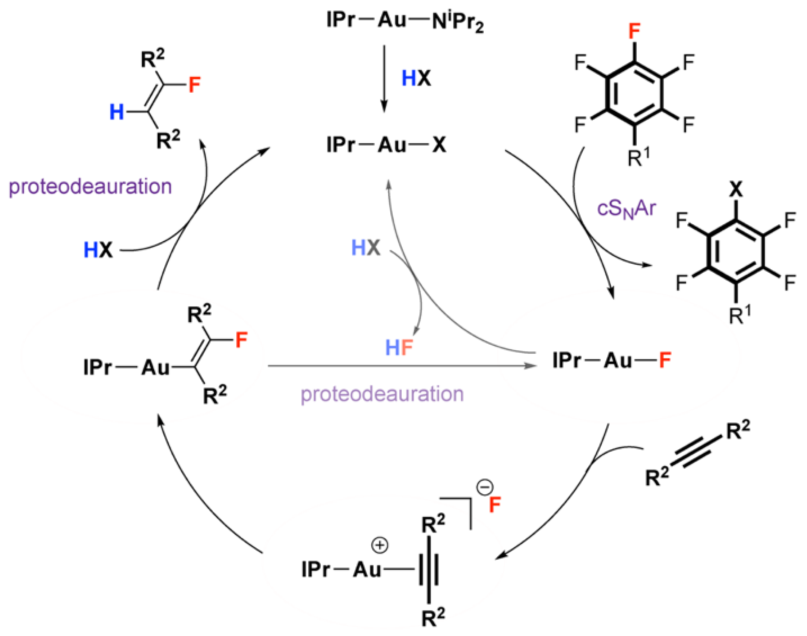

Figure S7. Plausible catalytic network for HF transfer.

As shown in Figure S7, the proposed reaction mechanism, which is supported by computational studies, suggests the non-trivial rate equations (1), (2). In the case of HX = 4-methoxyphenol, it is expected that $K_2 > K_1$ and the rate law can therefore be approximated as shown in equation (3). In this case, the order in reagent and catalyst can be determined through either consumption of **A**, or formation of **B** or **C**. In the case of HX = 4-methoxyaniline, $k_2 > k_1$ and the rate equation remains non-trivial. Rates data has been provided for both cases however rate orders have not been extracted for the case of HX = 4-methoxyaniline.

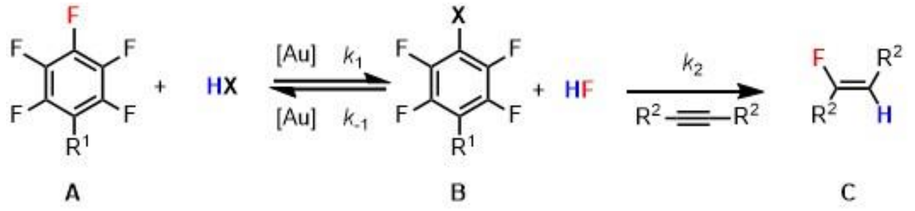

$$\frac{d[\boldsymbol{C}]}{dt} = k_2[Alkyne][HF][Au] \tag{1}$$

$$\frac{d[\boldsymbol{B}]}{dt} = k_1[A][HX][Au] - k_{-1}[B][HF][Au] - k_2[Alkyne][HF][Au] \tag{2}$$

$$for\ k_2 \gg k_1, k_{-1}, \frac{d[\boldsymbol{C}]}{dt} = \frac{d[\boldsymbol{B}]}{dt} = -\frac{d[\boldsymbol{A}]}{dt} = k'[A][HX][Au] \tag{3}$$

5.1 Catalyst Order for 4-Methoxyphenol Reaction

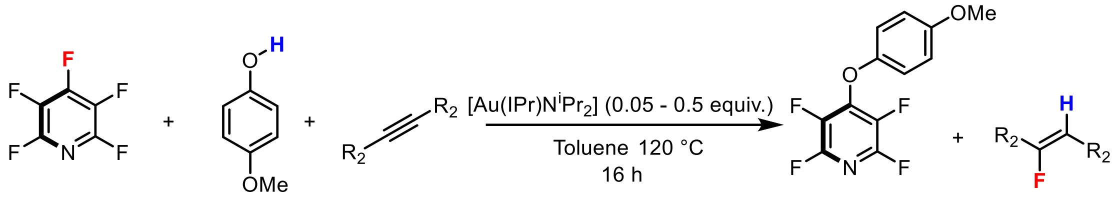

1 equiv.
[0.04 M]

1.2 equiv.

3 equiv.

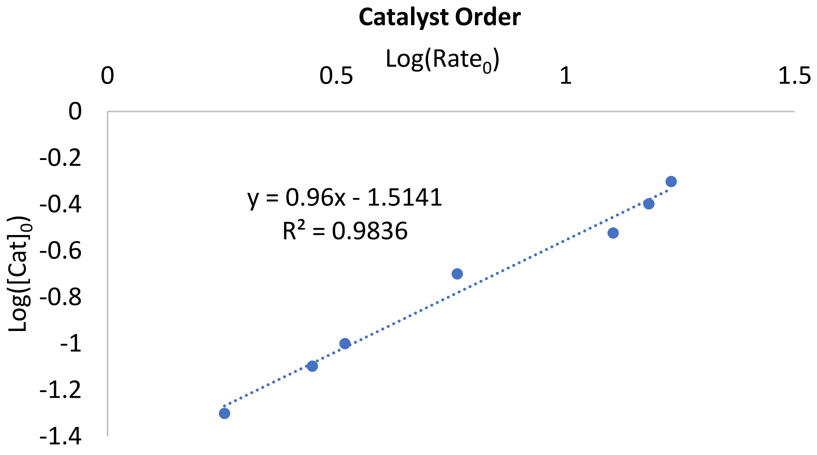

Figure S8. Log-log plot of initial rate ($Rate_0$), against total concentration ($[Cat]_0$) for a series of catalyst loadings: 0.05, 0.08, 0.1, 0.2, 0.3, 0.4, 0.5 equivalents, relative to pentafluoropyridine (0.04 M in 0.5 ml toluene). Conversions determined by ^1H NMR spectroscopy, through integrations of 4-methoxyphenol intensity relative to one equivalent of internal standard (mesitylene).

5.2 Catalyst Order for 4-Methoxyaniline Reaction

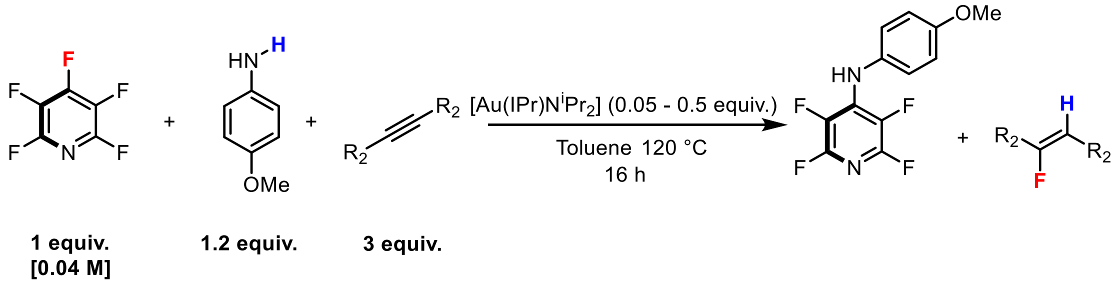

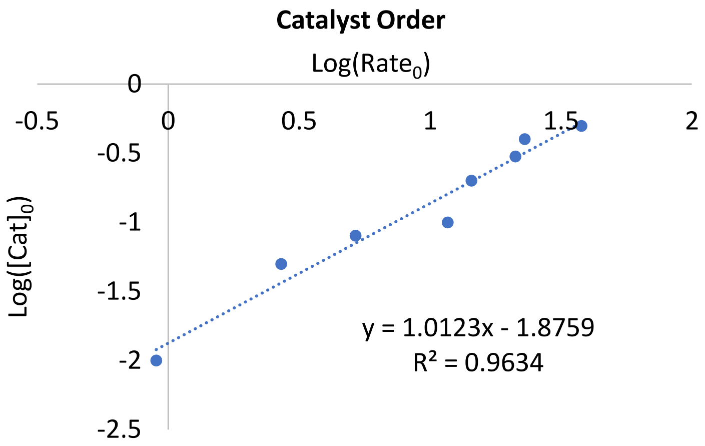

Figure S9. Log-log plot of initial rate ($Rate_0$), against total concentration ($[Cat]_0$) for a series of catalyst loadings: 0.05, 0.08, 0.1, 0.2, 0.3, 0.4, 0.5 equivalents, relative to pentafluoropyridine (0.04 M in 0.5 ml toluene). Conversions determined by ^{1}H NMR spectroscopy, through integrations of 4-methoxyphenol intensity relative to one equivalent of internal standard (mesitylene).

5.3 Pentafluoropyridine Order for 4-Methoxyphenol Reaction

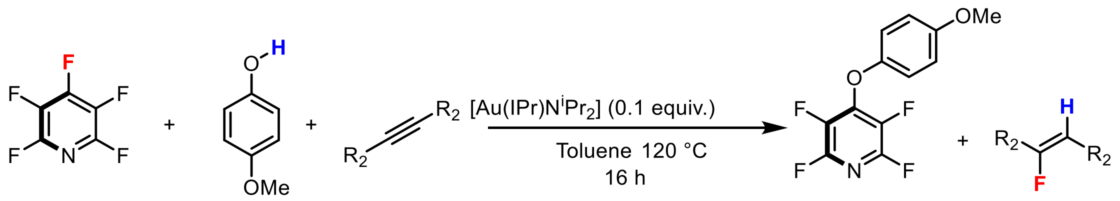

1 equiv. **[0.04 M]** **10 equiv.** **10 equiv.**

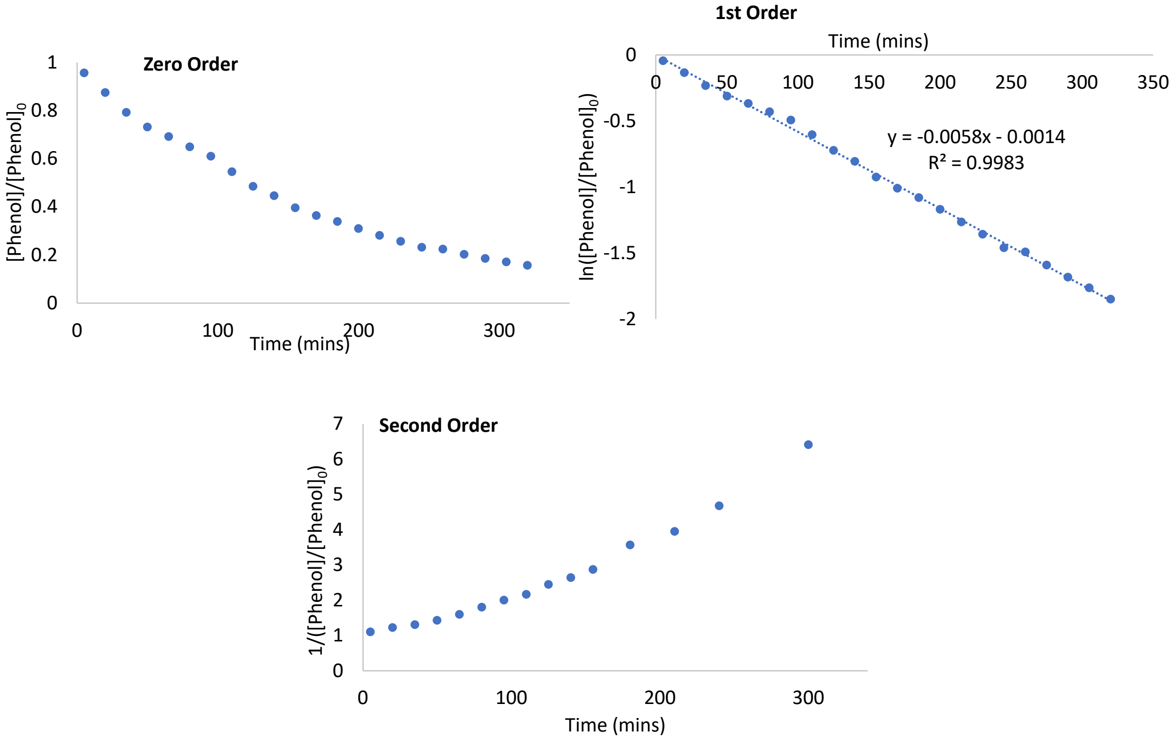

Figure S10. Determination of reaction order of pentafluorpyridine reagent through zero, first and second order plots of starting material (4-methoxyphenol) consumption over time. Reactions were performed with 0.1 : 1: 10 : 10 equivalents of catalyst : pentafluoropyridine : 4-methoxyphenol : diphenylacetylene.

5.4 4-Methoxyphenol Order for Reaction with 4-Methoxyphenol

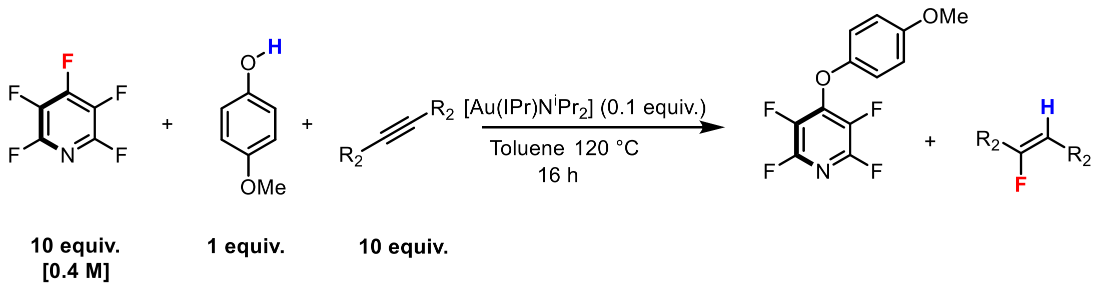

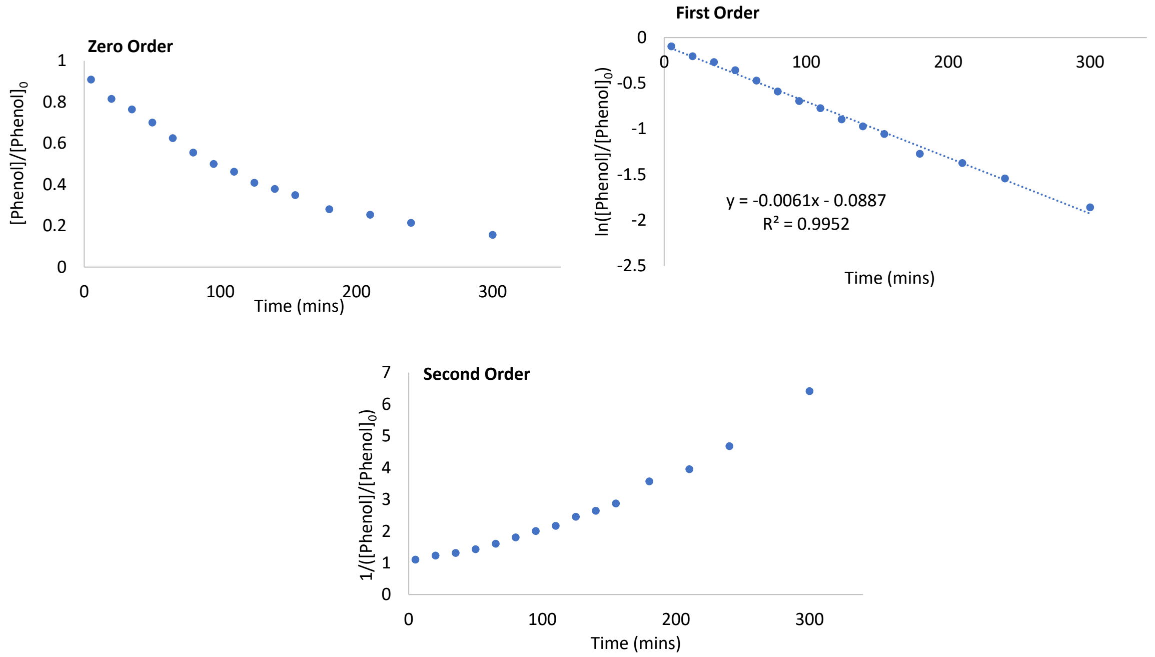

Figure S11. Determination of reaction order of 4-methoxyphenol reagent through zero, first and second order plots of starting material (4-methoxyphenol) consumption over time. Reactions were performed with 0.1 : 1: 10 : 10 equivalents of catalyst : 4-methoxyphenol : pentafluoropyridine: diphenylacetylene.

5.5 Pentafluoropyridine Order for Reaction with 4-Methoxyaniline

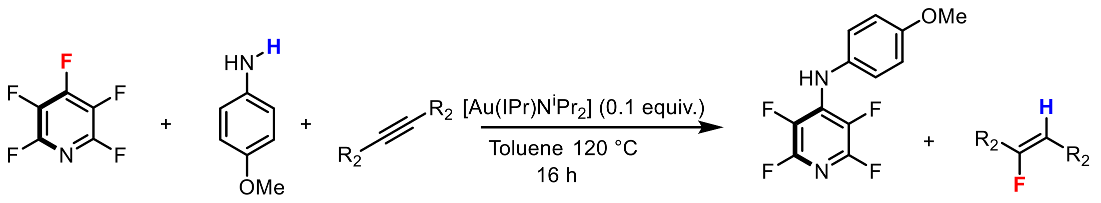

1 equiv.
[0.04 M]

10 equiv.

10 equiv.

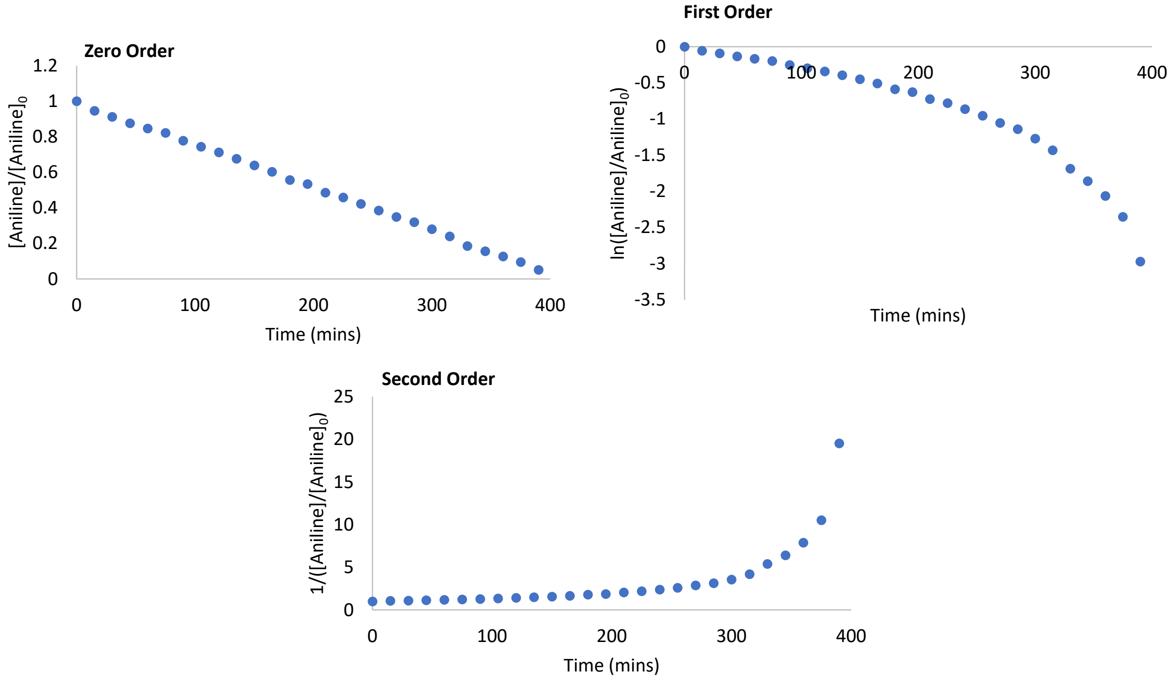

Figure S12. Determination of reaction order of pentafluorpyridine reagent through zero, first and second order plots of starting material (4-methoxyaniline) consumption over time. Reactions were performed with 0.1 : 1: 10 : 10 equivalents of catalyst : pentafluoropyridine : 4-methoxyaniline : diphenylacetylene.

5.6 4-Methoxyaniline Order for Reaction with 4-Methoxyaniline

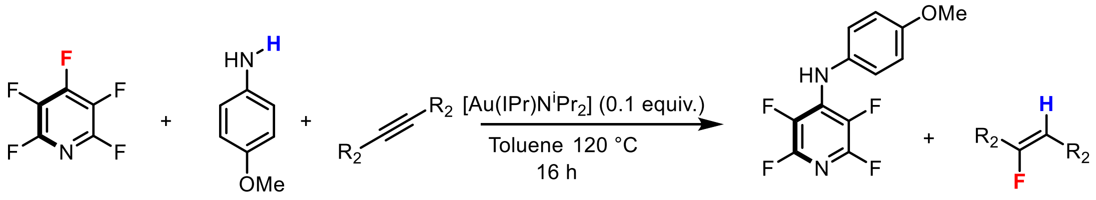

10 equiv.
[0.4 M]

1 equiv.

10 equiv.

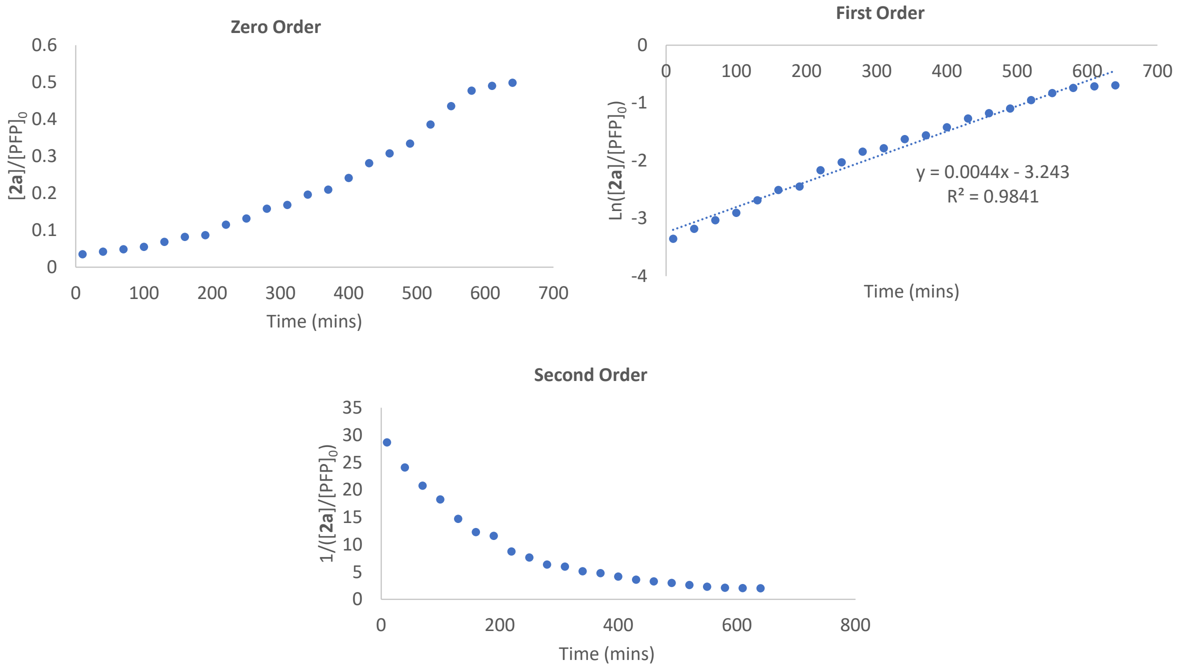

Figure S13. Determination of reaction order of 4-methoxyaniline reagent through zero, first and second order plots of starting material (4-methoxyaniline) consumption over time. Reactions were performed with 0.1 : 1: 10 : 10 equivalents of catalyst : 4-methoxyaniline : pentafluoropyridine : diphenylacetylene.

5.7 Product Formation Plots

The relative rates of fluoroarene (**1a**, **1h**), and fluoroalkene (**2a**) product formation are plotted over time for the Au(I) catalysed reaction of pentafluoropyridine with diphenylacetylene and either 4-methoxyphenol or 4-methoxyaniline.

A)

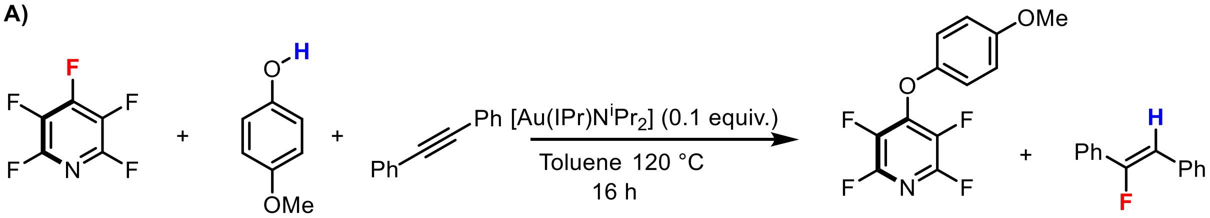

**1 equiv.** **[0.04 M]**	**1.2 equiv.**	**3 equiv.**	**1a**	**2a**

B)

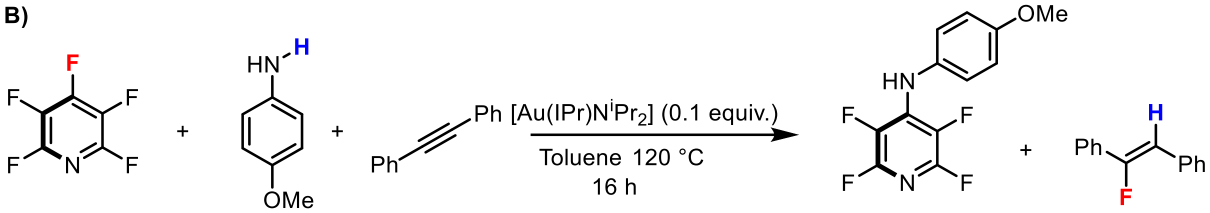

**1 equiv.** **[0.04 M]**	**1.2 equiv.**	**3 equiv.**	**1h**	**2a**

a)

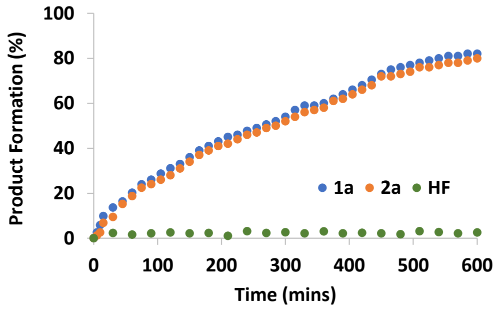

b)

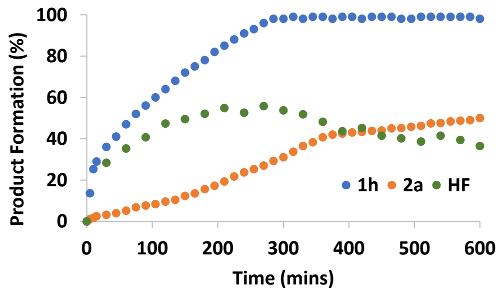

Figure S14. Plots of the formation of both products (**1a** and **2a**, or **1h** and **2a**), along with HF, for the reaction of pentafluoropyridine with diphenylacetylene and 4-methoxyphenol (A) or 4-methoxyaniline (B). Reactions were monitored by in-situ ^{1}H NMR kinetics at 120 °C. **1a** and **1d** product formations were monitored by the product methoxy environments at δ 3.21 and 3.30 ppm respectively. **2a** formation was monitored by the product doublet at δ 6.1 ppm. HF concentration was monitored by ^{19}F NMR environment at δ = 150.5 - 152.5 ppm.

5.8 Kinetic Isotope Effect: Competition Experiments

Deuterated analogues of 4-methoxyphenol and 4-methoxyaniline were synthesised by dissolving 50 mg of each nucleophile in 3 ml of hot D_2O. Recrystallization of the deuterated nucleophiles occurred by cooling the solution to 5 °C over 2 hours. The crystals were filtered, redissolved in dichloromethane and dried over activated 3Å mol sieves, before being freeze-pump-thawed. The reaction mixture was then filtered, and the solvent was removed in vacuo. The reactions were then set up in J. Young's NMR tubes as outlined in the general experimental. Deuterium incorporation was measured by ^{1}H NMR and high-resolution mass spectrometry and was shown to be >95 % in each case.

Kinetic isotope effects were determined for the Au(I) catalysed reactions of pentafluoropyridine with diphenylacetylene and either 4-methoxyphenol or 4-methoxyaniline, via competition experiments with an excess of 4-methoxyphenol or 4-methoxyaniline and their deuterated analogues, as shown in Figure S15. The ratio of protonated : deuterated fluoroalkene product (P_H/P_D) is provided in each case.

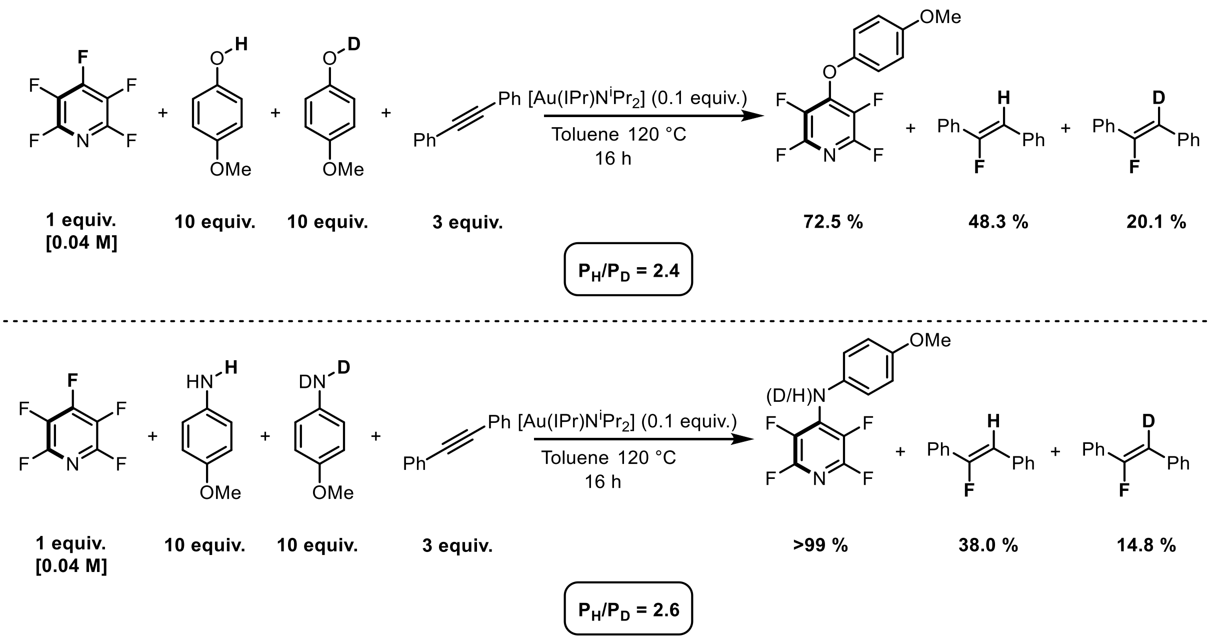

Figure S15. Ratio of protonated : deuterated products for the competition reactions of pentafluoropyridine with diphenylacetylene and a combination of either 4-methoxyphenol and d_1-4-methoxyphenol or 4-methoxyaniline and d_2-4-methoxyaniline.

5.9 Kinetic Isotope Effect: Rates Experiments

In addition to competition reactions, rates of fluoroalkene formation with protonated or deuterated phenols and anilines was determined. Rates (k_H and k_D) were abstracted under pseudo-first order plots of either 4-methoxyphenol consumption (Figure S16, k_H/k_D = 1.1 ± 0.1), or **2a** production (figure S17, k_H/k_D = 2.8 ± 0.3).

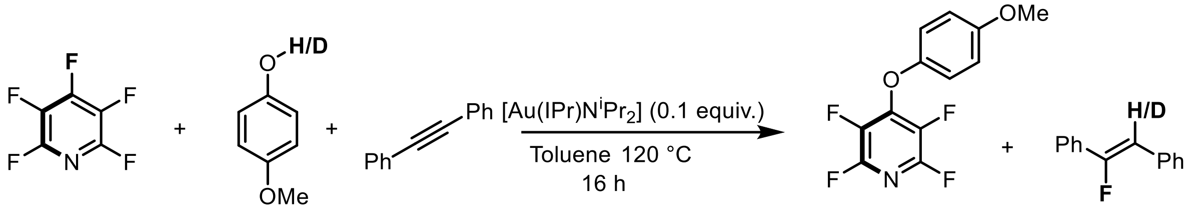

10 equiv. [0.4 M] **1.0 equiv.** **10 equiv.** **1a** **2a**

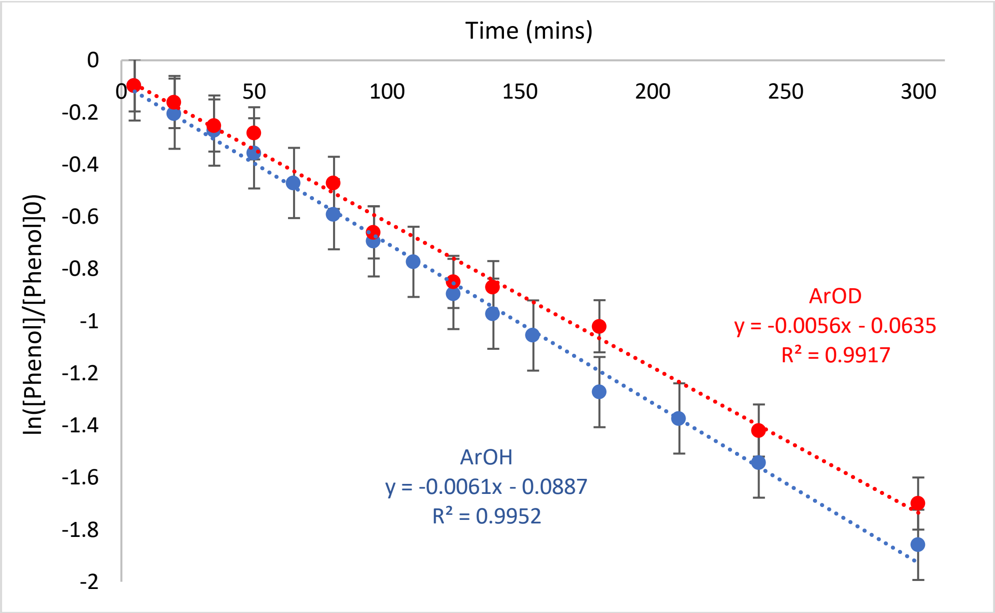

Figure S16. Pseudo-first order rate plots for the consumption of 4-methoxyphenol or d_1-4-methoxyphenol.

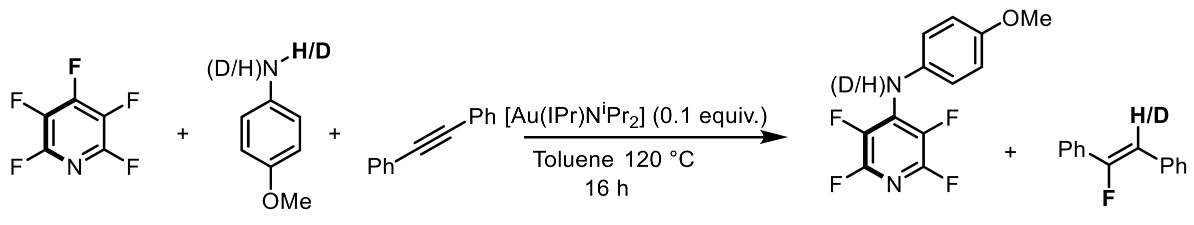

10 equiv. [0.4 M] **1.0 equiv.** **10 equiv.** **1h** **2a**

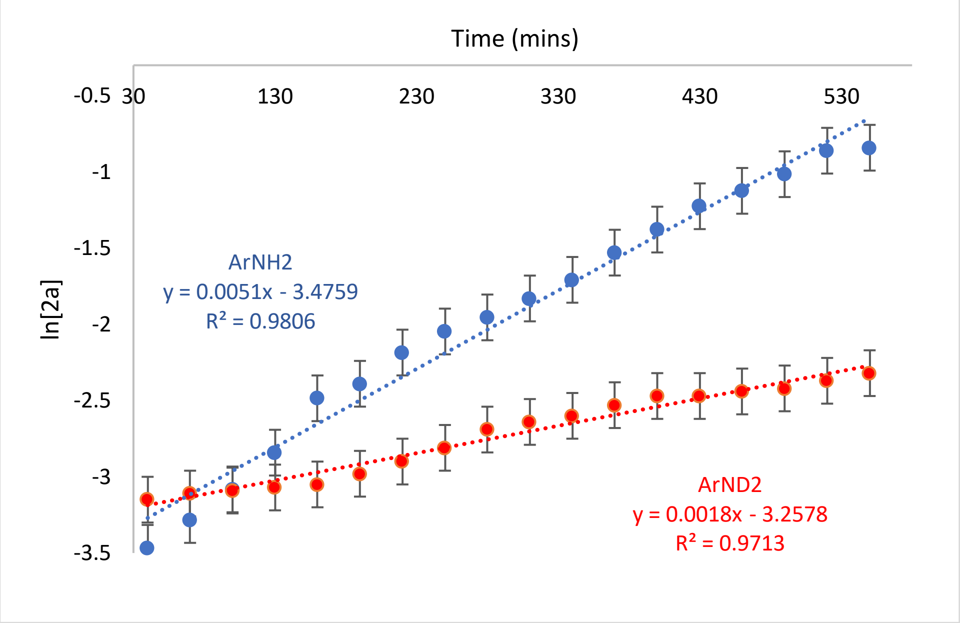

Figure S17. Pseudo-first order rate plots for the production of product **2a** in the presence of either 4-methoxyaniline or d_1-4-methoxyaniline.

6. References

[S1] Mio, M. J.; Kopel, L. C.; Braun, J. B.; Gadzikwa, T. L.; Hull, K. L.; Brisbois, R. G.; Markworth, C. J.; Grieco, P. A. One-Pot Synthesis of Symmetrical and Unsymmetrical Bisarylethynes by a Modification of the Sonogashira Coupling Reaction. *Org. Lett.* **2002**, *4*, 3199–3202.

[S2] Semba, K.; Fujihara, T.; Xu, T.; Terao, J.; Tsuji, Y. Copper-Catalyzed Highly Selective Semihydrogenation of Non-Polar Carbon-Carbon Multiple Bonds Using a Silane and an Alcohol. *Adv. Synth. Catal.,* **2012**, *354*, 1542–1550.

[S3] Hu, J.-Y.; Zhang, J.; Wang, G.-X.; Sun, H.-L.; Zhang, J.-L. Constructing a Catalytic Cycle for C–F to C–X (X = O, S, N) Bond Transformation Based on Gold-Mediated Ligand Nucleophilic Attack. *Inorg. Chem.* **2016**, *55*, 2274–2283.

[S4] Johnson, M. W.; Shevick, S. L.; Toste, F. D.; Bergman, R. G. Preparation and Reactivity of Terminal Gold(i) Amides and Phosphides. *Chem. Sci.* **2013**, *4*, 1023–1027.

[S5] Tsui, E. Y.; Müller, P.; Sadighi, J. P. Reactions of a Stable Monomeric Gold(I) Hydride Complex. *Angew. Chem. Int. Ed.* **2008**, *47*, 8937–8940.

[S6] Collado, A.; Gómez-Suárez, A.; Martin, A. R.; Slawin, A. M. Z.; Nolan, S. P. Straightforward Synthesis of [Au(NHC)X] (NHC = N-Heterocyclic Carbene, X = Cl, Br, I) Complexes. *Chem. Commun.* **2013**, *49*, 5541–5543.

[S7] Akana, J. A.; Bhattacharyya, K. X.; Müller, P.; Sadighi, J. P. Reversible C−F Bond Formation and the Au-Catalyzed Hydrofluorination of Alkynes. *J. Am. Chem. Soc.* **2007**, *129*, 7736–7737.

[S8] Brittain, W. D. G.; Cobb, S. L. Tetrafluoropyridyl (TFP): A General Phenol Protecting Group Readily Cleaved under Mild Conditions. *Org. Biomol. Chem.* **2019**, *17*, 2110–2115.

[S9] Poorfreidoni, A.; Ranjbar-Karimi, R. Synthesis of Substituted Imidazopyridines from Perfluorinated Pyridine Derivatives. *Tet. Lett.* **2016**, *57*, 5781–5783.

[S10] Christopher, J. A.; Brophy, L.; Lynn, S. M.; Miller, D. D.; Sloan, L. A.; Sandford, G. Synthetic Utility of 4-Bromo-2,3,5,6-Tetrafluoropyridine. *J. Fluor. Chem.* **2008**, *129*, 447–454.

[S11] Ranjbar-Karimi, R.; Poorfreidoni, A.; Masoodi, H. R. Survey Reactivity of Some N-Aryl Formamides with Pentafluoropyridine. *J. Fluor. Chem.* **2015**, *180*, 222–226.

[S12] Zhang, J.; Wu, J.; Xiong, Y.; Cao, S. Synthesis of Unsymmetrical Biaryl Ethers through Nickel-Promoted Coupling of Polyfluoroarenes with Arylboronic Acids and Oxygen. *Chem. Commun.* **2012**, *48*, 8553–8555.

[S13] Gauthier, R.; Mamone, M.; Paquin, J.-F. Gold-Catalyzed Hydrofluorination of Internal Alkynes Using Aqueous HF. *Org. Lett.* **2019**, *21*, 9024–9027.

[S14] Wenz, J.; Rettenmeier, C. A.; Wadepohl, H.; Gade, L. H. Catalytic C–F Bond Activation of Geminal Difluorocyclopropanes by Nickel(i) Complexes via a Radical Mechanism. *Chem. Commun.* **2016**, *52*, 202–205.

[S15] Zhao, Y.; Jiang, F.; Hu, J. Spontaneous Resolution of Julia-Kocienski Intermediates Facilitates Phase Separation to Produce Z- and E-Monofluoroalkenes. *J. Am. Chem. Soc.* **2015**, *137*, 5199–5203.

[S16] Engman, M.; Diesen, J. S.; Paptchikhine, A.; Andersson, P. G. Iridium-Catalyzed Asymmetric Hydrogenation of Fluorinated Olefins Using N,P-Ligands: A Struggle with Hydrogenolysis and Selectivity. *J. Am. Chem. Soc.* **2007**, *129*, 4536–4537.

[S17] O'Connor, T. J.; Toste, F. D. Gold-Catalyzed Hydrofluorination of Electron-Deficient Alkynes: Stereoselective Synthesis of β-Fluoro Michael Acceptors. *ACS Catal.* **2018**, *8*, 5947–5951.

7. XYZ Coordinates

TS-1

C	-3.411979	1.058384	3.757989
C	-2.105930	1.031243	3.265117
C	-1.945945	1.090050	1.873715
C	-3.018834	1.170968	0.976152
C	-4.303506	1.205271	1.521199
C	-4.498689	1.149142	2.895980
H	-3.578514	1.003324	4.829614
H	-5.161826	1.257886	0.858370
H	-5.506839	1.169159	3.299745
C	0.220572	2.086787	1.094790
C	1.385544	1.567585	0.632578
H	-0.090514	3.104300	1.268472
H	2.303554	2.037736	0.318528
C	2.218958	-0.757898	0.209405
C	2.349440	-1.042094	-1.157701
C	2.982229	-1.380563	1.205221
C	3.306569	-1.989350	-1.521323
C	3.927278	-2.319668	0.786584
C	4.088393	-2.619812	-0.559465
H	3.436272	-2.244631	-2.568065
H	4.538239	-2.825725	1.528037
H	4.825742	-3.356959	-0.863270
C	1.440300	-0.406783	-2.191079
C	0.257027	-1.334458	-2.488698
C	2.175628	-0.024678	-3.474601
H	1.034399	0.516725	-1.762859
H	-0.304205	-1.567658	-1.578296
H	-0.427499	-0.866393	-3.204096
H	0.607639	-2.279153	-2.917732
H	3.044661	0.607030	-3.267369
H	2.521310	-0.906766	-4.022936
H	1.503022	0.525290	-4.139845
C	2.813783	-1.068499	2.678029
C	4.058298	-0.369548	3.230453
C	2.472174	-2.324792	3.481769
H	1.972452	-0.376504	2.786296
H	4.275951	0.551721	2.680730
H	3.914707	-0.113678	4.285132
H	4.938373	-1.017561	3.160469
H	1.585365	-2.827914	3.085311
H	3.300163	-3.041435	3.470657
H	2.278800	-2.059394	4.526214
C	-2.820419	1.172594	-0.525823
C	-3.517071	2.361219	-1.189537
C	-3.285397	-0.155793	-1.128940
H	-1.747820	1.267242	-0.727446
H	-3.182414	3.311008	-0.761284
H	-3.300430	2.373558	-2.262291
H	-4.604369	2.305420	-1.075060
H	-2.734239	-0.995559	-0.695304
H	-4.350321	-0.325773	-0.939706
H	-3.123734	-0.159297	-2.211885
C	-0.928283	0.912963	4.211046
C	-0.898678	-0.466089	4.873593
C	-0.929070	2.033806	5.252001
H	-0.009354	1.015255	3.624009
H	-0.849933	-1.265752	4.129083
H	-0.026176	-0.554070	5.529272
H	-1.795032	-0.631912	5.480115
H	-0.938214	3.019972	4.777622
H	-1.801422	1.969503	5.910312
H	-0.036294	1.964115	5.881194
Au	-0.752780	-1.945821	1.289928
C	-3.604307	-3.423678	1.045508
C	-4.261382	-4.251240	0.150162
C	-4.261303	-2.368556	1.669352
C	-5.607695	-4.030011	-0.131951
H	-3.716971	-5.062096	-0.321750
C	-5.597585	-2.142322	1.383940
H	-3.724134	-1.732544	2.365399
C	-6.279423	-2.970851	0.484643
H	-6.114495	-4.683468	-0.831895
H	-6.135218	-1.323567	1.849848
O	-7.582256	-2.663978	0.274266
C	-8.313647	-3.486035	-0.609408
H	-9.327218	-3.084465	-0.629079
H	-7.895916	-3.459981	-1.623639
H	-8.344200	-4.525406	-0.260144
C	-2.396277	-4.532935	3.523011
C	-2.729655	-5.651281	4.243910
C	-2.334855	-7.012365	2.525030
C	-1.966380	-5.979075	1.698669
N	-2.708913	-6.887821	3.783692
F	-2.346237	-8.249606	2.016503
F	-1.646936	-6.191446	0.404429
F	-2.465100	-3.296035	4.064240
F	-3.136785	-5.488966	5.507848
F	-0.328019	-4.126802	2.189111
C	-1.886042	-4.653388	2.201671
O	-2.241855	-3.612256	1.271622
C	-0.003137	-0.153947	1.050895
N	-0.616586	1.015188	1.343705
N	1.225537	0.194071	0.610386

[Au(IPr)F]

C	-0.294316	-1.255825	-1.759882
C	-1.283806	-0.329320	-1.739999
H	0.777233	-1.161550	-1.687970
H	-1.258202	0.744463	-1.647578

C	-0.232156	-3.744931	-1.963704
C	0.014715	-4.426907	-0.765051
C	0.126352	-4.245243	-3.222093
C	0.662192	-5.660039	-0.853971
C	0.772166	-5.482155	-3.256693
C	1.038893	-6.181398	-2.085894
H	0.863565	-6.223312	0.052417
H	1.059757	-5.906710	-4.214064
H	1.538155	-7.144686	-2.134358
C	-3.775105	-0.426236	-1.900169
C	-4.309600	-0.061316	-3.142772
C	-4.460863	-0.263440	-0.689462
C	-5.585927	0.503359	-3.147535
C	-5.733930	0.305573	-0.748462
C	-6.289858	0.687106	-1.963553
H	-6.037573	0.792305	-4.091964
H	-6.300547	0.440585	0.168146
H	-7.283757	1.124206	-1.988830
N	-0.910214	-2.485746	-1.900295
N	-2.473539	-1.021898	-1.869342
C	-0.201800	-3.511359	-4.505898
H	-0.627643	-2.537813	-4.241030
C	-1.263191	-4.272748	-5.304332
H	-0.890013	-5.253412	-5.618338
H	-2.166603	-4.430179	-4.706552
H	-1.537904	-3.712040	-6.203971
C	1.050158	-3.250676	-5.344662
H	1.800234	-2.689882	-4.778395
H	1.511633	-4.186063	-5.677669
H	0.793427	-2.673344	-6.238514
C	-3.570110	-0.306387	-4.441879
H	-2.562261	-0.658250	-4.197530
C	-3.420953	0.975339	-5.262391
H	-4.393940	1.365526	-5.578363
H	-2.915128	1.759163	-4.690149
H	-2.835754	0.779502	-6.166457
C	-4.257475	-1.411686	-5.248208
H	-4.336047	-2.333803	-4.663421
H	-5.268641	-1.110927	-5.542657
H	-3.690106	-1.627818	-6.159604
C	-0.442296	-3.889687	0.575565
H	-0.810358	-2.869311	0.424708
C	0.704957	-3.817114	1.583742
H	0.356545	-3.371426	2.520791
H	1.097251	-4.811805	1.819124
H	1.533112	-3.211119	1.203437
C	-1.609158	-4.722876	1.112833
H	-2.436849	-4.746980	0.396884
H	-1.297057	-5.755343	1.303148
H	-1.977952	-4.301580	2.053985
C	-3.883901	-0.726486	0.632302
H	-2.847695	-1.036522	0.460747
C	-4.648146	-1.949033	1.147610
H	-4.636752	-2.758127	0.410341
H	-4.196724	-2.319025	2.074169
H	-5.693317	-1.697297	1.356996
C	-3.859649	0.397138	1.669139
H	-3.300710	1.264683	1.304888
H	-4.870751	0.731827	1.922896
H	-3.387241	0.048638	2.592988
C	-2.256481	-2.354964	-1.969608
Au	-3.588018	-3.771299	-2.182143
F	-4.933629	-5.198711	-2.405068

Int-1

C	-0.503675	-2.174040	-1.433482
C	-1.202452	-1.012229	-1.438358
H	0.535619	-2.383141	-1.236760
H	-0.903320	0.004850	-1.242728
C	-1.078515	-4.570349	-1.850150
C	-0.966885	-5.310433	-0.662362
C	-0.896615	-5.122022	-3.124185
C	-0.657531	-6.664118	-0.786395
C	-0.593624	-6.484314	-3.191494
C	-0.473839	-7.245069	-2.036891
H	-0.567263	-7.273243	0.106412
H	-0.444489	-6.949261	-4.161607
H	-0.232416	-8.301947	-2.110209
C	-3.591272	-0.414103	-1.892015
C	-3.767609	0.237858	-3.120515
C	-4.443320	-0.238066	-0.794228
C	-4.852929	1.107741	-3.229431
C	-5.513278	0.644518	-0.955637
C	-5.716086	1.309429	-2.158204
H	-5.031219	1.625541	-4.166781
H	-6.199662	0.804835	-0.129395
H	-6.557781	1.987472	-2.264505
N	-1.401194	-3.177604	-1.750722
N	-2.504491	-1.338914	-1.767925
C	-0.994564	-4.299100	-4.392661
H	-1.282767	-3.279449	-4.118263
C	-2.078045	-4.837412	-5.328081
H	-1.843364	-5.849961	-5.672621
H	-3.049908	-4.865312	-4.825411
H	-2.167749	-4.196129	-6.210810
C	0.363078	-4.219630	-5.094948
H	1.128122	-3.799469	-4.434611
H	0.703462	-5.210327	-5.414139
H	0.294424	-3.586921	-5.985736
C	-2.864317	-0.035046	-4.306684
H	-1.961485	-0.533744	-3.937142

C	-2.417639	1.247093	-5.008529
H	-3.259165	1.767042	-5.477379
H	-1.940088	1.940652	-4.309789
H	-1.700267	1.010382	-5.800404
C	-3.550975	-0.993927	-5.284604
H	-3.833696	-1.928632	-4.789400
H	-4.461299	-0.542995	-5.693851
H	-2.883595	-1.232704	-6.119665
C	-1.224483	-4.676167	0.690908
H	-0.911393	-3.626698	0.633291
C	-0.426206	-5.326032	1.818888
H	-0.544575	-4.743223	2.737177
H	-0.785396	-6.337817	2.033695
H	0.641447	-5.382561	1.584481
C	-2.721714	-4.700468	1.014673
H	-3.316083	-4.212545	0.237599
H	-3.080680	-5.729631	1.099955
H	-2.912675	-4.190092	1.965014
C	-4.244813	-0.971624	0.516498
H	-3.349482	-1.595466	0.427191
C	-5.421725	-1.903571	0.813300
H	-5.567589	-2.634902	0.012360
H	-5.242671	-2.454298	1.742201
H	-6.353554	-1.340281	0.930552
C	-4.005604	0.009627	1.666100
H	-3.146451	0.657522	1.465732
H	-4.877885	0.650792	1.831013
H	-3.814299	-0.535925	2.595606
C	-2.637461	-2.670347	-1.964077
Au	-4.278097	-3.620996	-2.450267
F	-5.979935	-4.588007	-2.923842
C	-4.008984	-8.337666	0.674156
C	-4.025249	-7.942047	-0.662179
C	-4.897532	-6.940112	-1.086331
C	-5.762193	-6.313010	-0.185245
C	-5.743304	-6.720961	1.154759
C	-4.880509	-7.721277	1.576881
H	-3.359276	-8.393657	-1.388487
H	-4.909476	-6.639503	-2.129431
H	-6.417350	-6.240048	1.857046
H	-4.864920	-8.041333	2.614366
O	-6.616879	-5.334606	-0.546871
H	-6.453027	-5.084567	-1.491286
O	-3.189001	-9.302970	1.190444
C	-2.430954	-10.052154	0.273323
H	-1.697856	-9.430795	-0.258021
H	-1.899817	-10.807292	0.855252
H	-3.068848	-10.550115	-0.468953

TS-2

C	-0.306634	-1.608979	-2.585283
C	-1.355055	-0.756713	-2.495486
H	0.739156	-1.435783	-2.782107
H	-1.414940	0.315420	-2.593549
C	-0.018639	-4.079901	-2.388461
C	0.619246	-4.480328	-1.202656
C	0.083920	-4.792930	-3.592863
C	1.375157	-5.652936	-1.251782
C	0.844196	-5.963676	-3.580793
C	1.480991	-6.391137	-2.423537
H	1.881385	-5.995234	-0.354457
H	0.934441	-6.548987	-4.491460
H	2.064570	-7.307252	-2.434531
C	-3.796382	-1.008848	-2.041789
C	-4.616299	-0.848768	-3.170159
C	-4.211639	-0.683647	-0.740557
C	-5.897735	-0.335562	-2.963904
C	-5.502174	-0.170282	-0.593976
C	-6.336639	0.002264	-1.690190
H	-6.562008	-0.205543	-3.813598
H	-5.856776	0.091352	0.398718
H	-7.338037	0.399952	-1.550996
N	-0.807720	-2.880616	-2.371575
N	-2.470693	-1.528492	-2.228744
C	-0.582777	-4.334705	-4.876713
H	-1.077661	-3.377795	-4.679944
C	-1.664579	-5.319770	-5.327080
H	-1.242294	-6.309885	-5.529313
H	-2.432477	-5.430273	-4.555786
H	-2.143740	-4.963551	-6.245769
C	0.449147	-4.096977	-5.983549
H	1.211837	-3.375746	-5.673311
H	0.961705	-5.024407	-6.260084
H	-0.040314	-3.709705	-6.883314
C	-4.166669	-1.224400	-4.569649
H	-3.128035	-1.567030	-4.512794
C	-4.200831	-0.018712	-5.513293
H	-5.220241	0.359707	-5.643323
H	-3.586668	0.804763	-5.135279
H	-3.824102	-0.298243	-6.502729
C	-4.998621	-2.386423	-5.120508
H	-4.933906	-3.260212	-4.465590
H	-6.055574	-2.113525	-5.209171
H	-4.640959	-2.671934	-6.115954
C	0.551397	-3.662446	0.074158
H	-0.276736	-2.952105	-0.024992
C	1.843370	-2.853539	0.247525
H	1.786880	-2.230028	1.145846
H	2.706674	-3.519665	0.354353

H	2.034614	-2.198330	-0.608811
C	0.269725	-4.510165	1.315999
H	-0.663361	-5.074489	1.229274
H	1.086279	-5.211625	1.520728
H	0.183684	-3.857596	2.191184
C	-3.321267	-0.852055	0.475965
H	-2.401560	-1.353291	0.156182
C	-3.971942	-1.739404	1.541494
H	-4.222138	-2.730594	1.146967
H	-3.282330	-1.869664	2.382617
H	-4.883284	-1.278047	1.939259
C	-2.927537	0.514593	1.047579
H	-2.422606	1.137599	0.301505
H	-3.807875	1.064065	1.398708
H	-2.252437	0.389325	1.900393
C	-2.145872	-2.843681	-2.151937
Au	-3.399086	-4.329413	-1.720709
C	-7.188892	-5.404643	-3.599400
C	-6.438468	-5.251956	-2.421953
C	-6.941229	-4.476643	-1.364009
C	-8.183712	-3.868930	-1.496328
C	-8.928914	-4.021675	-2.664150
C	-8.429462	-4.791015	-3.713891
H	-6.793200	-6.007449	-4.411184
H	-6.327319	-4.377842	-0.470819
H	-8.573190	-3.270245	-0.677939
H	-9.900243	-3.543849	-2.757062
H	-9.009231	-4.915393	-4.624006
C	-5.168767	-5.904815	-2.310145
C	-4.172586	-6.625999	-2.271899
C	-3.144008	-7.619792	-2.279128
C	-2.930707	-8.375465	-3.444472
C	-2.371278	-7.870283	-1.133315
C	-1.960877	-9.368689	-3.461547
H	-3.534573	-8.177631	-4.324789
C	-1.403153	-8.866844	-1.168040
H	-2.537146	-7.292708	-0.225590
C	-1.194073	-9.615917	-2.323986
H	-1.805664	-9.953998	-4.363414
H	-0.811720	-9.060766	-0.278078
H	-0.437956	-10.395989	-2.338340
F	-4.472318	-4.709224	0.284452
C	-4.361955	-7.478704	5.126502
C	-5.165207	-6.701859	4.293126
C	-4.659381	-6.208894	3.090343
C	-3.346008	-6.482520	2.700403
C	-2.545957	-7.263952	3.545277
C	-3.047633	-7.754533	4.740397
H	-6.190334	-6.470536	4.562848
H	-5.278451	-5.605325	2.433285
H	-1.523482	-7.477267	3.246632
H	-2.432149	-8.360617	5.398603
O	-2.824383	-6.022808	1.546638
H	-3.514969	-5.462412	1.046271
O	-4.759507	-8.009767	6.323485
C	-6.077332	-7.755388	6.729173
H	-6.811811	-8.155742	6.015888
H	-6.207216	-8.256797	7.690500
H	-6.267692	-6.680430	6.859286

Int-2

C	2.883958	2.791914	-1.201028
C	1.821588	3.627723	-1.117867
H	3.937259	2.986136	-1.323534
H	1.754133	4.703298	-1.150243
C	3.197926	0.320233	-1.115648
C	3.830281	-0.077069	0.074592
C	3.333664	-0.377228	-2.326279
C	4.619115	-1.227863	0.021748
C	4.128987	-1.524560	-2.317504
C	4.763486	-1.947366	-1.157259
H	5.122221	-1.566317	0.922308
H	4.251399	-2.093425	-3.234811
H	5.376423	-2.844139	-1.172116
C	-0.634667	3.338900	-0.814003
C	-1.452038	3.414491	-1.953394
C	-1.053583	3.753792	0.460392
C	-2.738364	3.929523	-1.784767
C	-2.348557	4.265214	0.567849
C	-3.182883	4.351280	-0.538510
H	-3.399445	4.000628	-2.643775
H	-2.706848	4.594288	1.538716
H	-4.187756	4.749584	-0.429231
N	2.386964	1.506273	-1.093422
N	0.702683	2.832056	-0.960616
C	2.683013	0.080864	-3.618231
H	2.075013	0.964410	-3.398486
C	1.740987	-0.982969	-4.186775
H	2.278543	-1.902248	-4.442424
H	0.960744	-1.235880	-3.463004
H	1.260602	-0.613252	-5.099283
C	3.742493	0.491948	-4.645963
H	4.395885	1.278879	-4.256012
H	4.375442	-0.357139	-4.925339
H	3.265783	0.865891	-5.558203
C	-0.983720	2.989009	-3.332550
H	0.011663	2.544281	-3.231953
C	-0.858689	4.199505	-4.263624
H	-1.829274	4.683965	-4.415398
H	-0.173398	4.949724	-3.856406
H	-0.482419	3.891563	-5.244933

C	-1.895897	1.918032	-3.936002
H	-1.967114	1.046682	-3.278354
H	-2.909313	2.298177	-4.102532
H	-1.502636	1.588842	-4.903914
C	3.723260	0.718191	1.363381
H	2.889804	1.421472	1.258675
C	5.003234	1.534621	1.584280
H	4.916329	2.143068	2.490363
H	5.868715	0.873802	1.705401
H	5.214757	2.204987	0.744527
C	3.419113	-0.156150	2.581433
H	2.486033	-0.714717	2.460939
H	4.232241	-0.861428	2.786739
H	3.315296	0.478196	3.467971
C	-0.158028	3.695465	1.683854
H	0.743071	3.132769	1.417397
C	-0.818237	2.950314	2.846586
H	-1.076056	1.927843	2.549242
H	-0.123700	2.904494	3.692643
H	-1.718675	3.468511	3.196184
C	0.276433	5.107704	2.093417
H	0.791148	5.631637	1.280411
H	-0.587962	5.715402	2.383060
H	0.954566	5.062890	2.951858
C	1.040008	1.520334	-0.948423
Au	-0.226890	-0.037200	-0.799493
C	-4.191042	-1.209790	-2.030855
C	-3.299711	-0.877638	-0.998047
C	-3.694613	-0.004026	0.027434
C	-4.981143	0.521481	0.007169
C	-5.869283	0.191193	-1.014766
C	-5.472756	-0.675488	-2.032277
H	-3.872702	-1.886648	-2.817996
H	-2.966848	0.226471	0.807387
H	-5.292119	1.196086	0.799674
H	-6.873072	0.607067	-1.019459
H	-6.164036	-0.936778	-2.828262
C	-1.984343	-1.453533	-0.999916
C	-0.985568	-2.185019	-1.047701
C	-0.012670	-3.236401	-1.116017
C	0.060428	-4.033529	-2.269475
C	0.843785	-3.484128	-0.031445
C	0.979711	-5.072200	-2.334381
H	-0.607678	-3.832754	-3.101483
C	1.758813	-4.526709	-0.114806
H	0.769852	-2.864866	0.862327
C	1.831171	-5.319256	-1.258684
H	1.030515	-5.691425	-3.225293
H	2.417255	-4.723185	0.726201
H	2.548163	-6.133802	-1.311722
F	-1.163232	0.117002	1.527377
C	-1.222234	-3.106305	6.020494
C	-1.949815	-2.151536	5.311489
C	-1.441690	-1.616860	4.128028
C	-0.199374	-2.023646	3.630887
C	0.523874	-2.984444	4.353003
C	0.019121	-3.517398	5.528925
H	-2.917520	-1.811350	5.665452
H	-1.998683	-0.873312	3.566212
H	1.491834	-3.303014	3.975703
H	0.576714	-4.261153	6.090661
O	0.318658	-1.524484	2.495463
H	-0.318416	-0.803635	2.092769
O	-1.629513	-3.692300	7.189384
C	-2.873270	-3.297564	7.701171
H	-3.694818	-3.522356	7.005967
H	-3.023410	-3.865069	8.622075
H	-2.900031	-2.223750	7.935980

TS-3

C	-0.947820	-2.529792	-1.720827
C	-1.825537	-1.513926	-1.906751
H	0.092009	-2.535489	-1.435682
H	-1.715525	-0.445135	-1.817796
C	-1.080928	-5.013485	-1.821962
C	-0.971319	-5.537495	-0.523030
C	-0.654956	-5.693384	-2.969039
C	-0.397419	-6.802614	-0.396479
C	-0.082080	-6.953946	-2.784448
C	0.050588	-7.498725	-1.514746
H	-0.292121	-7.245609	0.588140
H	0.259081	-7.514807	-3.649328
H	0.504362	-8.478259	-1.392589
C	-4.232584	-1.373781	-2.545706
C	-4.416840	-0.860422	-3.838086
C	-5.168399	-1.221105	-1.513005
C	-5.586668	-0.137214	-4.075164
C	-6.320587	-0.487342	-1.803655
C	-6.521462	0.058962	-3.064742
H	-5.771072	0.271759	-5.063507
H	-7.071892	-0.350193	-1.032023
H	-7.421602	0.632230	-3.266914
N	-1.641102	-3.701824	-1.966286
N	-3.025549	-2.095773	-2.269253
C	-0.788267	-5.112890	-4.361730
H	-1.254733	-4.125905	-4.277799
C	-1.703256	-5.976352	-5.232621
H	-1.294401	-6.982976	-5.367774
H	-2.694660	-6.079818	-4.781390
H	-1.821147	-5.523531	-6.222323
C	0.583947	-4.919203	-5.010398

H	1.226366	-4.277760	-4.399148
H	1.098256	-5.876041	-5.147363
H	0.475340	-4.456083	-5.996214
C	-3.430993	-1.132721	-4.956187
H	-2.497572	-1.489466	-4.507291
C	-3.096554	0.122101	-5.761830
H	-3.966752	0.495928	-6.310532
H	-2.733100	0.927425	-5.116425
H	-2.320079	-0.103063	-6.499218
C	-3.963325	-2.249401	-5.860531
H	-4.150093	-3.165882	-5.290632
H	-4.905705	-1.951789	-6.332380
H	-3.242067	-2.478144	-6.652095
C	-1.497703	-4.779756	0.680000
H	-1.433195	-3.709137	0.454347
C	-0.681034	-5.021503	1.947519
H	-1.027748	-4.354021	2.741877
H	-0.801954	-6.045036	2.316771
H	0.385747	-4.837880	1.786379
C	-2.971838	-5.121199	0.917170
H	-3.577886	-4.980799	0.017135
H	-3.082318	-6.160671	1.236075
H	-3.387551	-4.486883	1.707456
C	-4.982768	-1.839824	-0.142829
H	-4.003352	-2.328431	-0.116680
C	-6.038776	-2.918915	0.110528
H	-6.005694	-3.701766	-0.653479
H	-5.870911	-3.395305	1.081150
H	-7.048421	-2.495268	0.109433
C	-4.993271	-0.777765	0.958054
H	-4.224830	-0.017381	0.787285
H	-5.961701	-0.270226	1.015885
H	-4.805576	-1.242703	1.930916
C	-2.921210	-3.442246	-2.310505
Au	-4.408764	-4.686392	-2.859088
C	-8.614293	-5.522805	-4.031215
C	-7.430505	-4.822239	-3.746102
C	-7.431110	-3.421686	-3.727653
C	-8.605450	-2.725348	-3.987012
C	-9.779752	-3.417506	-4.272647
C	-9.777304	-4.812158	-4.294671
H	-8.568503	-6.611037	-4.011228
H	-6.513230	-2.882326	-3.513057
H	-8.597382	-1.640131	-3.968368
H	-10.696305	-2.871114	-4.477324
H	-10.694560	-5.352277	-4.511527
C	-6.226430	-5.585042	-3.507079
C	-5.674228	-6.709052	-3.396006
C	-4.815747	-7.851267	-3.470934
C	-5.144026	-8.868788	-4.382264
C	-3.671908	-7.974493	-2.670038
C	-4.320925	-9.978478	-4.499604
H	-6.077031	-8.786591	-4.923542
C	-2.858549	-9.094121	-2.797222
H	-3.438739	-7.208234	-1.937318
C	-3.175240	-10.094156	-3.712058
H	-4.582691	-10.765994	-5.199898
H	-1.979361	-9.182533	-2.168760
H	-2.539088	-10.969987	-3.802414
F	-7.547211	-8.099493	-3.330359
C	-4.983056	-7.988713	1.401535
C	-5.852748	-6.927145	1.664202
C	-6.888131	-6.629545	0.790369
C	-7.090968	-7.389746	-0.376133
C	-6.232101	-8.473865	-0.606596
C	-5.184547	-8.766347	0.264064
H	-5.689178	-6.331120	2.557825
H	-7.552961	-5.793345	0.987372
H	-6.385771	-9.056777	-1.508343
H	-4.528463	-9.597250	0.027804
O	-8.072681	-7.069598	-1.216021
H	-7.899567	-7.551763	-2.133523
O	-3.954715	-8.167330	2.297041
C	-3.001717	-9.146189	1.971988
H	-2.526814	-8.943067	1.000862
H	-2.243465	-9.114141	2.757187
H	-3.439448	-10.153069	1.941460

Int-3

C	3.419970	2.554334	0.457637
C	2.478323	3.526689	0.374824
H	4.477445	2.597394	0.663719
H	2.541109	4.596174	0.496238
C	3.405971	0.073171	0.206623
C	3.605176	-0.578473	1.433657
C	3.798631	-0.468853	-1.023549
C	4.258527	-1.810922	1.402471
C	4.439095	-1.710051	-1.000418
C	4.676849	-2.368038	0.198301
H	4.435519	-2.345442	2.330315
H	4.753645	-2.164035	-1.935391
H	5.181672	-3.329799	0.195319
C	0.017954	3.540391	-0.074089
C	-0.395094	3.904586	-1.362647
C	-0.764798	3.749296	1.069678
C	-1.638138	4.527901	-1.483063
C	-2.000130	4.375502	0.895111
C	-2.428952	4.768112	-0.366932
H	-1.996146	4.814795	-2.467233
H	-2.637258	4.548008	1.757410
H	-3.393719	5.253440	-0.483021

N	2.769624	1.356391	0.217870
N	1.285706	2.891129	0.084105
C	3.540966	0.231010	-2.341672
H	3.052616	1.187395	-2.129559
C	2.581464	-0.585193	-3.210880
H	3.013539	-1.555928	-3.476114
H	1.637061	-0.764042	-2.686118
H	2.361475	-0.048136	-4.139421
C	4.848147	0.533457	-3.076523
H	5.519939	1.140345	-2.461431
H	5.378100	-0.386601	-3.344396
H	4.644311	1.080340	-4.002616
C	0.433760	3.610756	-2.595602
H	1.378155	3.161346	-2.270977
C	0.772883	4.890248	-3.362043
H	-0.129720	5.377912	-3.744762
H	1.298363	5.609410	-2.725907
H	1.412961	4.659847	-4.219676
C	-0.275108	2.589738	-3.489433
H	-0.474271	1.663317	-2.941447
H	-1.233405	2.976593	-3.851427
H	0.345625	2.351462	-4.359737
C	3.067162	-0.005576	2.729265
H	2.963459	1.077801	2.599910
C	3.998759	-0.232699	3.918144
H	3.617338	0.299466	4.794793
H	4.061130	-1.291717	4.188760
H	5.012037	0.126300	3.713053
C	1.671790	-0.573965	3.008650
H	0.982456	-0.378016	2.181761
H	1.708847	-1.656942	3.161407
H	1.250605	-0.126332	3.914813
C	-0.337272	3.261109	2.438508
H	0.708718	2.942565	2.376140
C	-1.165605	2.036695	2.836172
H	-1.062240	1.233795	2.099711
H	-0.840148	1.651206	3.808075
H	-2.228297	2.293078	2.910775
C	-0.417994	4.361003	3.497001
H	0.174966	5.235203	3.211053
H	-1.449025	4.692269	3.657661
H	-0.040901	3.989638	4.455056
C	1.451674	1.551181	-0.017872
Au	0.009044	0.228278	-0.546136
C	-3.365121	-0.489944	-2.865788
C	-2.704913	-0.155107	-1.672066
C	-3.123315	1.005602	-1.000733
C	-4.168553	1.782138	-1.485508
C	-4.811065	1.436120	-2.672282
C	-4.398928	0.297789	-3.361445
H	-3.052311	-1.374498	-3.410929
H	-2.612459	1.296623	-0.086690
H	-4.470782	2.670220	-0.938147
H	-5.620545	2.049108	-3.058668
H	-4.885302	0.019544	-4.292826
C	-1.551777	-0.935220	-1.166049
C	-1.611382	-2.276127	-1.134652
C	-0.638629	-3.304421	-0.749263
C	-0.931896	-4.653716	-1.005609
C	0.567729	-3.008830	-0.097719
C	-0.061733	-5.665280	-0.613996
H	-1.857773	-4.906591	-1.508562
C	1.434397	-4.020138	0.292713
H	0.817454	-1.975577	0.127234
C	1.126441	-5.356918	0.042271
H	-0.317084	-6.700267	-0.824388
H	2.356330	-3.753978	0.799876
H	1.805940	-6.146098	0.351747
F	-2.817438	-2.880964	-1.482176
C	-1.188524	-2.933334	3.467283
C	-1.757590	-1.661153	3.582188
C	-2.768671	-1.260929	2.721492
C	-3.238659	-2.131745	1.735784
C	-2.705481	-3.413745	1.656815
C	-1.675400	-3.814502	2.504857
H	-1.373386	-0.986407	4.340817
H	-3.188159	-0.262086	2.783837
H	-3.063365	-4.095854	0.892219
H	-1.247366	-4.801575	2.374270
O	-4.207283	-1.692260	0.883970
H	-4.059882	-2.103930	0.021670
O	-0.166327	-3.216070	4.327000
C	0.475934	-4.458948	4.164022
H	0.888195	-4.569753	3.152570
H	1.288104	-4.482108	4.892671
H	-0.205144	-5.297319	4.361948

TS-4

C	0.095651	-2.279073	-2.240495
C	-0.746443	-1.224360	-2.113633
H	1.156613	-2.328384	-2.426506
H	-0.571516	-0.161428	-2.161664
C	-0.150868	-4.755694	-2.140927
C	0.247167	-5.373730	-0.945077
C	-0.053200	-5.378849	-3.394658
C	0.763518	-6.667671	-1.035586
C	0.475306	-6.670775	-3.426492
C	0.879067	-7.309753	-2.261664
H	1.076726	-7.179181	-0.129806
H	0.563251	-7.184723	-4.379230
H	1.283342	-8.316838	-2.309267

C	-3.183006	-0.952896	-1.655321
C	-4.022188	-0.672735	-2.746720
C	-3.432850	-0.493433	-0.353438
C	-5.149393	0.110266	-2.493567
C	-4.575602	0.285160	-0.159554
C	-5.423660	0.584952	-1.216944
H	-5.827299	0.341853	-3.308470
H	-4.805398	0.653150	0.836376
H	-6.309948	1.189386	-1.045397
N	-0.670203	-3.417492	-2.080399
N	-2.003246	-1.745478	-1.878879
C	-0.488062	-4.704677	-4.682083
H	-0.955287	-3.748083	-4.424813
C	-1.536940	-5.534738	-5.426609
H	-1.127431	-6.492864	-5.763666
H	-2.404627	-5.746510	-4.795666
H	-1.882185	-4.994094	-6.314106
C	0.719293	-4.403429	-5.576276
H	1.453639	-3.772403	-5.064982
H	1.227114	-5.325748	-5.878299
H	0.400657	-3.884978	-6.486406
C	-3.711620	-1.151672	-4.152872
H	-3.046805	-2.018912	-4.069175
C	-2.963795	-0.059775	-4.928768
H	-3.586350	0.835938	-5.030873
H	-2.034059	0.235466	-4.430737
H	-2.712148	-0.410432	-5.935224
C	-4.954486	-1.616088	-4.913779
H	-5.517514	-2.366074	-4.346752
H	-5.620989	-0.778802	-5.150055
H	-4.651377	-2.056421	-5.869855
C	0.125115	-4.700187	0.408711
H	-0.271764	-3.691401	0.253300
C	1.489957	-4.556517	1.088512
H	1.386782	-4.024446	2.039820
H	1.935476	-5.533494	1.303538
H	2.194286	-4.000390	0.461858
C	-0.867705	-5.445493	1.306007
H	-1.850620	-5.516484	0.830198
H	-0.522492	-6.462251	1.522270
H	-0.985218	-4.921946	2.260688
C	-2.531336	-0.821095	0.822535
H	-1.683378	-1.406790	0.451165
C	-3.261831	-1.683709	1.855585
H	-3.643938	-2.605284	1.407458
H	-2.584414	-1.950339	2.674274
H	-4.115190	-1.151779	2.288250
C	-1.961540	0.448730	1.461898
H	-1.414081	1.055870	0.733804
H	-2.754908	1.073616	1.885200
H	-1.275449	0.190958	2.275409
C	-1.968154	-3.099661	-1.855274
Au	-3.525145	-4.349338	-1.637826
C	-5.193455	-5.748233	-1.733573
C	-5.228465	-5.806766	-0.363272
C	-4.854275	-6.876993	-2.632866
C	-5.120037	-6.668905	-4.000382
C	-4.304645	-8.114771	-2.251713
C	-4.854242	-7.653999	-4.944345
H	-5.558597	-5.718697	-4.296094
C	-4.033989	-9.093606	-3.201852
H	-4.086405	-8.312106	-1.210346
C	-4.306330	-8.872153	-4.550048
H	-5.081314	-7.469651	-5.990924
H	-3.608531	-10.041604	-2.883003
H	-4.098193	-9.644941	-5.284990
C	-5.773618	-4.814801	0.582019
C	-5.686845	-5.057372	1.960498
C	-6.420051	-3.652516	0.127732
C	-6.229016	-4.154651	2.868689
H	-5.202046	-5.959459	2.315751
C	-6.959269	-2.760475	1.044576
H	-6.505883	-3.462100	-0.941172
C	-6.867603	-3.004041	2.414894
H	-6.157763	-4.356823	3.933643
H	-7.464442	-1.871008	0.680012
H	-7.299480	-2.304046	3.124948
F	-4.841721	-6.930939	0.289674
C	-7.872752	-4.033686	-3.211309
C	-8.650260	-2.882306	-3.425851
C	-10.025686	-2.948534	-3.646771
C	-10.674737	-4.182144	-3.649457
C	-9.924575	-5.339767	-3.432273
C	-8.555965	-5.270664	-3.221381
H	-8.148209	-1.918012	-3.418994
H	-10.577190	-2.028125	-3.812068
H	-10.439586	-6.296540	-3.437838
H	-7.988520	-6.185331	-3.069517
O	-6.563575	-3.940784	-3.013395
H	-5.923224	-4.861485	-2.324813
O	-12.019753	-4.362124	-3.852970
C	-12.787698	-3.213102	-4.076393
H	-13.817266	-3.549569	-4.217711
H	-12.754007	-2.520798	-3.222233
H	-12.464721	-2.668677	-4.975973

TS-5

C	-1.115652	-1.265784	-1.398512
C	-2.206025	-0.478036	-1.227175
H	-0.061035	-1.042527	-1.380414
H	-2.302783	0.577518	-1.030389

C	-0.769652	-3.707195	-1.828937
C	-0.504921	-4.530524	-0.727299
C	-0.300713	-3.973697	-3.123542
C	0.280494	-5.662925	-0.953778
C	0.478113	-5.118034	-3.295626
C	0.766912	-5.952909	-2.221529
H	0.506432	-6.327598	-0.125218
H	0.856263	-5.365048	-4.282410
H	1.372728	-6.840804	-2.377084
C	-4.679339	-0.892176	-1.243910
C	-5.357936	-0.518024	-2.411068
C	-5.287103	-0.934866	0.017816
C	-6.702806	-0.169670	-2.283292
C	-6.632329	-0.571597	0.090702
C	-7.334980	-0.196245	-1.046932
H	-7.268659	0.104907	-3.168384
H	-7.141083	-0.606510	1.049679
H	-8.390282	0.049096	-0.976894
N	-1.585436	-2.546922	-1.629296
N	-3.310257	-1.300212	-1.355283
C	-0.686821	-3.099834	-4.300226
H	-0.970288	-2.115508	-3.910565
C	-1.915195	-3.687524	-5.003822
H	-1.683741	-4.674324	-5.419004
H	-2.752374	-3.804007	-4.308077
H	-2.236050	-3.037237	-5.824589
C	0.461154	-2.883244	-5.284422
H	1.354456	-2.499120	-4.782578
H	0.734324	-3.809075	-5.800753
H	0.162740	-2.162482	-6.051851
C	-4.694241	-0.531458	-3.772701
H	-3.630876	-0.752872	-3.632281
C	-4.791403	0.830185	-4.462227
H	-5.831800	1.100409	-4.670332
H	-4.356760	1.622225	-3.844596
H	-4.258425	0.809621	-5.418181
C	-5.281507	-1.643846	-4.644355
H	-5.169906	-2.618446	-4.159455
H	-6.348859	-1.478671	-4.824413
H	-4.773522	-1.677856	-5.613901
C	-1.033541	-4.231164	0.660383
H	-1.656427	-3.332863	0.601061
C	0.113902	-3.931290	1.627711
H	-0.279773	-3.675882	2.616820
H	0.770604	-4.799888	1.744536
H	0.725018	-3.094117	1.275594
C	-1.921372	-5.365965	1.175861
H	-2.752120	-5.571235	0.493743
H	-1.348132	-6.290783	1.302265
H	-2.337701	-5.099116	2.153088
C	-4.550530	-1.404621	1.255464
H	-3.496521	-1.544335	0.992120
C	-5.090140	-2.760188	1.718860
H	-5.004943	-3.510027	0.926451
H	-4.532161	-3.116084	2.591334
H	-6.147006	-2.686955	1.997127
C	-4.606596	-0.367043	2.377394
H	-4.207519	0.597622	2.049107
H	-5.632909	-0.205032	2.722779
H	-4.019699	-0.706487	3.236759
C	-2.936925	-2.577632	-1.601776
Au	-4.144645	-4.109789	-1.777381
F	-4.788890	-6.179525	-0.781197
C	-8.583348	-3.659862	-0.397878
C	-7.500050	-4.455453	-0.767862
C	-7.106022	-4.548331	-2.103582
C	-7.837551	-3.852994	-3.072613
C	-8.914333	-3.060684	-2.707261
C	-9.286945	-2.945508	-1.365767
H	-8.854180	-3.599349	0.650312
H	-6.940584	-5.001120	-0.013904
H	-7.538723	-3.936031	-4.112757
H	-9.478245	-2.507269	-3.452102
O	-6.024708	-5.281071	-2.487538
H	-5.583708	-5.885338	-1.641624
O	-10.331857	-2.105094	-1.103539
C	-10.721209	-1.971356	0.241384
H	-9.907010	-1.572068	0.861941
H	-11.556122	-1.268983	0.252714
H	-11.051007	-2.927922	0.667583

TS-1’

C	-0.583418	-1.826733	-2.451966
C	-1.266367	-0.656549	-2.532013
H	0.455189	-2.035823	-2.252159
H	-0.947969	0.366779	-2.414361
C	-1.227742	-4.239917	-2.724811
C	-1.382645	-4.980401	-1.544854
C	-0.878247	-4.817325	-3.953183
C	-1.148485	-6.354423	-1.617134
C	-0.651493	-6.195214	-3.970772
C	-0.780152	-6.955079	-2.814589
H	-1.269371	-6.962388	-0.725591
H	-0.381141	-6.678111	-4.905206
H	-0.601159	-8.026004	-2.849207
C	-3.663807	-0.067074	-3.003054
C	-3.866815	0.473044	-4.280712
C	-4.500545	0.196380	-1.911305
C	-4.971800	1.307812	-4.449412
C	-5.591209	1.039291	-2.133606
C	-5.826822	1.584923	-3.388943

H	-5.173760	1.736257	-5.425822
H	-6.267660	1.263590	-1.314000
H	-6.686152	2.230407	-3.544342
N	-1.500683	-2.832890	-2.690863
N	-2.578327	-0.984910	-2.821323
C	-0.788384	-4.012264	-5.233117
H	-0.973086	-2.960777	-4.989612
C	-1.874340	-4.450072	-6.218828
H	-1.741813	-5.496146	-6.514717
H	-2.871550	-4.347190	-5.781403
H	-1.835572	-3.837194	-7.125029
C	0.605557	-4.101757	-5.856290
H	1.377504	-3.765897	-5.156993
H	0.845623	-5.127930	-6.153364
H	0.660106	-3.476504	-6.752969
C	-2.965575	0.123483	-5.448400
H	-2.026775	-0.271162	-5.043118
C	-2.611674	1.342742	-6.299349
H	-3.486967	1.739814	-6.823157
H	-2.186808	2.147910	-5.692172
H	-1.877750	1.064548	-7.061945
C	-3.599957	-0.980363	-6.300059
H	-3.792823	-1.878930	-5.707946
H	-4.557395	-0.649491	-6.714651
H	-2.938647	-1.247618	-7.131186
C	-1.834971	-4.349335	-0.244361
H	-1.877571	-3.264514	-0.389126
C	-0.847109	-4.621502	0.891013
H	-1.168662	-4.108063	1.802578
H	-0.783596	-5.690251	1.120010
H	0.158585	-4.272178	0.637746
C	-3.246698	-4.820911	0.115442
H	-3.954332	-4.577875	-0.683857
H	-3.274124	-5.904709	0.268789
H	-3.586396	-4.337735	1.037554
C	-4.263499	-0.405683	-0.541220
H	-3.342994	-0.997418	-0.583389
C	-5.398503	-1.356042	-0.153892
H	-5.503889	-2.155641	-0.894559
H	-5.196441	-1.813947	0.819965
H	-6.354206	-0.825748	-0.086283
C	-4.060285	0.682488	0.514934
H	-3.232116	1.346145	0.248040
H	-4.958256	1.297852	0.631954
H	-3.838472	0.231238	1.487286
C	-2.732561	-2.322864	-2.919290
Au	-4.374293	-3.392856	-3.239156
C	-5.790089	-5.983532	-3.135945
C	-6.816079	-6.731585	-2.543079
C	-4.573473	-6.618264	-3.397613
C	-6.625762	-8.064821	-2.214771
H	-7.774055	-6.259785	-2.337762
C	-4.376343	-7.956066	-3.075884
H	-3.763144	-6.054883	-3.852820
C	-5.404123	-8.689239	-2.478711
H	-7.417663	-8.644270	-1.751035
H	-3.413295	-8.406022	-3.286286
O	-5.306952	-9.995642	-2.112382
C	-4.088275	-10.650138	-2.374195
H	-3.252143	-10.179848	-1.839336
H	-4.207989	-11.674461	-2.018837
H	-3.858862	-10.666163	-3.447502
N	-6.002098	-4.635586	-3.515914
H	-6.828399	-4.286634	-3.036780
C	-8.915660	-5.300427	-5.585304
C	-7.561214	-5.530048	-5.587337
C	-6.663503	-4.460512	-5.306189
C	-7.291670	-3.183044	-5.343841
C	-8.662024	-3.097557	-5.340185
N	-9.491487	-4.118196	-5.455107
F	-9.216145	-1.884156	-5.238995
F	-6.524214	-2.081390	-5.202772
F	-5.428680	-4.564771	-5.877802
F	-7.056390	-6.761735	-5.765303
F	-9.730987	-6.345139	-5.758202

Int-1'

C	-0.506935	-2.179888	-1.408577
C	-1.200617	-1.015396	-1.397551
H	0.530173	-2.397156	-1.209226
H	-0.898052	-0.002854	-1.184524
C	-1.085143	-4.566515	-1.853959
C	-0.968294	-5.311840	-0.669909
C	-0.899630	-5.111583	-3.130435
C	-0.646956	-6.662292	-0.799858
C	-0.585479	-6.470948	-3.203869
C	-0.457371	-7.235745	-2.052752
H	-0.553440	-7.275260	0.090143
H	-0.433011	-6.930161	-4.176213
H	-0.206201	-8.290037	-2.131066
C	-3.585793	-0.402469	-1.844479
C	-3.789051	0.239167	-3.073624
C	-4.409597	-0.210500	-0.727877
C	-4.870119	1.116876	-3.163484
C	-5.475624	0.679829	-0.869643
C	-5.703740	1.336040	-2.072848
H	-5.067885	1.626289	-4.101847
H	-6.139146	0.853535	-0.027477
H	-6.542090	2.020509	-2.163788
N	-1.407520	-3.174815	-1.745368
N	-2.502575	-1.332319	-1.736295

C	-1.001878	-4.283930	-4.395340
H	-1.303566	-3.269455	-4.116673
C	-2.074920	-4.829981	-5.338279
H	-1.827424	-5.838224	-5.686678
H	-3.048734	-4.869834	-4.840284
H	-2.167074	-4.185567	-6.218580
C	0.358140	-4.185531	-5.090667
H	1.115146	-3.759500	-4.424754
H	0.711489	-5.170945	-5.412331
H	0.286587	-3.549785	-5.979111
C	-2.916564	-0.042842	-4.279974
H	-2.046448	-0.616178	-3.942286
C	-2.392977	1.240648	-4.924848
H	-3.206194	1.844454	-5.340346
H	-1.851059	1.858630	-4.202389
H	-1.712366	0.998552	-5.747119
C	-3.671996	-0.909118	-5.292112
H	-4.014441	-1.842209	-4.832790
H	-4.550909	-0.381072	-5.677175
H	-3.025799	-1.157279	-6.140941
C	-1.234024	-4.688689	0.686825
H	-0.969601	-3.626458	0.625076
C	-0.388028	-5.300212	1.801640
H	-0.520576	-4.725172	2.723016
H	-0.692556	-6.328907	2.020138
H	0.677354	-5.303963	1.550504
C	-2.724130	-4.778531	1.031233
H	-3.350822	-4.324193	0.258958
H	-3.035807	-5.822367	1.128848
H	-2.924021	-4.269444	1.980574
C	-4.181360	-0.933451	0.583908
H	-3.301537	-1.575302	0.471933
C	-5.362552	-1.841405	0.932497
H	-5.535024	-2.596979	0.159646
H	-5.166380	-2.366259	1.873258
H	-6.283248	-1.261145	1.058416
C	-3.885490	0.058375	1.711219
H	-3.022953	0.688616	1.472558
H	-4.740455	0.717286	1.896367
H	-3.671361	-0.477615	2.641369
C	-2.642117	-2.660423	-1.955944
Au	-4.279739	-3.577248	-2.509050
F	-5.964079	-4.488779	-3.090000
C	-3.987850	-8.489190	0.586238
C	-3.950981	-7.947973	-0.698420
C	-4.784912	-6.885237	-1.036622
C	-5.668194	-6.324498	-0.104375
C	-5.698349	-6.886276	1.182917
C	-4.876155	-7.951146	1.520312
H	-3.274120	-8.336176	-1.450856
H	-4.750193	-6.475339	-2.041865
H	-6.371968	-6.472337	1.929654
H	-4.907401	-8.382793	2.516349
O	-3.212838	-9.531390	1.018175
C	-2.410127	-10.167890	0.056248
H	-1.650064	-9.490997	-0.356589
H	-1.910406	-10.992991	0.566755
H	-3.008372	-10.566651	-0.774045
N	-6.432239	-5.213396	-0.423069
H	-6.487238	-4.995789	-1.417961
H	-7.310257	-5.136315	0.066423

TS-2'

C	2.913228	2.631000	-1.135066
C	1.948245	3.505651	-1.506353
H	3.984507	2.735189	-1.069922
H	2.001417	4.531562	-1.834085
C	2.938256	0.288888	-0.280922
C	3.131694	0.197800	1.106916
C	3.373899	-0.694244	-1.184418
C	3.782427	-0.943377	1.585090
C	4.024224	-1.808863	-0.652418
C	4.223993	-1.934746	0.717552
H	3.934726	-1.057910	2.654415
H	4.377176	-2.588885	-1.320644
H	4.728446	-2.812272	1.112676
C	-0.548302	3.408663	-1.638338
C	-1.092642	3.310320	-2.928111
C	-1.219433	4.020827	-0.567475
C	-2.365197	3.847606	-3.129029
C	-2.492367	4.534879	-0.823983
C	-3.060954	4.448801	-2.087862
H	-2.820061	3.784741	-4.113701
H	-3.047375	5.003736	-0.016688
H	-4.054296	4.852346	-2.262442
N	2.273465	1.451133	-0.799409
N	0.744064	2.835616	-1.392331
C	3.196121	-0.553563	-2.684559
H	2.508834	0.279796	-2.864369
C	2.569350	-1.797239	-3.316671
H	3.225030	-2.670507	-3.234056
H	1.614741	-2.047039	-2.846660
H	2.390981	-1.624697	-4.383271
C	4.532978	-0.209885	-3.351378
H	4.963336	0.710208	-2.942799
H	5.263051	-1.012736	-3.201075
H	4.401205	-0.073469	-4.429994
C	-0.364982	2.629456	-4.071775
H	0.629393	2.339581	-3.716024
C	-0.170397	3.574483	-5.260702
H	-1.129058	3.878584	-5.694135

H	0.365193	4.483083	-4.968064
H	0.404828	3.079235	-6.049851
C	-1.088782	1.346109	-4.491598
H	-1.183636	0.661186	-3.643060
H	-2.093383	1.565412	-4.869996
H	-0.533822	0.838445	-5.288143
C	2.699335	1.283078	2.075573
H	2.190359	2.065523	1.502540
C	3.925954	1.923802	2.735947
H	3.616857	2.746929	3.388399
H	4.467624	1.198915	3.352996
H	4.628132	2.320773	1.994754
C	1.705313	0.767948	3.120836
H	0.779461	0.405334	2.660328
H	2.133759	-0.043192	3.719674
H	1.450632	1.581217	3.810154
C	-0.620096	4.136585	0.822533
H	0.411260	3.770050	0.780262
C	-1.371402	3.269450	1.838949
H	-1.295943	2.201349	1.610281
H	-0.952165	3.423908	2.839338
H	-2.432938	3.538510	1.876576
C	-0.560683	5.598810	1.277077
H	-0.021368	6.224764	0.558689
H	-1.562579	6.022766	1.402666
H	-0.051451	5.671781	2.243400
C	0.931006	1.563383	-0.958534
Au	-0.513492	0.240675	-0.555395
C	-4.816195	0.194753	-1.133722
C	-3.743902	-0.086863	-0.271965
C	-3.785965	0.329753	1.068040
C	-4.904481	1.013028	1.530012
C	-5.972208	1.288589	0.677472
C	-5.925100	0.879666	-0.654470
H	-4.772000	-0.132476	-2.168061
H	-2.914811	0.112702	1.687180
H	-4.941784	1.333610	2.567438
H	-6.842757	1.821910	1.049514
H	-6.756124	1.092150	-1.320946
C	-2.625844	-0.833644	-0.769231
C	-1.838477	-1.634197	-1.278243
C	-1.131791	-2.721437	-1.886868
C	-1.271944	-2.954601	-3.264884
C	-0.337370	-3.579577	-1.108394
C	-0.630653	-4.035950	-3.854961
H	-1.888165	-2.285548	-3.857851
C	0.297279	-4.657471	-1.715270
H	-0.220523	-3.391796	-0.039873
C	0.154409	-4.890224	-3.081910
H	-0.745930	-4.214490	-4.920160
H	0.906216	-5.322956	-1.110033
H	0.652308	-5.737272	-3.545448
F	-1.001838	-0.280901	1.646749
C	0.693750	-3.886897	5.883041
C	-0.336753	-3.049840	5.454984
C	-0.356370	-2.573155	4.146269
C	0.640105	-2.924542	3.227243
C	1.672325	-3.765711	3.676045
C	1.700458	-4.235292	4.980564
H	-1.129784	-2.750264	6.132183
H	-1.143681	-1.900224	3.817648
H	2.458600	-4.059253	2.983290
H	2.500163	-4.885404	5.323314
O	0.809440	-4.406071	7.144332
C	-0.193874	-4.074047	8.065928
H	-1.185127	-4.415823	7.735695
H	0.063080	-4.580491	8.998640
H	-0.242047	-2.991231	8.250081
N	0.573544	-2.484021	1.909686
H	1.483915	-2.350035	1.489611
H	-0.025686	-1.649050	1.786073

Int-2’

C	2.925337	2.632267	-1.049882
C	1.970858	3.519945	-1.418538
H	3.992702	2.737198	-0.938109
H	2.031419	4.559199	-1.699474
C	2.930542	0.263291	-0.276045
C	3.193461	0.194942	1.100770
C	3.284317	-0.753080	-1.181030
C	3.833036	-0.957345	1.569269
C	3.920138	-1.879177	-0.657485
C	4.190895	-1.981681	0.703256
H	4.037964	-1.054259	2.631395
H	4.205507	-2.688196	-1.322135
H	4.683803	-2.869029	1.090902
C	-0.520940	3.427166	-1.622127
C	-1.056675	3.345558	-2.916759
C	-1.194299	4.031208	-0.548015
C	-2.326099	3.889450	-3.119171
C	-2.464919	4.550368	-0.806810
C	-3.026641	4.479445	-2.074677
H	-2.774022	3.841849	-4.107898
H	-3.022524	5.012869	0.002332
H	-4.017732	4.888028	-2.250528
N	2.278381	1.439126	-0.782800
N	0.766717	2.843765	-1.371626
C	3.043659	-0.614240	-2.673413
H	2.198036	0.068544	-2.810380
C	2.665730	-1.931911	-3.348725
H	3.503754	-2.637284	-3.368270

H	1.822837	-2.414894	-2.847727
H	2.381397	-1.745020	-4.389186
C	4.270463	0.008734	-3.351762
H	4.513738	0.989665	-2.931703
H	5.148439	-0.634859	-3.228099
H	4.093122	0.136046	-4.425068
C	-0.317626	2.691438	-4.068740
H	0.653023	2.346706	-3.696919
C	-0.050719	3.690297	-5.198695
H	-0.984826	4.057269	-5.637173
H	0.512303	4.558603	-4.842131
H	0.526195	3.215556	-5.999217
C	-1.070148	1.459510	-4.580516
H	-1.218802	0.735377	-3.773176
H	-2.052041	1.732050	-4.982716
H	-0.503822	0.974229	-5.382955
C	2.840356	1.307168	2.071540
H	2.340823	2.103720	1.509882
C	4.113764	1.905690	2.680728
H	3.860128	2.748575	3.331762
H	4.647286	1.168010	3.289629
H	4.806578	2.264750	1.911615
C	1.862719	0.840621	3.154551
H	0.903286	0.533770	2.721926
H	2.270427	0.004720	3.733546
H	1.678851	1.663605	3.854930
C	-0.597943	4.144755	0.843797
H	0.431745	3.773072	0.805639
C	-1.353123	3.285485	1.864336
H	-1.240687	2.211815	1.672192
H	-0.960592	3.481738	2.868611
H	-2.420777	3.533391	1.872174
C	-0.531390	5.610226	1.288501
H	0.007660	6.231057	0.565112
H	-1.531494	6.038237	1.414952
H	-0.019160	5.686535	2.252944
C	0.943393	1.557562	-0.982599
Au	-0.534229	0.206649	-0.738152
C	-4.836460	-0.147259	-1.012750
C	-3.682433	-0.239467	-0.218445
C	-3.676639	0.271287	1.088525
C	-4.833232	0.859525	1.587220
C	-5.982234	0.948113	0.803525
C	-5.981528	0.445087	-0.496793
H	-4.826259	-0.546389	-2.022513
H	-2.744173	0.198296	1.653687
H	-4.835795	1.253241	2.599829
H	-6.880802	1.409221	1.204403
H	-6.875940	0.512202	-1.109472
C	-2.521419	-0.896105	-0.752106
C	-1.733107	-1.677842	-1.301305
C	-1.056544	-2.793307	-1.899273
C	-1.216534	-3.057470	-3.268369
C	-0.275443	-3.647354	-1.103715
C	-0.609277	-4.171378	-3.833922
H	-1.820695	-2.388490	-3.873713
C	0.324730	-4.758249	-1.685958
H	-0.147717	-3.421413	-0.043251
C	0.160704	-5.024313	-3.044449
H	-0.739043	-4.376182	-4.892653
H	0.922749	-5.423398	-1.069545
H	0.631817	-5.896388	-3.489151
F	-0.842455	0.027786	1.655587
C	0.485515	-3.805468	5.792469
C	-0.450992	-2.856755	5.381867
C	-0.414158	-2.349850	4.084841
C	0.544610	-2.781790	3.160315
C	1.483367	-3.733844	3.592003
C	1.456739	-4.233566	4.885259
H	-1.211577	-2.491960	6.064284
H	-1.121217	-1.587269	3.769985
H	2.239923	-4.089125	2.895042
H	2.184198	-4.969464	5.215315
O	0.540112	-4.363882	7.041163
C	-0.428597	-3.952124	7.967522
H	-1.448056	-4.181260	7.625920
H	-0.230699	-4.504843	8.888301
H	-0.365584	-2.874908	8.178148
N	0.528523	-2.307664	1.851705
H	1.453729	-2.232055	1.449110
H	0.000439	-1.421139	1.746552

TS-3’

C	-0.918569	-2.627901	-1.719974
C	-1.822374	-1.624212	-1.834317
H	0.130293	-2.623169	-1.470139
H	-1.731198	-0.557919	-1.703752
C	-0.995844	-5.104807	-1.961778
C	-0.831839	-5.720839	-0.709947
C	-0.588644	-5.685268	-3.168800
C	-0.202797	-6.965515	-0.693844
C	0.034703	-6.933218	-3.095389
C	0.235957	-7.559033	-1.873452
H	-0.054368	-7.477927	0.250537
H	0.363159	-7.418607	-4.009398
H	0.729054	-8.526195	-1.837783
C	-4.250933	-1.510314	-2.396623
C	-4.494229	-0.970513	-3.668273
C	-5.149008	-1.401921	-1.325990
C	-5.689325	-0.271453	-3.844208
C	-6.330414	-0.693845	-1.556662

C	-6.592022	-0.125432	-2.796706
H	-5.919890	0.157106	-4.814323
H	-7.054787	-0.591910	-0.754316
H	-7.513876	0.427348	-2.952675
N	-1.595434	-3.802744	-1.994817
N	-3.021839	-2.215940	-2.183370
C	-0.807024	-5.020800	-4.512248
H	-1.296509	-4.056139	-4.342919
C	-1.742440	-5.859311	-5.386488
H	-1.305230	-6.836759	-5.614374
H	-2.700808	-6.031938	-4.886571
H	-1.937372	-5.346856	-6.333991
C	0.521574	-4.743618	-5.218322
H	1.177351	-4.120154	-4.602708
H	1.055539	-5.672695	-5.442849
H	0.345735	-4.224594	-6.165830
C	-3.542362	-1.189475	-4.826864
H	-2.580521	-1.519000	-4.418545
C	-3.283026	0.089100	-5.622865
H	-4.183633	0.435214	-6.139718
H	-2.931613	0.899283	-4.976925
H	-2.521632	-0.094987	-6.386813
C	-4.067983	-2.309557	-5.731066
H	-4.190890	-3.244838	-5.174625
H	-5.042322	-2.043530	-6.154185
H	-3.373787	-2.491854	-6.558027
C	-1.370669	-5.084434	0.556230
H	-1.340829	-3.996578	0.422586
C	-0.541569	-5.409442	1.796534
H	-0.903663	-4.822345	2.645835
H	-0.629718	-6.464268	2.075874
H	0.518483	-5.180861	1.649703
C	-2.833428	-5.489255	0.768008
H	-3.455263	-5.263923	-0.103384
H	-2.918868	-6.561132	0.969624
H	-3.256030	-4.951389	1.623365
C	-4.891283	-2.041409	0.022598
H	-3.891890	-2.488539	0.004650
C	-5.891822	-3.169411	0.283669
H	-5.847305	-3.935562	-0.496572
H	-5.676415	-3.660289	1.237340
H	-6.917764	-2.787954	0.321771
C	-4.908592	-1.005604	1.148024
H	-4.178685	-0.209835	0.970173
H	-5.894492	-0.540556	1.250019
H	-4.668934	-1.483532	2.103014
C	-2.890844	-3.556439	-2.284916
Au	-4.354048	-4.836385	-2.815725
C	-8.478511	-5.697206	-4.237310
C	-7.340152	-4.997099	-3.800135
C	-7.361421	-3.598812	-3.708193

C	-8.513733	-2.901243	-4.050443
C	-9.643468	-3.588751	-4.488319
C	-9.619107	-4.980678	-4.577813
H	-8.409711	-6.792907	-4.243341
H	-6.480137	-3.061578	-3.369947
H	-8.523176	-1.818442	-3.975638
H	-10.542163	-3.040425	-4.757381
H	-10.503576	-5.516353	-4.911720
C	-6.164921	-5.768845	-3.471550
C	-5.561296	-6.855078	-3.312178
C	-4.738801	-8.024381	-3.246234
C	-5.151350	-9.167818	-3.952996
C	-3.555329	-8.055940	-2.492577
C	-4.366860	-10.311934	-3.913665
H	-6.133994	-9.125723	-4.412084
C	-2.787090	-9.212197	-2.461593
H	-3.251511	-7.187875	-1.915633
C	-3.184871	-10.340000	-3.175007
H	-4.693173	-11.197790	-4.450856
H	-1.881146	-9.228351	-1.866247
H	-2.582952	-11.243795	-3.142045
F	-7.625057	-8.253434	-3.558695
C	-5.290341	-7.860659	1.535956
C	-5.451789	-8.693867	0.425193
C	-6.457103	-8.459451	-0.494834
C	-7.346529	-7.373361	-0.347964
C	-7.167372	-6.544136	0.769137
C	-6.155667	-6.782672	1.702328
H	-4.764937	-9.524764	0.291415
H	-6.573293	-9.088731	-1.371530
H	-7.834980	-5.697933	0.917103
H	-6.058531	-6.114881	2.552388
O	-4.250320	-8.171066	2.381397
C	-4.184646	-7.460324	3.589811
H	-5.114188	-7.555334	4.167571
H	-3.360895	-7.892638	4.161518
H	-3.981034	-6.392084	3.430280
N	-8.357403	-7.204669	-1.264330
H	-8.729809	-6.267717	-1.312625
H	-8.150369	-7.630287	-2.201725

Int-3’

C	3.419161	2.464239	0.447205
C	2.504169	3.455927	0.318178
H	4.476856	2.489026	0.655493
H	2.594654	4.527798	0.391414
C	3.365152	-0.016146	0.344098
C	3.636520	-0.531920	1.623373
C	3.713457	-0.680485	-0.837658
C	4.331344	-1.739142	1.691494

C	4.401166	-1.890765	-0.713048
C	4.721438	-2.405830	0.533884
H	4.569665	-2.165329	2.660258
H	4.685575	-2.433902	-1.609239
H	5.263908	-3.343989	0.608533
C	0.045969	3.506034	-0.149166
C	-0.336376	3.837350	-1.455958
C	-0.757556	3.758697	0.971027
C	-1.570258	4.468050	-1.621942
C	-1.982030	4.392528	0.751625
C	-2.382816	4.747485	-0.530624
H	-1.903487	4.730584	-2.621580
H	-2.633449	4.599884	1.595693
H	-3.341344	5.235164	-0.682097
N	2.740178	1.271396	0.259422
N	1.297475	2.837771	0.050521
C	3.369349	-0.146283	-2.211879
H	2.808073	0.784686	-2.086117
C	2.459212	-1.124452	-2.958765
H	2.962525	-2.079664	-3.141509
H	1.549057	-1.326374	-2.385925
H	2.169495	-0.704621	-3.927690
C	4.631642	0.176264	-3.013683
H	5.262020	0.901828	-2.489790
H	5.231511	-0.722751	-3.190877
H	4.364920	0.595946	-3.989005
C	0.518186	3.501316	-2.659992
H	1.439000	3.029825	-2.301255
C	0.915666	4.760100	-3.432746
H	0.037569	5.270799	-3.841821
H	1.447931	5.470162	-2.792010
H	1.568741	4.500092	-4.271929
C	-0.193040	2.487791	-3.559915
H	-0.452659	1.584755	-2.997900
H	-1.116916	2.905267	-3.973912
H	0.453871	2.203127	-4.396516
C	3.131259	0.164840	2.871844
H	3.142330	1.244607	2.681187
C	3.998895	-0.085075	4.102463
H	3.655797	0.543084	4.929872
H	3.931273	-1.124251	4.440962
H	5.051863	0.144786	3.912038
C	1.677729	-0.241316	3.142566
H	1.033717	-0.037645	2.281961
H	1.599025	-1.309058	3.370971
H	1.282366	0.312878	4.000982
C	-0.362812	3.316229	2.365225
H	0.666843	2.945351	2.327448
C	-1.249479	2.154005	2.819631
H	-1.166367	1.306087	2.132262
H	-0.954843	1.814397	3.818097
H	-2.301488	2.457328	2.862603
C	-0.398267	4.472293	3.365174
H	0.241454	5.299148	3.041651
H	-1.412890	4.863269	3.493362
H	-0.050503	4.133584	4.346213
C	1.428120	1.489851	0.005989
Au	-0.073877	0.226487	-0.502414
C	-3.782230	-0.499291	-2.498782
C	-2.908464	-0.148212	-1.453594
C	-3.241023	0.988541	-0.693061
C	-4.406654	1.708824	-0.929228
C	-5.266551	1.334399	-1.959547
C	-4.938483	0.233175	-2.747720
H	-3.548096	-1.357535	-3.117493
H	-2.561276	1.304241	0.093974
H	-4.631349	2.576216	-0.314656
H	-6.174330	1.898705	-2.152911
H	-5.591466	-0.062434	-3.564836
C	-1.669548	-0.888717	-1.139270
C	-1.596633	-2.224079	-1.265314
C	-0.473687	-3.126270	-0.985323
C	-0.303811	-4.281021	-1.761534
C	0.434636	-2.880651	0.053081
C	0.772082	-5.134514	-1.540280
H	-1.018125	-4.498294	-2.548939
C	1.510183	-3.731434	0.269911
H	0.272565	-2.032929	0.710747
C	1.690346	-4.858658	-0.529739
H	0.893874	-6.017228	-2.162049
H	2.207161	-3.510274	1.071840
H	2.533035	-5.522863	-0.358596
F	-2.682541	-2.949249	-1.716504
C	-1.165171	-2.908182	3.241542
C	-1.722132	-1.627667	3.248285
C	-2.796085	-1.320440	2.425514
C	-3.345908	-2.282483	1.565764
C	-2.791967	-3.565159	1.584995
C	-1.715250	-3.880383	2.408818
H	-1.288868	-0.873511	3.897913
H	-3.203277	-0.312543	2.428759
H	-3.190143	-4.325216	0.918386
H	-1.299782	-4.880489	2.365020
O	-0.088195	-3.107506	4.062234
C	0.521382	-4.375254	4.014129
H	0.879315	-4.613943	3.004091
H	1.371260	-4.333345	4.697703
H	-0.163009	-5.168403	4.343665
N	-4.433347	-1.987188	0.740520
H	-4.505589	-0.996949	0.541111
H	-4.376107	-2.480016	-0.142318

TS-4’

C	-0.609466	-1.294918	-1.929372
C	-1.591931	-0.389816	-2.153228
H	0.447013	-1.174856	-1.750349
H	-1.572693	0.686508	-2.215312
C	-0.505322	-3.774077	-1.755155
C	-0.315759	-4.217005	-0.436393
C	-0.025474	-4.469941	-2.875615
C	0.369007	-5.420845	-0.260333
C	0.647803	-5.670539	-2.640382
C	0.839960	-6.144038	-1.348926
H	0.533304	-5.796246	0.745481
H	1.029269	-6.239527	-3.483510
H	1.366259	-7.080801	-1.189113
C	-4.013187	-0.461896	-2.633418
C	-4.236787	-0.078128	-3.965276
C	-4.914812	-0.184971	-1.592765
C	-5.396822	0.650478	-4.234345
C	-6.060325	0.540880	-1.923013
C	-6.294971	0.964653	-3.223934
H	-5.603579	0.963070	-5.254174
H	-6.787811	0.763180	-1.149551
H	-7.198617	1.520203	-3.455671
N	-1.207128	-2.538700	-1.964114
N	-2.762673	-1.103406	-2.321874
C	-0.184136	-3.953915	-4.293705
H	-0.760196	-3.023447	-4.255082
C	-0.964821	-4.932517	-5.173829
H	-0.443865	-5.891603	-5.267348
H	-1.962022	-5.125553	-4.768612
H	-1.083981	-4.521661	-6.181806
C	1.181118	-3.624285	-4.906974
H	1.730716	-2.898220	-4.299439
H	1.803536	-4.520934	-4.997206
H	1.056555	-3.204259	-5.910351
C	-3.299917	-0.452729	-5.098916
H	-2.452593	-1.003865	-4.676818
C	-2.735987	0.784175	-5.803318
H	-3.530860	1.374698	-6.270784
H	-2.204291	1.438366	-5.104772
H	-2.036612	0.488852	-6.592344
C	-3.998448	-1.389043	-6.090864
H	-4.394754	-2.276940	-5.589239
H	-4.834892	-0.886956	-6.588926
H	-3.297238	-1.714228	-6.866942
C	-0.785795	-3.421831	0.767723
H	-1.417734	-2.602491	0.408880
C	0.410767	-2.799448	1.496630
H	0.070488	-2.192477	2.341930
H	1.078414	-3.574810	1.887817
H	1.000915	-2.158155	0.833850
C	-1.636241	-4.259872	1.723251
H	-2.502994	-4.692095	1.217287
H	-1.060744	-5.079677	2.166012
H	-1.999549	-3.635304	2.545712
C	-4.671831	-0.624038	-0.161083
H	-3.937056	-1.436513	-0.179945
C	-5.935719	-1.181719	0.499144
H	-6.409886	-1.959229	-0.113233
H	-5.675588	-1.614964	1.471251
H	-6.673164	-0.393683	0.688495
C	-4.077267	0.526724	0.659554
H	-3.135224	0.888113	0.233168
H	-4.771999	1.372932	0.699762
H	-3.881913	0.203340	1.687538
C	-2.538398	-2.434554	-2.206128
Au	-3.860781	-3.958868	-2.286152
C	-5.336751	-5.574447	-2.469062
C	-4.642815	-6.283902	-1.499151
C	-5.424184	-6.027117	-3.883841
C	-6.598574	-5.726923	-4.591400
C	-4.404390	-6.719461	-4.560403
C	-6.753700	-6.115698	-5.917059
H	-7.392193	-5.176217	-4.097066
C	-4.560025	-7.095989	-5.890091
H	-3.482523	-6.963975	-4.046137
C	-5.735465	-6.799963	-6.575814
H	-7.676843	-5.871966	-6.435181
H	-3.755527	-7.628001	-6.391346
H	-5.854886	-7.098222	-7.613756
C	-4.725259	-6.162158	-0.043334
C	-4.047519	-7.090006	0.764266
C	-5.519666	-5.169600	0.559107
C	-4.160816	-7.028345	2.147662
H	-3.441210	-7.860339	0.301755
C	-5.618474	-5.119430	1.943446
H	-6.072049	-4.460191	-0.063570
C	-4.943803	-6.041939	2.743547
H	-3.636518	-7.754893	2.761949
H	-6.239264	-4.354080	2.401186
H	-5.032940	-5.996058	3.825387
F	-3.890579	-7.338057	-1.871442
C	-9.252875	-2.345264	-4.965533
C	-10.003141	-3.159174	-4.110889
C	-9.452042	-3.659704	-2.944672
C	-8.109113	-3.382340	-2.552917
C	-7.393071	-2.522629	-3.426816
C	-7.941762	-2.031095	-4.607970
H	-11.028035	-3.392529	-4.387806
H	-10.054470	-4.306746	-2.308737
H	-6.379737	-2.241357	-3.150676

H	-7.333810	-1.385793	-5.234112
N	-7.497640	-3.918074	-1.465027
H	-8.191018	-4.452692	-0.939526
H	-6.220724	-4.936519	-2.066682
O	-9.890827	-1.901876	-6.099490
C	-9.141307	-1.113428	-6.977903
H	-9.794479	-0.876895	-7.821148
H	-8.810680	-0.172770	-6.511192
H	-8.252040	-1.642631	-7.352347

TS-5’

C	-1.165790	-1.244168	-1.232387
C	-2.237813	-0.416064	-1.263122
H	-0.118584	-1.053211	-1.061464
H	-2.321735	0.649996	-1.125529
C	-0.856600	-3.709056	-1.578346
C	-0.616555	-4.452420	-0.414809
C	-0.398327	-4.094849	-2.845195
C	0.125965	-5.626647	-0.547629
C	0.339047	-5.277828	-2.924667
C	0.599492	-6.034962	-1.789246
H	0.326958	-6.233782	0.329995
H	0.705507	-5.612748	-3.890678
H	1.172354	-6.954022	-1.872846
C	-4.688227	-0.744839	-1.686511
C	-5.168521	-0.542401	-2.987749
C	-5.470880	-0.546656	-0.538849
C	-6.489506	-0.106701	-3.119815
C	-6.780841	-0.106403	-0.726264
C	-7.284428	0.114162	-2.003300
H	-6.895233	0.062535	-4.112876
H	-7.418629	0.053032	0.137172
H	-8.308196	0.456329	-2.128165
N	-1.645994	-2.520629	-1.476860
N	-3.343159	-1.209158	-1.522503
C	-0.702434	-3.293406	-4.094190
H	-1.213251	-2.373932	-3.789784
C	-1.650728	-4.060227	-5.019818
H	-1.193324	-4.996016	-5.359574
H	-2.595295	-4.302235	-4.522338
H	-1.879476	-3.458666	-5.906003
C	0.579635	-2.883117	-4.821122
H	1.250501	-2.321417	-4.163492
H	1.126928	-3.755858	-5.192497
H	0.339174	-2.253706	-5.683809
C	-4.310125	-0.751349	-4.218655
H	-3.347128	-1.159527	-3.896280
C	-4.034179	0.588181	-4.907460
H	-4.961512	1.042840	-5.272488
H	-3.558833	1.299729	-4.224393
H	-3.372080	0.443896	-5.767393
C	-4.930253	-1.763612	-5.184121
H	-5.060086	-2.749862	-4.723163
H	-5.903316	-1.416723	-5.550012
H	-4.278780	-1.882740	-6.056949
C	-1.190287	-4.044923	0.926790
H	-1.556005	-3.016115	0.839704
C	-0.142136	-4.066038	2.039002
H	-0.576882	-3.694096	2.972107
H	0.224054	-5.079787	2.231305
H	0.719270	-3.439193	1.788941
C	-2.391850	-4.929919	1.272174
H	-3.152294	-4.867249	0.486793
H	-2.085325	-5.977000	1.372813
H	-2.843111	-4.613451	2.218503
C	-4.947454	-0.880610	0.843184
H	-3.852933	-0.830124	0.810505
C	-5.335390	-2.318911	1.202954
H	-4.943989	-3.025027	0.464208
H	-4.939154	-2.588409	2.188221
H	-6.424031	-2.433893	1.224643
C	-5.416745	0.101364	1.914792
H	-5.191365	1.136935	1.641642
H	-6.494264	0.023439	2.092494
H	-4.918650	-0.118561	2.864048
C	-2.987417	-2.511005	-1.660220
Au	-4.200194	-3.986447	-2.102324
F	-5.179406	-4.842353	-3.999049
C	-8.383958	-4.669793	0.820059
C	-7.346781	-5.338891	0.185125
C	-6.918949	-4.945328	-1.085303
C	-7.560595	-3.873769	-1.708644
C	-8.602466	-3.202094	-1.078095
C	-9.016726	-3.591804	0.196933
H	-8.717562	-4.969139	1.808662
H	-6.857327	-6.170506	0.686368
H	-7.222076	-3.552907	-2.690108
H	-9.066876	-2.366779	-1.588062
O	-10.013714	-2.989045	0.903669
C	-10.645138	-1.882071	0.306366
H	-11.145822	-2.155522	-0.631538
H	-11.391722	-1.531931	1.020555
H	-9.933355	-1.070515	0.102779
N	-5.809012	-5.552492	-1.715222
H	-5.536630	-6.435306	-1.303406
H	-5.834562	-5.571309	-2.766975
